# Transcriptome profiling and pathway analysis of genes expressed differentially in participants with or without a positive response to topiramate treatment for methamphetamine addiction

**DOI:** 10.1186/s12920-014-0065-x

**Published:** 2014-12-12

**Authors:** Ming D Li, Ju Wang, Tianhua Niu, Jennie Z Ma, Chamindi Seneviratne, Nassima Ait-Daoud, Jim Saadvandi, Rana Morris, David Weiss, Jan Campbell, William Haning, David J Mawhinney, Denis Weis, Michael McCann, Christopher Stock, Roberta Kahn, Erin Iturriaga, Elmer Yu, Ahmed Elkashef, Bankole A Johnson

**Affiliations:** Department of Psychiatry and Neurobehavioral Sciences, University of Virginia, Charlottesville, USA; Information Management Consultants, Reston, USA; Department of Psychiatry, University of Missouri, Kansas City, USA; Pacific Addiction Research Center, Honolulu, USA; South Bay Treatment Center, San Diego, USA; Lutheran Hospital Office of Research, Des Moines, USA; Matrix Institute on Addictions, West Los Angeles, USA; Department of Veterans Affairs, Salt Lake City Health Care System, Salt Lake City, USA; Division of Pharmacotherapies and Medical Consequences of Drug Abuse, NIDA, Bethesda, USA; Veterans Administration Medical Center, Philadelphia, USA; Department of Psychiatry, University of Maryland, Baltimore, USA; Department of Veterans Affairs Cooperative Studies Program Coordination Center, Perry Point, USA

**Keywords:** Topiramate, Pharmacogenetics, Genes, Pathways, Transcriptome, Addiction treatment, Methamphetamine addiction

## Abstract

**Background:**

Developing efficacious medications to treat methamphetamine dependence is a global challenge in public health. Topiramate (TPM) is undergoing evaluation for this indication. The molecular mechanisms underlying its effects are largely unknown. Examining the effects of TPM on genome-wide gene expression in methamphetamine addicts is a clinically and scientifically important component of understanding its therapeutic profile.

**Methods:**

In this double-blind, placebo-controlled clinical trial, 140 individuals who met the DSM-IV criteria for methamphetamine dependence were randomized to receive either TPM or placebo, of whom 99 consented to participate in our genome-wide expression study. The RNA samples were collected from whole blood for 50 TPM- and 49 placebo-treated participants at three time points: baseline and the ends of weeks 8 and 12. Genome-wide expression profiles and pathways of the two groups were compared for the responders and non-responders at Weeks 8 and 12. To minimize individual variations, expression of all examined genes at Weeks 8 and 12 were normalized to the values at baseline prior to identification of differentially expressed genes and pathways.

**Results:**

At the single-gene level, we identified 1054, 502, 204, and 404 genes at nominal P values < 0.01 in the responders vs. non-responders at Weeks 8 and 12 for the TPM and placebo groups, respectively. Among them, expression of 159, 38, 2, and 21 genes was still significantly different after Bonferroni corrections for multiple testing. Many of these genes, such as *GRINA*, *PRKACA*, *PRKCI*, *SNAP23*, and *TRAK2*, which are involved in glutamate receptor and GABA receptor signaling, are direct targets for TPM. In contrast, no TPM drug targets were identified in the 38 significant genes for the Week 8 placebo group. Pathway analyses based on nominally significant genes revealed 27 enriched pathways shared by the Weeks 8 and 12 TPM groups. These pathways are involved in relevant physiological functions such as neuronal function/synaptic plasticity, signal transduction, cardiovascular function, and inflammation/immune function.

**Conclusion:**

Topiramate treatment of methamphetamine addicts significantly modulates the expression of genes involved in multiple biological processes underlying addiction behavior and other physiological functions.

**Electronic supplementary material:**

The online version of this article (doi:10.1186/s12920-014-0065-x) contains supplementary material, which is available to authorized users.

## Background

Methamphetamine (METH), an N-methyl derivative of amphetamine commonly abused recreationally, is a powerfully addictive psychostimulant that affects the central nervous system (CNS) dramatically [1]. Dependence on the drug has risen to an epidemic level worldwide [2], and in 2007, 529,000 Americans (ca. 0.2% of the US population) were METH users [3].

METH induces long-term changes in behavior, including sensitization and dependence [4,5], as well as deficits in cognitive function [6-8], and causes psychiatric symptoms such as hallucinations and delusions [9]. Its use and abuse have been associated with several significant health risks, including cardiac dysrhythmia, stroke, high blood pressure, hyperthermia, and CNS abnormalities [10,11] that are thought to reflect changes in the signaling and metabolism of neurotransmitters such as dopamine, serotonin, and glutamate [10,12-16].

Unfortunately, no efficacious medication for METH dependence has been developed to date [17]. There is a great need, not only for novel treatments, but for understanding of its molecular mechanisms. Topiramate (TPM), a sulfamate-substituted derivative of the monosaccharide D-fructose [18], has been efficacious in the treatment of alcohol dependence [19] and in promoting smoking cessation among alcohol-dependent smokers [20]. A preliminary study suggests that it also may be useful for treating cocaine dependence [21]. These therapeutic effects have been attributed to its hypothesized potential to reduce the release of cortico-mesolimbic dopamine, the neurotransmitter primarily responsible for the acquisition and maintenance of drug-seeking behaviors for the majority of abused drugs, including amphetamines. Thus, TPM might be efficacious for treating METH dependence [22]. However, the effects of TPM on METH-dependent subjects seem to be complex. Whereas earlier studies have not uncovered any deleterious interactions between TPM and METH with respect to cognitive performance, attention, or concentration, TPM tends to enhance METH-induced increases in attention and decrease perceptual-motor function [22]. Also, TPM accentuates markedly the positive subjective effects of METH, although not craving or reinforcement [23]. Although several hypotheses have been offered on the basis of clinical laboratory studies for the effects of TPM on METH dependence [22-25], the molecular mechanisms remain unclear.

In a recently completed double-blind, multi-center, placebo-controlled clinical trial of the treatment of METH dependence with TPM, mixed results were obtained [26]. Thus, although TPM did not increase abstinence from METH use, it significantly reduced urine METH concentrations and observer-rated severity of dependence [26]. From this trial, a genome-wide expression analysis was conducted on RNA extracted from the blood of participants, with the goal of identifying differentially expressed genes and pathways in the responders and non-responders. Such a global gene expression investigation not only provides evidence at the molecular level explaining the interaction of TPM and METH but also may help us to evaluate the pharmacological effect of TPM on METH dependence.

## Results

### Grouping study participants used for transcriptome analysis

On the basis of 209 chips that passed quality control, 49 participants in the placebo group and 50 in the TPM group were included. According to the criteria for primary efficacy outcome [26] (also see Methods), these participants were classified as responders or non-responders. For these participants, only 43 had a gene expression study at all three time points, 27 and 24 of which could be classified as responders or non-responders, respectively. For the other 16 participants, either no valid urine samples were tested or the patients were excluded for other reasons at Weeks 8 and 12 (see Additional file 1: Figure S1). To increase the sample size, we included some participants having valid gene expression data at Week 8 but not at Week 0 (baseline) among the Week 8 samples, as well as those participants with valid gene expression data at both Weeks 8 and 12 but not at baseline. Finally, we identified 5 responders and 17 non-responders in the Week 8 TPM group, 4 responders and 17 non-responders in the Week 8 placebo group (see Additional file 1: Figure S1A), 6 responders and 11 non-responders in the Week 12 TPM group, and 2 responders and 13 non-responders in the Week 12 placebo group (see Additional file 1: Figure S1B).

### Identification of genes differentially expressed in responders and non-responders at Weeks 8 and 12

At a significance level of 0.01, we identified 1,054 (FDR: 0.009 ± 0.010; range <1 × 10^−5^ - 0.035), 502 (FDR: 0.027 ± 0.021; range: <1 × 10^−5^ - 0.070), 204 (FDR: 0.113 ± 0.034; range: 0.003 - 0.160), and 404 (FDR: 0.033 ± 0.024; range: <1 × 10^−5^ - 0.084) differentially expressed genes between responders and non-responders for the Week 8 TPM, Week 8 placebo, Week 12 TPM, and Week 12 placebo groups, respectively (see Additional file 2: Tables S1-S4 for details). Of these four groups, the Week 8 TPM group had the lowest FDR. To take into account the number of genes tested in the four groups, 159, 38, 2, and 21 genes, respectively, remained significant at Bonferroni-corrected P values < 0.05.

In the Week 8 TPM group, 159 genes were significantly changed with a Bonferroni-corrected P value of < 0.05, with 97 being up-regulated and 62 down-regulated comparing positive and negative responders. Importantly, none of these 159 genes overlapped with the 38 genes detected in the Week 8 placebo group at a Bonferroni-corrected P value of 0.05 (Additional file 2: Table S2). Tables 1 and 2 show, respectively, the representative up-regulated and down-regulated genes whose functions are related to cell adhesion/motion, nervous system development and function/synaptic plasticity, signal transduction, ubiquitination/intracellular protein transport, mitochondrial function/metabolism and energy pathways, and immune system function categories.

**Table 1:** **A list of 48 representative genes significantly up**-**regulated in week 8 topiramate group** ^**a**^

**Gene symbol**	**Gene name**	**Week 8 TPM**		
		**FC** ± **SD** ^**b**^	**P Value** ^**c**^	**FDR** ^**d**^
**Cell adhesion** **/** **Motion**				
*CD164*	CD164 molecule, sialomucin	2.67 ± 0.38	1.00 × 10^−6^	5.07 × 10^−5^
*ITGA4*	Integrin, alpha 4 (antigen CD49D, alpha 4 subunit of VLA-4 receptor)	2.24 ± 0.27	4.00 × 10^−6^	1.31 × 10^−4^
*ITGB1*	Integrin, beta 1 (fibronectin receptor, beta polypeptide, antigen CD29 includes MDF2, MSK12)	2.20 ± 0.25	1.10 × 10^−5^	2.64 × 10^−4^
*SCYL2*	SCY1-like 2 (S. cerevisiae)	2.45 ± 0.30	4.00 × 10^−6^	1.31 × 10^−4^
**Synaptic plasticity and nervous system development** **/** **function**				
*DLG1*	Discs, large homolog 1 (Drosophila)	1.66 ± 0.12	2.00 × 10^−6^	8.22 × 10^−5^
*GDI2*	GDP dissociation inhibitor 2	1.63 ± 0.09	<1.00 × 10^−6^	<1.00 × 10^−5^
*HIF1A*	Hypoxia-inducible factor 1, alpha subunit (basic helix-loop-helix transcription factor)	2.22 ± 0.26	4.00 × 10^−6^	1.31 × 10^−4^
*SCFD1*	Sec1 family domain containing 1	1.63 ± 0.12	3.00 × 10^−6^	1.09 × 10^−4^
*SNAP23*	Synaptosomal-associated protein, 23kDa	1.95 ± 0.19	2.00 × 10^−6^	8.22 × 10^−5^
*TRAK2*	Trafficking protein, kinesin binding 2	1.44 ± 0.07	<1.00 × 10^−6^	<1.00 × 10^−5^
*ZFR*	Zinc finger RNA binding protein	2.14 ± 0.22	1.00 × 10^−6^	5.07 × 10^−5^
**Signal transduction**				
*AKAP11*	A kinase (PRKA) anchor protein 11	2.23 ± 0.29	1.10 × 10^−5^	2.60 × 10^−4^
*CCNYL1*	Cyclin Y-like 1	1.62 ± 0.10	3.00 × 10^−6^	1.09 × 10^−4^
*ERBB2IP*	Erbb2 interacting protein	2.28 ± 0.23	<1.00 × 10^−6^	<1.00 × 10^−5^
*FGFR1OP2*	FGFR1 oncogene partner 2	1.57 ± 0.11	4.00 × 10^−6^	1.31 × 10^−4^
*MAPK1IP1L*	Mitogen-activated protein kinase 1 interacting protein 1-like	1.39 ± 0.06	<1.00 × 10^−6^	<1.00 × 10^−5^
*PIK3AP1*	Phosphoinositide-3-kinase adaptor protein 1	1.70 ± 0.14	5.00 × 10^−6^	1.54 × 10^−4^
*PIK3R1*	Phosphoinositide-3-kinase, regulatory subunit 1 (alpha)	2.04 ± 0.23	7.00 × 10^−6^	1.92 × 10^−4^
*PIP5K3*	Phosphatidylinositol-3-phosphate/phosphatidylinositol 5-kinase, type III	1.95 ± 0.20	1.00 × 10^−5^	2.53 × 10^−4^
*PTEN*	Phosphatase and tensin homolog	2.01 ± 0.10	<1.00 × 10^−6^	<1.00 × 10^−5^
*RABEP1*	Rabaptin, RAB GTPase binding effector protein 1	1.42 ± 0.08	5.00 × 10^−6^	1.54 × 10^−4^
*RAP2A*	RAP2A, member of RAS oncogene family	1.75 ± 0.15	8.00 × 10^−6^	2.13 × 10^−4^
*RAP2C*	RAP2C, member of RAS oncogene family	1.84 ± 0.16	3.00 × 10^−6^	1.09 × 10^−4^
*SKAP2*	Src kinase associated phosphoprotein 2	1.76 ± 0.15	5.00 × 10^−6^	1.54 × 10^−4^
*SOS2*	Son of sevenless homolog 2 (Drosophila)	2.51 ± 0.25	<1.00 × 10^−6^	<1.00 × 10^−5^
*TGFBR2*	Transforming growth factor, beta receptor II (70/80kDa)	1.49 ± 0.09	8.00 × 10^−6^	2.13 × 10^−4^
*TOB1*	Transducer of ERBB2, 1	2.28 ± 0.27	1.00 × 10^−6^	5.07 × 10^−5^
*ZFAND6*	Zinc finger, AN1-type domain 6	2.25 ± 0.20	<1.00 × 10^−6^	<1.00 × 10^−5^
**Ubiquitination**/**Intracellular protein transport**				
*CUL5*	Cullin 5	2.25 ± 0.29	6.00 × 10^−6^	1.72 × 10^−4^
*FBXL5*	F-box and leucine-rich repeat protein 5	1.69 ± 0.09	<1.00 × 10^−6^	<1.00 × 10^−5^
*FBXO28*	F-box protein 28	1.45 ± 0.08	5.00 × 10^−6^	1.54 × 10^−4^
*PCNP*	PEST proteolytic signal containing nuclear protein	2.54 ± 0.35	3.00 × 10^−6^	1.09 × 10^−4^
*PSMD12*	Proteasome (prosome, macropain) 26S subunit, non-ATPase, 12	1.77 ± 0.12	<1.00 × 10^−6^	<1.00 × 10^−5^
*RNF11*	Ring finger protein 11	3.29 ± 0.39	<1.00 × 10^−6^	<1.00 × 10^−5^
*RNF149*	Ring finger protein 149	1.51 ± 0.10	8.00 × 10^−6^	2.13 × 10^−4^
*SEC62*	SEC62 homolog (S. cerevisiae)	1.69 ± 0.12	<1.00 × 10^−6^	<1.00 × 10^−5^
*SMURF2*	SMAD specific E3 ubiquitin protein ligase 2	1.64 ± 0.10	<1.00 × 10^−6^	<1.00 × 10^−5^
*SRP54*	Signal recognition particle 54kDa	1.56 ± 0.10	3.00 × 10^−6^	1.09 × 10^−4^
*UBE2D1*	Ubiquitin-conjugating enzyme E2D 1 (UBC4/5 homolog, yeast)	2.41 ± 0.30	2.00 × 10^−6^	8.22 × 10^−5^
*XPO1*	Exportin 1 (CRM1 homolog, yeast)	1.98 ± 0.18	<1.00 × 10^−6^	<1.00 × 10^−5^
*YME1L1*	YME1-like 1 (S. cerevisiae)	1.69 ± 0.11	<1.00 × 10^−6^	<1.00 × 10^−5^
**Mitochondrial function** **/** **Metabolism and energy pathways**				
*ATP8A1*	ATPase, aminophospholipid transporter (APLT), class I, type 8A, member 1	2.11 ± 0.24	9.00 × 10^−6^	2.38 × 10^−4^
*GALNT7*	UDP-N-acetyl-alpha-D-galactosamine:polypeptide N-acetylgalactosaminyltransferase 7 (GalNAc-T7)	1.87 ± 0.17	3.00 × 10^−6^	1.09 × 10^−4^
*MAN1A1*	Mannosidase, alpha, class 1A, member 1	2.26 ± 0.28	6.00 × 10^−6^	1.72 × 10^−4^
*NUDT5*	Nudix (nucleoside diphosphate linked moiety X)-type motif 5	1.34 ± 0.04	<1.00 × 10^−6^	<1.00 × 10^−5^
*PGK1*	Phosphoglycerate kinase 1	1.35 ± 0.06	6.00 × 10^−6^	1.72 × 10^−4^
*SLC25A46*	Solute carrier family 25, member 46	2.20 ± 0.25	3.00 × 10^−6^	1.09 × 10^−4^
*TXNL1*	Thioredoxin-like 1	1.49 ± 0.08	1.00 × 10^−6^	5.07 × 10^−5^

^a^Genes are selected from a total of 97 significantly up-regulated genes with Bonferroni-corrected P Values < 0.05 (i.e., 0.05/3698 genes = 1.35 × 10^−5^).

^b^FC, denoting fold change, is defined as the reciprocal of the ratio of the expression values of Positive Responders over Negative Responders; SD, standard deviation.

^c^P Value was calculated using the ordinary Student’s *t* test for each gene.

^d^FDR, denoting false discovery rate, was estimated by the Benjamini-Hochberg (BH) method.

**Table 2:** **A List of 45 representative genes significantly down**-**regulated in week 8 topiramate group** ^**a**^

**Gene symbol**	**Gene name**	**Week 8 TPM**		
		**FC** ± **SD** ^**b**^	**P Value** ^**c**^	**FDR** ^**d**^
**Cell adhesion** **/** **Motion**				
*CDC2L2*	Cell division cycle 2-like 2	−1.39 ± 0.07	1.10 × 10^−5^	2.64 × 10^−4^
*CDC42EP2*	CDC42 effector protein (Rho GTPase binding) 2	−2.01 ± 0.19	1.00 × 10^−6^	5.07 × 10^−5^
*EMILIN2*	Elastin microfibril interfacer 2	−1.41 ± 0.05	<1.00 × 10^−6^	<1.00 × 10^−5^
*JAK3*	Janus kinase 3 (a protein tyrosine kinase, leukocyte)	−1.68 ± 0.11	1.00 × 10^−6^	5.07 × 10^−5^
*TUBB2C*	Tubulin, beta 2C	−1.39 ± 0.06	2.00 × 10^−6^	8.22 × 10^−5^
**Nervous system development and function** **/** **synaptic plasticity**				
*ADAT1*	Adenosine deaminase, tRNA-specific 1	−1.61 ± 0.08	<1.00 × 10^−6^	<1.00 × 10^−5^
*CIRBP*	Cold inducible RNA binding protein	−1.74 ± 0.13	1.00 × 10^−6^	5.07 × 10^−5^
*DGCR14*	3-phosphoinositide dependent protein kinase-1	−1.45 ± 0.04	<1.00 × 10^−6^	<1.00 × 10^−5^
*FKBP4*	FK506 binding protein 4, 59 kDa	−1.49 ± 0.10	6.00 × 10^−6^	1.72 × 10^−4^
*NAPA*	N-ethylmaleimide-sensitive factor attachment protein, alpha	−2.04 ± 0.21	1.00 × 10^−6^	5.07 × 10^−5^
*P2RX1*	Purinergic receptor P2X, ligand-gated ion channel, 1	−1.72 ± 0.13	2.00 × 10^−6^	8.22 × 10^−5^
*PRKACA*	Solute carrier family 1 (glial high affinity glutamate transporter), member 3	−1.69 ± 0.15	1.10 × 10^−5^	2.64 × 10^−4^
*SHC1*	SHC (Src homology 2 domain containing) transforming protein 1	−1.23 ± 0.04	3.00 × 10^−6^	1.09 × 10^−4^
*TRIM8*	Tripartite motif-containing 8	−1.45 ± 0.06	4.00 × 10^−6^	1.31 × 10^−4^
**Signaling transduction**				
*ARHGEF2*	Rho/rac guanine nucleotide exchange factor (GEF) 2	−1.62 ± 0.11	2.00 × 10^−6^	8.22 × 10^−5^
*BCR*	Breakpoint cluster region	−1.43 ± 0.06	<1.00 × 10^−6^	<1.00 × 10^−5^
*GRINA*	Glutamate receptor, ionotropic, N-methyl D-aspartate-associated protein 1	−2.49 ± 0.28	<1.00 × 10^−6^	<1.00 × 10^−5^
*LIMK2*	LIM domain kinase 2	−3.05 ± 0.44	4.00 × 10^−6^	1.31 × 10^−4^
*PHPT1*	Phosphohistidine phosphatase 1	−1.33 ± 0.06	7.00 × 10^−6^	1.92 × 10^−4^
*PLCB2*	Phospholipase C, beta 2	−1.93 ± 0.13	<1.00 × 10^−6^	<1.00 × 10^−5^
*RHOT2*	Ras homolog gene family, member T2	−1.46 ± 0.05	<1.00 × 10^−6^	<1.00 × 10^−5^
**Ubiquitination** **/** **Intracellular protein transport**				
*AP2A1*	Adaptor-related protein complex 2, alpha 1 subunit	−2.04 ± 0.20	1.10 × 10^−5^	2.64 × 10^−4^
*NPEPL1*	Aminopeptidase-like 1	−1.26 ± 0.03	<1.00 × 10^−6^	<1.00 × 10^−5^
*SHARPIN*	SHANK-associated RH domain interactor	−2.13 ± 0.26	1.00 × 10^−5^	2.53 × 10^−4^
*TRAPPC5*	Trafficking protein particle complex 5	−1.58 ± 0.11	6.00 × 10^−6^	1.72 × 10^−4^
*UBE2M*	Ubiquitin-conjugating enzyme E2M (UBC12 homolog, yeast)	−1.70 ± 0.12	1.00 × 10^−6^	5.07 × 10^−5^
*UBXN6*	UBX domain protein 6	−2.40 ± 0.29	2.00 × 10^−6^	8.22 × 10^−5^
*USP4*	Ubiquitin specific peptidase 4 (proto-oncogene)	−1.46 ± 0.06	2.00 × 10^−6^	8.22 × 10^−5^
*WBP2*	WW domain binding protein 2	−1.85 ± 0.18	4.00 × 10^−6^	1.31 × 10^−4^
**Mitochondrial function** **/** **metabolism and energy pathways**				
*ATAD3B*	ATPase family, AAA domain containing 3B	−1.47 ± 0.07	<1.00 × 10^−6^	<1.00 × 10^−5^
*B4GALT3*	UDP-Gal:betaGlcNAc beta 1,4- galactosyltransferase, polypeptide 3	−1.26 ± 0.04	1.00 × 10^−6^	5.07 × 10^−5^
*COASY*	Coenzyme A synthase	−1.29 ± 0.06	1.20 × 10^−5^	2.79 × 10^−4^
*COX5B*	Cytochrome c oxidase subunit Vb	−1.44 ± 0.09	1.20 × 10^−5^	2.79 × 10^−4^
*GANAB*	Glucosidase, alpha; neutral AB	−1.63 ± 0.08	<1.00 × 10^−6^	<1.00 × 10^−5^
*MRPL37*	Mitochondrial ribosomal protein L37	−1.30 ± 0.04	<1.00 × 10^−6^	<1.00 × 10^−5^
*NAGK*	N-acetylglucosamine kinase	−1.30 ± 0.05	4.00 × 10^−6^	1.31 × 10^−4^
*NDUFV3*	NADH dehydrogenase (ubiquinone) flavoprotein 3, 10 kDa	−1.63 ± 0.08	<1.00 × 10^−6^	<1.00 × 10^−5^
*PFKL*	Phosphofructokinase, liver	−1.53 ± 0.10	3.00 × 10^−6^	1.09 × 10^−4^
*UROD*	Uroporphyrinogen decarboxylase	−1.35 ± 0.06	5.00 × 10^−6^	1.54 × 10^−4^
**Immune system function**				
*CISH*	Cytokine inducible SH2-containing protein	−1.34 ± 0.05	1.00 × 10^−6^	5.07 × 10^−5^
*HLA*-*E*	Major histocompatibility complex, class I, E	−1.25 ± 0.04	<1.00 × 10^−6^	<1.00 × 10^−5^
*JMJD3*	Jumonji domain containing 3, histone lysine demethylase	−2.03 ± 0.16	<1.00 × 10^−6^	<1.00 × 10^−5^
*MSRB2*	Methionine sulfoxide reductase B2	−1.68 ± 0.13	2.00 × 10^−6^	8.22 × 10^−5^
*OAS1*	2′,5′-oligoadenylate synthetase 1, 40/46 kDa	−1.84 ± 0.15	1.00 × 10^−6^	5.07 × 10^−5^
*TAPBP*	TAP binding protein (tapasin)	−1.60 ± 0.06	<1.00 × 10^−6^	<1.00 × 10^−5^

^a^Genes are selected from a total of 62 down-regulated genes with Bonferroni-corrected P Values < 0.05 (i.e., 0.05/3698 genes = 1.35 × 10^−5^).

^b^FC, denoting fold change, is defined as the ratio of the expression values of Positive Responders over Negative Responders; SD, standard deviation.

^c^P Value was calculated using the ordinary Student’s *t* test for each gene.

^d^FDR, denoting false discovery rate, was estimated by the Benjamini-Hochberg (BH) method.

In the Week 12 TPM group, we detected only two genes, *ITCH* and *MKNK2*, whose expression remained significant after Bonferroni correction for multiple testing (Additional file 2: Table S3). Both were down-regulated by TPM and are in the nervous system development and function/synaptic plasticity category. Although the exact reason is unknown, we suspect that the small size of the Week 12 TPM group might have contributed. None of them overlapped with those 21 genes changed by placebo at Week 12 with Bonferroni-corrected P values < 0.05 (Additional file 2: Table S4).

#### Pathways identified by IPA

The differentially expressed genes were subjected to pathway analysis using the IPA. A total of 114, 41, 54, and 25 pathways with at least three genes overexpressed were enriched at a nominal P value of < 0.05 between responders and non-responders for Week 8 TPM, Week 8 placebo, Week 12 TPM, and Week 12 placebo, respectively. Among these pathways, 21 significantly enriched pathways with an FDR of < 0.05 at either time point or FDR < 0.10 at both time points were shared exclusively by the Week 8 and Week 12 TPM groups (Table 3), suggesting they are more likely to be the pathways related to the treatment effect of TPM in METH-dependent subjects. No significantly enriched pathways were shared exclusively by the Week 8 and Week 12 placebo groups, with FDRs < 0.10 at both time points. Although 163, 149, 137, and 120 pathways were detected for the Week 8 TPM, Week 8 placebo, Week 12 TPM, and Week 12 placebo groups, respectively, at a significance level of 0.05, only 46, 5, 6, and 0 pathways remained significant after Bonferroni correction for multiple testing. A comparison of these significant pathways after correction for multiple testing revealed that only two pathways (i.e., B-cell receptor signaling and renin-angiotensin signaling) were shared exclusively by the Week 8 and Week 12 TPM groups, and no pathways were shared by the Week 8 and Week 12 placebo groups.

**Table 3:** **Significantly enriched pathways detected exclusively in week 8 and week 12 topiramate groups** (**n** = **27**)^**a**^

	**Week 8 TPM**			**Week 12 TPM**		
**Pathway name** ^**b**^	**No. genes**	**P Value**	**FDR** ^**c**^	**No. genes**	**P Value**	**FDR** ^**c**^
**Neuronal function** **/** **Synaptic plasticity** **(** **n** **=** **8** **)**						
Alpha-adrenergic signaling^a^	17	0.025	0.042	10	0.0036	0.029
Ephrin receptor signaling^a^	36	1.70 × 10^−4^	7.18 × 10^−4^	17	5.75 × 10^−4^	0.0095
ErbB signaling pathway^b^	18	0.0026	0.0081	8	0.0078	0.035
FGF signaling^a^	18	0.0040	0.0086	8	0.017	0.074
GnRH signaling pathway^b^	20	0.0017	0.0060	11	0.0012	0.013
mTOR signaling pathway^b^	15	4.47 × 10^−5^	3.78 × 10^−4^	5	0.033	0.090
Neurotrophin/TRK signaling^a^	17	9.33 × 10^−4^	0.0027	7	0.017	0.074
Synaptic long term Potentiation^a,b^	*29*	*6.17* × *10* ^−*6*^	*7.44* × *10* ^−*5*^	*10*	*0.012*	*0.058*
**Signal transduction** **(** **n** **=** **6** **)**						
Adipocytokine signaling^b^	14	0.0070	0.017	9	9.30 × 10^−4^	0.013
*Fc Epsilon RI Signaling* ^*a*,*b*^	*24*	*1.51* × *10* ^−*4*^	*6.73* × *10* ^−*4*^	*11*	*0.0018*	*0.021*
LPS-stimulated MAPK signaling^a^	23	2.75 × 10^−6^	4.65 × 10^−5^	9	0.0025	0.024
NF-κB signaling^a^	33	3.02 × 10^−5^	2.04 × 10^−4^	14	0.0016	0.021
p38 MAPK signaling^a^	20	0.0037	0.0082	9	0.014	0.062
SAPK/JNK signaling^a^	18	0.011	0.021	14	1.35 × 10^−5^	5.56 × 10^−4^
**Cardiovascular function** **(** **n** **=** **2** **)**						
Cardiac hypertrophy signaling^a^	42	2.45 × 10^−4^	9.22 × 10^−4^	16	0.013	0.062
Renin-angiotensin signaling^a^	27	3.55 × 10^−5^	2.24 × 10^−4^	14	8.91 × 10^−5^	0.0025
**Inflammation** **/** **Immune function** **(** **n** **=** **8** **)**						
B cell activating factor signaling^a^	10	0.013	0.023	5	0.022	0.090
CCR3 signaling in eosinophils^a^	24	0.0013	0.0035	13	5.25 × 10^−4^	0.0095
CCR5 signaling in macrophages^a^	13	0.030	0.048	11	5.62 × 10^−5^	0.0019
Chemokine signaling^a^	21	4.37 × 10^−5^	2.54 × 10^−4^	8	0.0096	0.049
CXCR4 signaling^a^	28	0.0065	0.014	11	0.045	0.14
Epithelial cell signaling in *Helicobacter pylori* infection^b^	16	0.0011	0.0048	8	0.0063	0.031
*Natural killer cell signaling* ^*a*,*b*^	*26*	*1.15* × *10* ^−*4*^	*5.54* × *10* ^−*4*^	*12*	*0.0013*	*0.019*
Role of PKR in Interferon Induction and Antiviral Response^a^	11	0.0051	0.011	5	0.025	0.094
**Other** ** (** **n** **=** **3** **)**						
Hepatic cholestasis^a^	24	0.014	0.026	12	0.0076	0.042
Macropinocytosis^a^	17	6.46 × 10^−4^	0.0021	8	0.0043	0.034
Xenobiotic metabolism signaling^a^	37	0.044	0.065	19	0.0053	0.036

^a^Ingenuity Pathways Knowledge Base Pathways with Number of Genes ≥ 3, P Values < 0.05, FDRs < 0.05 at either Week 8 or Week 12 and FDRs < 0.10 at both were selected by Ingenuity Pathway Analysis (IPA) URL: http://www.ingenuity.com/.

^b^Kyoto Encyclopedia of Genes and Genomes (KEGG) pathways with Number of Genes ≥ 3 and Gamma P Values < 0.05, and FDRs < 0.05 at either Week 8 or Week 12 and FDRs < 0.10 at both were selected by Onto-Tools URL: http://vortex.cs.wayne.edu/ontoexpress/. For pathways identified by both IPA and Onto-Tools (shown in italic), only IPA results were presented.

^c^FDR, denoting false discovery rate, was estimated by the Benjamini-Hochberg (BH) method.

#### Pathways identified by onto-tools pathway-express

Next, we performed pathway analysis on the nominally significantly expressed genes using Onto-Tools Pathway-Express. A total of 47, 21, 32, and 25 KEGG pathways with at least three overexpressed genes were enriched at nominal P values < 0.05 between responders and non-responders for the Week 8 TPM, Week 8 placebo, Week 12 TPM, and Week 12 placebo groups, respectively. Among them, eight significantly enriched KEGG pathways with FDRs < 0.05 at either time point or FDRs < 0.10 at both time points were shared exclusively by the Week 8 and Week 12 TPM groups (Table 3). Comparing the pathways detected by Onto-tools with those detected by IPA, we found three were shared: synaptic long-term potentiation, Fc epsilon RI signaling, and natural killer-cell signaling. In contrast, no significantly enriched KEGG pathways were shared by the Week 8 and Week 12 placebo groups, with FDRs < 0.10 at both time points. Again, although 81, 64, 60, and 65 pathways were detected for the Week 8 TPM, Week 8 placebo, Week 12 TPM, and Week 12 placebo groups, after Bonferroni correction, only 19, 5, 3, and 5 pathways remained significant. Furthermore, only two pathways (i.e., MAPK signaling and T-cell receptor signaling) were shared exclusively by the Week 8 and Week 12 TPM groups, and no pathways were shared by the Week 8 and Week 12 placebo groups.

Combining the results of IPA and Onto-Tools Pathway-Express, at the nominal P values < 0.05 and further restricting by FDRs < 0.05 at either time point or < 0.10 at both, a total of 27 pathways were identified (see Table 3). These pathways are involved in a spectrum of physiological functions: some are associated mainly with signal transduction (Fc epsilon RI signaling, LPS-stimulated MAPK signaling, p38 MAPK signaling, and SAPK/JNK signaling), whereas others are related to cardiovascular function (cardiac hypertrophy signaling, and renin-angiotensin signaling), and inflammation/immune function (B-cell activating-factor signaling, CCR3 signaling in eosinophils, CCR5 signaling in macrophages, chemokine signaling, CXCR4 signaling, epithelial cell signaling in *Helicobacter pylori* infection, natural killer cell signaling, and role of PKR in interferon induction and antiviral response).

**Figure 1:**
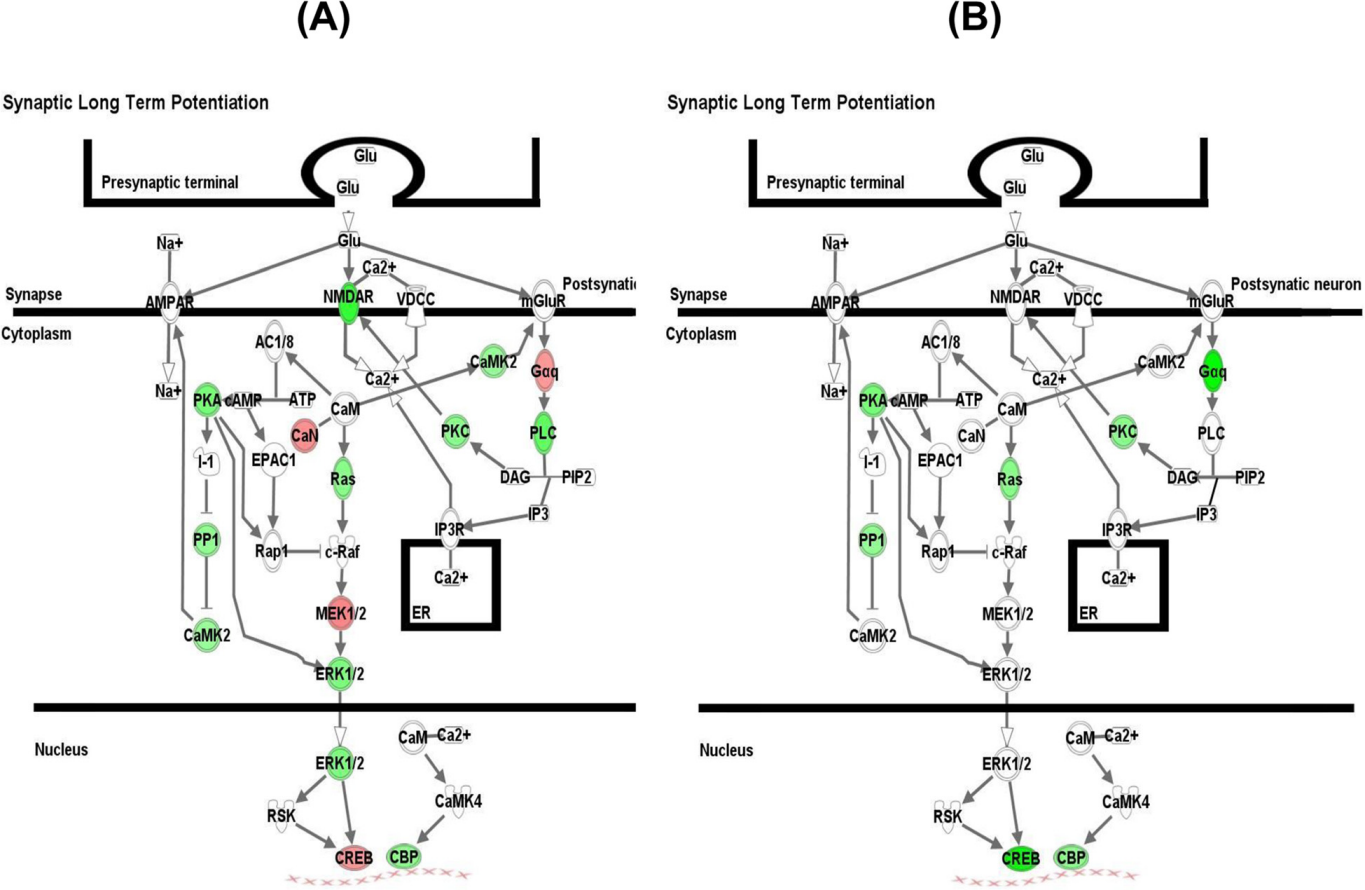
**Enriched synaptic long**-**term potentiation canonical pathway** **,** **identified by ingenuity pathway analysis based on differentially expressed genes** **(** **P value** **<** **0.05** **)** **with the ordinary student**’**s** ***t*** **-test.** The pathway was also detected by onto-tools pathway-express. **(A)** Week 8 TPM group (29 genes: *ATF2*, *CAMK2D*, *CAMK2G*, *CREB1*, *EP300*, *GNAQ*, *GRINA*, *MAP2K1*, *MAPK1*, *MAPK3*, *PLCB2*, *PPP1CA*, *PPP1CB*, *PPP1CC*, *PPP1R10*, *PPP1R12A*, *PPP1R14B*, *PPP1R7*, *PPP3CB*, *PPP3CC*, *PRKACA*, *PRKACB*, *PRKAR1A*, *PRKCD*, *PRKCH*, *PRKCI*, *PRKCQ*, *PRKCZ*, and *RRAS*); and **(B)** Week 12 TPM group (10 genes; *ATF4*, *CREB5*, *EP300*, *GNAQ*, *KRAS*, *PPP1R10*, *PRKACB*, *PRKAR2A*, *PRKCB*, and *PRKCQ*). Symbols with a single border represent single genes; those with a double border represent complexes of genes or the possibility that alternative genes might act in the pathway. Red symbols represent up-regulated gene clusters and green symbols represent down-regulated clusters.

The essential pathways related to neuronal function/synaptic plasticity include alpha-adrenergic signaling, ephrin receptor signaling, ErbB signaling, FGF signaling, GnRH signaling, mTOR signaling, neurotrophin/TRK signaling, and synaptic long-term potentiation. The genes in the synaptic long-term potentiation pathway that were changed by TPM at Week 8 and Week 12 are depicted in Figure 1.

## Discussion

**Figure 2:**
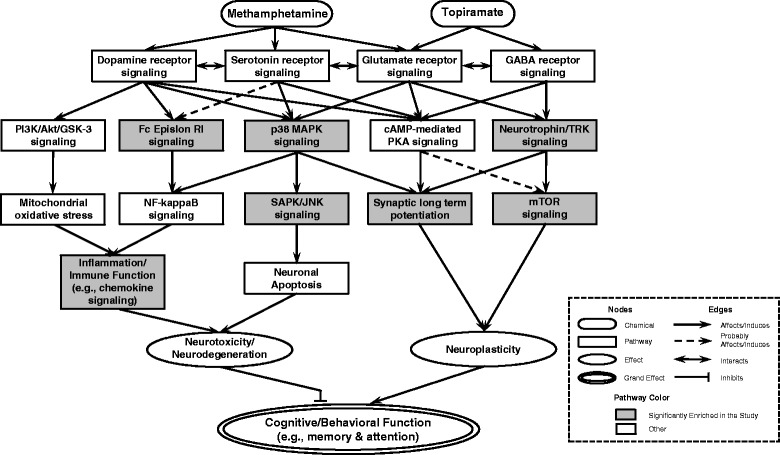
**Integrated model of the biological pathways related to TPM treatment for methamphetamine addiction.** The joint effects of TPM and methamphetamine act on multiple molecular pathways that eventually result in modulations of neuroplasticity and neurotoxicity/neurodegeneration, which have a combined effect on cognitive/behavioral function. Pathways enriched exclusively in the TPM responder groups at Weeks 8 and 12 are highlighted in gray.

The current study is the first genome-wide expression investigation into the effects of TPM for the treatment of METH dependence. By profiling genome-wide expression patterns in human white blood cells from METH-dependent subjects who received either oral TPM or placebo, we identified various number of genes that are differentially expressed between responders and non-responders in the TPM-treated and placebo control groups. Further clustering of these altered genes according to their function revealed the significantly enriched pathways governing neuroplasticity and neurotoxicity/neurodegeneration (see Figure 2). Given the primary purpose of this clinical trial, in this discussion, we focus primarily on how TPM may regulate molecular pathways of synaptic plasticity underlying METH’s reward and reinforcing effects that influence abstinence.

Exposure to drugs of abuse triggers various gene expression changes resulting in complex neural adaptations that determine the addictive properties of abusive drugs [27]. Among these changes, modifications in long-term synaptic potentiation (*LTP*) of neuroplasticity are fundamental in instilling reward and reinforcing the drug effects [28]. With evidence from decades of molecular research, it is established that METH alters *LTP* through activation of dopamine or glutamate surface receptors or both [29] that are linked to the intracellular signal transduction extracellular-signal-regulated-kinase (ERK) pathway. Among the surface neurotransmitter receptors regulating this pathway, the only receptor gene we found to be differentially expressed in the responders and non-responders to TPM was ionotropic glutamate receptor N-methyl D-aspartate-associated protein 1 (*GRINA*). This protein is a subtype of N-methyl D-aspartate (NMDA) receptors that are antagonized by TPM [30,31] and activated by METH [32,33]. In the TPM responder group, *GRINA* was down-regulated at Week 8, implying fewer NMDA receptors at the synapse. Although expression of none of the other primary target genes of TPM (such as GABA_A_ and AMPA/kainate glutamate receptors) was altered by TPM, three genes coding for membrane trafficking proteins (*DLG1*, *SNAP23*, and *TRAK2*) associated with these receptors were up-regulated in TPM responders compared with non-responders and placebo-treated subjects. The *DLG1* gene encodes a synaptic scaffolding protein (also known as synapse-associated protein 97) involved in synapse formation [34] and trafficking of AMPA [35,36], kainate [37], and NMDA [38,39] glutamate receptors. SNAP23 is a scaffolding protein that aids in stabilizing NMDA receptors at the neuronal surface [40]. The *TRAK2* product is involved in GABA_B_-receptor trafficking [41]. In rat neocortex, *DLG1* mRNA is up-regulated by the NMDA antagonist phencyclidine but not by METH [42]. Considering these factors, it is possible that TPM-associated alterations in the expression of AMPA, kainite, and GABA_A_ receptors at the neuronal surface are more likely to be further governed by post-transcriptional modifications such as receptor phosphorylation and trafficking to and from the synaptic membrane, rather than through alterations in their transcription.

Once METH activates its neural receptors, they activate ERK via the upstream cytoplasmic regulators of the ERK pathway; activated ERK translocates from the cytoplasm to the nucleus and phosphorylates cAMP response element binding protein (CREB) [27] to facilitate METH-induced gene expression, serving as the mediator between the nucleus and the target receptors of METH at the neuronal surface [43]. The drug might activate the ERK pathway via either up-regulation of gene transcription, post-transcriptional activation of protein phosphorylation, or both. In the present study, expression of several genes of the ERK pathway was down-regulated in responders at Week 8 of TPM treatment compared with non-responders and the placebo-treated subjects (see Figure 1A), suggesting a “reversal” of METH-induced up-regulation of ERK pathway genes. At Week 8, these TPM-related down-regulated genes included ERK-1 (*MAPK3*) and its upstream regulators, protein kinase A (*PRKACA*), protein kinases C and Z (*PRKCD* and *PRKCZ*), Ras-related genes (*ARHGEF2*, *RHOT2*, and *RRAS*), and *EP300*, which encodes a transcriptional co-activator that forms a complex with CREB-binding protein (CBP). By Week 12, besides *EP300*, the transcription factor CREB gene *CREB5* expression was down-regulated in TPM responders.

Given these findings, it is reasonable to hypothesize that (1) reversal of ERK and CREB over-expression results in blocking of METH-dependent transcription activity and consequently disruption of METH-induced *LTP* and (2) non-responders may harbor variants that affect expression of genes that are down-regulated in TPM responders. These possibilities have gained support from several lines of evidence reported by other investigators. For example, Narita et al. [29] demonstrated that blockade of protein kinase C (PKC) abolishes behavioral sensitization to METH. Human laboratory studies have indicated a partial inhibition of METH’s reinforcing effects by TPM at the same dosage used in the current study [23]. More importantly, our findings corroborate the concept that TPM would be a possible treatment for METH addiction through facilitating the inhibitory effects of GABA and blocking glutamate excitatory effects on dopamine neurons [22,23].

Apart from pathways governing neural plasticity, the functional category with the largest number of affected pathways was in immune function (see Table 3). Data on TPM’s effects on immune mediators is sparse, with a few studies emerging recently. Among the ten immune function-related pathways detected in the current study, only the T-cell receptor signaling pathway has been reported previously to be regulated by TPM [44]. A common feature of all these pathways and the pathways governing neuroplasticity is their use of the mitogen-activated-protein-kinase (MAPK) pathway as the central component. As this is not the primary focus of this report, a detailed discussion of those immune-related pathways will not be provided here.

The reliability of our findings is strengthened by a number of aspects of our study design: First, the present study included both a positive (TPM non-responders) and a negative (placebo) control group. Inclusion of a placebo group provided us with a reference necessary for the exploration of gene expression alterations induced specifically by TPM rather than by the absence or reduction of METH use or any other non-specific factors. For example, *EP300*, a member of the CREB gene family, was down-regulated in both the Weeks 8 and 12 TPM responder and the Week 12 placebo responder groups, suggesting that the regulatory effect of the gene is not specific to TPM, whereas *CREB5*, discussed above, was down-regulated only in Week 12 TPM responders, suggesting a TPM-specific effect. Further, the inclusion of a positive control group aided us in identifying genes and pathways associated with METH abstinence, which was the primary outcome of this clinical study. Second, we analyzed expression data from three time points, namely, the baseline (prior to starting TPM treatment) and Weeks 8 and 12 for each individual. This approach allowed us to correct for any confounding effects that might be caused by significant individual gene expression differences among subjects at baseline, by normalizing the extent of expression at Weeks 8 and 12 with the patient’s own baseline expression and increasing the reliability of the findings by utilizing Week 12 expression patterns to confirm those that occurred at Week 8. The third main strength of the present study is that the dose of TPM administered throughout the treatment period was well within the drug’s therapeutic range [22,23], and therefore, we can confidently conclude that the TPM-dependent expression alterations we detected were not related to TPM’s toxic effects, but rather to its therapeutic effects. Finally, we believe that, with the level of rigorousness of the clinical and statistical criteria employed in defining treatment responders and significantly altered genes and pathways, the chance that our findings are falsely positive is minimal.

However, this study is limited by several factors, of which the most notable is the small sample size for some comparison groups. Especially, the number of responders in the TPM and placebo groups were not balanced for either Week 8 or Week 12 (4 and 2 subjects for Weeks 8 and 12 in the placebo group vs. 5 and 6 subjects in TPM group for Weeks 8 and 12). However, these numbers are not distinctly smaller than those in other pharmacogenomic/expression studies published in the literature [45] and provided us with an 85% statistical power to draw conclusions about individual genes and pathways [46]. On the other hand, it could be argued that the imbalance in the number of responders in the two groups was attributable in part to weaker effects of the placebo in promoting abstinence compared with TPM. Because of the small samples, we did not consider covariate effects such as age, sex, and ethnicity in assessing single-gene effects. Although we believe the results obtained from such samples are reliable, extra attention should be paid in interpreting the expression pattern of single genes, especially those identified from the placebo groups. Another main limitation of our study is that we used a peripheral white blood cell model to study the gene expression alterations associated with neuronal functions. Peripheral blood is an easily accessible source of RNA for analysis of environmental exposure and disease conditions [47-49]. Circulating leukocytes can be used to infer gene expression in other tissues [50]. Indeed, constituents of blood maintain the balance of homeostasis, modulate immunity or inflammation, partake in stress signaling, and facilitate cellular communication in vascular-associated tissues, including those of the CNS [51]. Sullivan et al. [52] conducted a secondary data analysis of transcriptional profiling of 79 diverse human tissues and found that whole blood shared substantial gene expression similarities with multiple brain tissues such as the amygdala, caudate nucleus, prefrontal cortex, and whole brain (the median Spearman correlation coefficient for the group was 0.52), indicating that gene expression in whole blood can be a robust and valid surrogate for gene expression in the brain [52]. However, in another recent study, only weak correlation was detected between gene expression in the brain and that in blood samples [53]. Under such conditions, although the gene expression data from whole blood may provide useful information to infer the biological processes underlying the interaction of TPM and METH in the neuronal system, more direct evidence obtained from brain tissues is necessary in order to verify the findings reported in this study.

## Conclusions

In summary, with application of rigorous clinical and statistical criteria, we demonstrated that TPM mitigates METH’s reinforcing effects, possibly through reversal of some of the dysregulated genes in pathways governing synaptic plasticity to their normal state. Further studies are necessary to replicate these findings as well as to identify genetic variations that may have resulted in regulatory differences observed in TPM responders vs. non-responders. Identification of such molecular mechanisms will help greatly in developing efficacious medications for the treatment of METH dependence.

## Methods

### Study design and blood sample collection

This was a double-blind, multi-center, placebo-controlled, randomized, parallel-group study for METH-dependent outpatients [26]. Under the inter-agency agreement between the National Institute on Drug Abuse and the Veterans Affairs (VA) Cooperative Programs, eight medical centers participated. The sites’ Institutional Review Boards and the VA Human Rights Committee approved the protocol for and conduct of the study.

Subjects meeting the eligibility criteria after a 14-day screening period and a baseline assessment were randomized into equivalent-size groups for oral treatment with TPM or placebo daily for 91 days. There was a dose titration phase (Days 1 to 35) to a maximum tolerated dose of TPM not to exceed 200 mg/day, a maintenance phase (Days 36 to 84), and a taper phase (Days 85 to 91). To continue in the study, subjects had to maintain a minimum daily dose of 50 mg. Blood samples were collected on Day 1 (considered the baseline) and at the end of Weeks 8 and 12 from every participant who consented to participate in the genetics/expression study. The rationale for using weeks 8 and 12 of TPM treatment in the genetic/expression study was that these two time points were in the middle of the maintenance phase of the maximum dose for each patient and the end of treatment, respectively. At the two time points, because the TPM dose given to each patient became relatively stable, this would reduce variability of drugs received among patients, thus likely increasing statistical power of identifying differentially expressed genes and pathways. All blood samples for this study were collected in PAXgeneTM blood tubes using standard phlebotomy technique.

### Primary efficacy outcome measure

The primary efficacy outcome measure was METH use or non-use during each week of the entire period from weeks 1 to 12. For each participant, urine samples were collected three times per week. A *positive use week* was defined as any week in which at least one of the urine tests was positive for METH and a *negative use week* as one in which all three tests were negative. The value was considered to be missing if no urine sample was collected. On the basis of the primary efficacy outcome measures for the entire trial period, each study participant in either the TPM or the placebo group was classified as either a positive or negative responder to the treatment, which was referred to as responder or non-responder in this study. For example, a TPM responder at Week 8 means for this participant receiving TPM treatment no METH was detected in all the three urine samples for Week 8 (negative use week); whereas for a TPM non-responder, METH was detected in one or more urine samples. Our aim was to determine which genes were differentially expressed in the responders and non-responders of the TPM or placebo group during a given week. Because we collected blood samples from each participant at baseline and Weeks 8 and 12, we formed four analysis groups: Week 8 TPM, Week 8 placebo, Week 12 TPM, and Week 12 placebo, according to the positive or negative use information at Weeks 8 and 12, respectively. Because not all participants contributed blood samples at both time points, the final sample sizes were different for each group.

### RNA isolation and gene expression analysis

Blood samples were collected at approximately the same time of day for each participant for all three time points to control for potential circadian rhythm effects on gene expression. Total RNA was extracted using the PAXgene™ Blood RNA Isolation Kit (Qiagen, Valencia, CA, USA). Genome-wide expression of each sample was assessed with a Human Genome U-133 Plus 2.0 array (Affymetrix Inc., Santa Clara, CA, USA) by Expression Analysis Inc. (Durham, NC). Briefly, the double-stranded cDNA was used in a T7 RNA polymerase *in vitro* transcription reaction (Ambion, Austin, TX, USA) containing biotin-labeled ribonucleotides CTP and UTP. The resulting labeled cRNAs were then hybridized to HG-U133plus2.0 arrays.

#### Quality control and bioinformatics analysis of array data

Outlier array detection and quality assessment: In total, there were 212 HG-U133plus2.0 arrays from 99 study participants, which included 91 arrays at baseline, 65 at Week 8, and 56 at Week 12. The 212 “.CEL” files generated by the Microarray Suite (MAS 5.0; Affymetrix) were converted into “.DCP” files using dChip 2008 software (http://biosun1.harvard.edu/~cli/dchip_2008_05.exe). We used the “% array outlier” diagnostic metric to detect outlier arrays, defined as the percentage of outlier probe sets in one array [54]. If this percentage exceeded 5%, the array was called an “outlier.” Three arrays at baseline were found to have a “% array outlier” metric > 5% and were excluded from further analysis. For quality assessment of the remaining 209 chips, the distributions of log_2_-transformed raw probe-level intensities were visualized by boxplots, and no anomalies were found (data not shown).

Data pre-processing and normalization: Data quality assessment was followed by data pre-processing and normalization with the Robust Multi-Array Average (RMA) algorithm [55], implemented in the RMA function in the Bioconductor Affy package [56]. The RMA is a statistical method comprising three procedures performing the following functions: (i) convolution background correction; (ii) probe-level quantile normalization; and (iii) median polish summarization for each probe set to estimate the log_2_ scale expression values. A matrix of expression values was computed for the 209 “.CEL” files. The expression values after normalization were similar across arrays.

**Figure 3:**
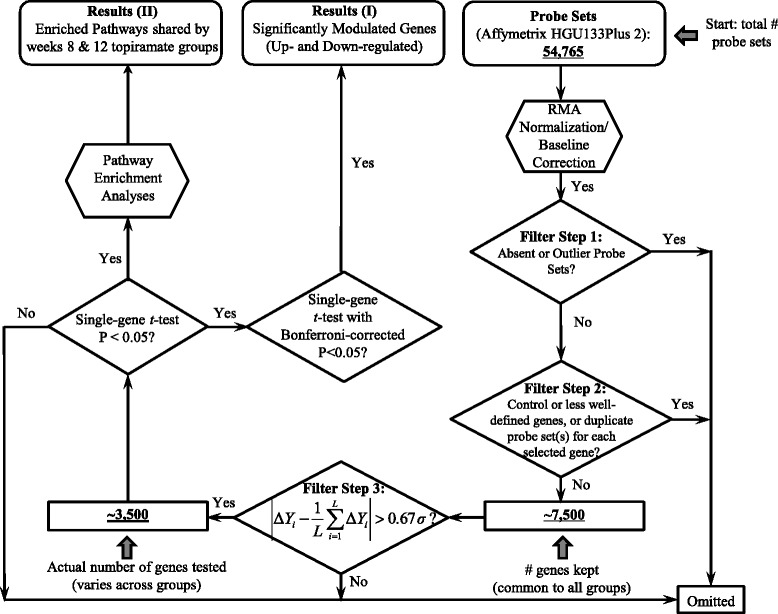
**Schematic diagram of study workflow** **,** **including probe set filtering steps and statistical test strategies for detecting significant single genes and pathways.** The probe intensities measured in 209 hybridized Affymetrix HG-U133 plus 2.0 arrays were normalized by Robust Multichip Average followed by a baseline correction step. Probes marked ‘Presence’ in fewer than four arrays in each group (because for Week 12 placebo group, only two positive responders were included, probes with two valid measurements were kept) were removed. Probes corresponding to control or less well-defined genes, and duplicated probes were removed. Genes with low FCs; i.e., within 1 standard deviation (denoted by σ) for a total of *L* (~7500) genes also were removed, as most of them were not likely to be differentially expressed to a statistically significant extent. The remaining genes were tested by the ordinary Student’s *t*-test, and genes with P values < 0.05 were used for pathway analysis. In total, 3698, 3532, 3328, and 3405 genes were tested for the Week 8 TPM, Week 8 placebo, Week 12 TPM, and Week 12 placebo groups, respectively.

Probe set filtering: The HG-U133plus2.0 array contains 54,675 oligonucleotide-based probe sets. However, not all of these sets correspond to well-defined genes. By using the latest Affymetrix annotation file (dated November 30, 2008), we found that a total of 33,752 (61.73%) probe sets correspond to unique genes, whereas the remaining probe sets do not and were thus excluded from our statistical analysis. Furthermore, we implemented a series of filtering procedures to reduce the number of probe sets to be tested, which is summarized as follows: (*i*) *Filtering* “*Absence call*” *probe sets*: We applied a Bioconductor package called “Presence-Absence Calls with Negative Probesets” (PANP) that uses Affymetrix-reported probe sets with no known hybridization partners. PANP uses a simple empirically derived approach to generate P values for thresholds to define “presence/absence” calls. The “presence/absence” calls and P values are returned as two matrices: “Pcalls” and “Pvals,” respectively. Probe sets with < 50% present calls among all arrays within each group were removed, which is considered restrictive [57,58], leaving ~15,000 probe sets for further analysis. (*ii*) *Filtering biologically irrelevant genes and duplicate probe set*(*s*) *for each selected gene*: Among the ~15,000 probe sets, control sets of various housekeeping genes (e.g., *GAPDH*) and spiked-in controls (e.g., *Ec*-*bioB*, *Ec*-*bioC*, *Ec*-*bioD*), as well as those genes that are not well defined or have unknown functions were removed. After removing duplicate probe set(s) for the same gene, such that only the probe set with the smallest test statistic was kept for each gene [59], about 7,500 genes remained. (*iii*) *Filtering out genes with low fold changes* (*FCs*): Genes with log_2_(FC) < 0.67 × standard deviation (SD) away from the group mean (i.e., between the first and the third quartile assuming that log_2_(FC) follows a normal distribution) were removed. After these sequential steps of filtering, about 3,500 genes were left for downstream statistical analyses for each group. A schematic diagram of the detailed data mining and analysis plan is shown in Figure 3.

#### Statistical analysis to identify differentially expressed genes and pathways

After data quality checking, pre-processing, normalization, and probe set filtering, we analyzed the microarray data at both the single-gene level (where one seeks to determine whether each gene is expressed differently under different conditions) and the pathway level (where one intends to determine if a biological pathway shows a different expression pattern under different conditions). Considering the individual variations at the baseline, we normalized each individual’s Week 8 and Week 12 expression values by the corresponding baseline values prior to the identification of differentially expressed genes and biological pathways.

##### Single-gene analysis

The primary goal of this step is to detect those genes with significantly different expressions in two comparison groups that cannot be ascribed to chance or natural variability [60]. The ordinary Student’s *t*-test, implemented by MATLAB (MathWorks, Natick, MA), was employed for testing differential expressions in a gene-by-gene manner. To correct for multiple testing, both Bonferroni correction and false discovery rate (FDR); i.e., the expected proportion of falsely rejected null hypotheses among the rejected hypotheses, which was estimated by the Benjamini-Hochberg (BH) procedure [61], were applied.

##### Pathway analysis

Because gene expression is a well-coordinated system, expressions of different genes generally are not independent. Pathway analysis can reduce the number of hypotheses to a more manageable number that directly addresses questions of biological interest. During the past few years, various bioinformatics tools have been developed for pathway analysis, although none has gained widespread acceptance [60]. Therefore, in the current study, significantly enriched pathways of differentially expressed genes were detected using the following bioinformatics tools:

##### Ingenuity Pathway Analysis (IPA) (http://www.ingenuity.com/)

The IPA is a web-based bioinformatics tool [62]. A given set of input genes was associated with molecular networks based on their connectivities in the Ingenuity Pathways Knowledge Base. Fisher’s exact test was used to determine the probability that each biological function assigned to that data set was attributable to chance alone [63].

##### Onto*-*Tools Pathway*-*Express (http://vortex.cs.wayne.edu/projects.htm)

The Onto-Tools Pathway-Express [64,65] implements an innovative “Impact Factor Analysis” based on the Kyoto Encyclopedia of Genes and Genomes (KEGG) pathway database. Distinct from either “Over-Representation Analysis” (ORA) or “Gene Set Enrichment Analysis” (GSEA), Onto-Tools Pathway-Express uses a systems biology approach to identify pathways that are significantly impacted in any condition monitored by high-throughput gene expression technology. This new “Impact Factor Analysis” not only incorporates the classical probabilistic component but also includes important biological factors that are not captured by the existing techniques; e.g., the magnitude of the expression changes of each gene, the position of the differentially expressed genes on given pathways, the topology of the pathway that describes how genes interact, and the type of signaling interactions between them [64]. Based on a given set of input genes, for each pathway detected, a perturbation factor gamma P value and a corresponding FDR were calculated, taking into consideration the normalized FC of the gene and the number of genes upstream of its position in the pathway. Because IPA and Onto-Tools Pathway-Express have applied distinct statistical algorithms based on independent knowledge databases, these two bioinformatics tools are complementary, and thus their results are combined.

## Additional files

Additional file 1: Figure S1. Diagram of sample selection and grouping of responders and non-responders to TPM treatment and Placebo for Weeks 8 and 12. Additional file 2: Tables S1-4. A detailed list of genes and pathways identified from the study at different treatment times.
