# Supplementary material for: Transcriptome profiling and pathway analysis of genes expressed differentially in participants with or without a positive response to topiramate treatment for methamphetamine addiction
Source: BMC Med Genomics. 2014 Dec 12;7:65. doi: 10.1186/s12920-014-0065-x (PMC4279796; doi:10.1186/s12920-014-0065-x)

Supplementary Figure 1A: Diagram illustration for sample selection and grouping of responders and non-responders to Topiramate treatment and Placebo for Week 8

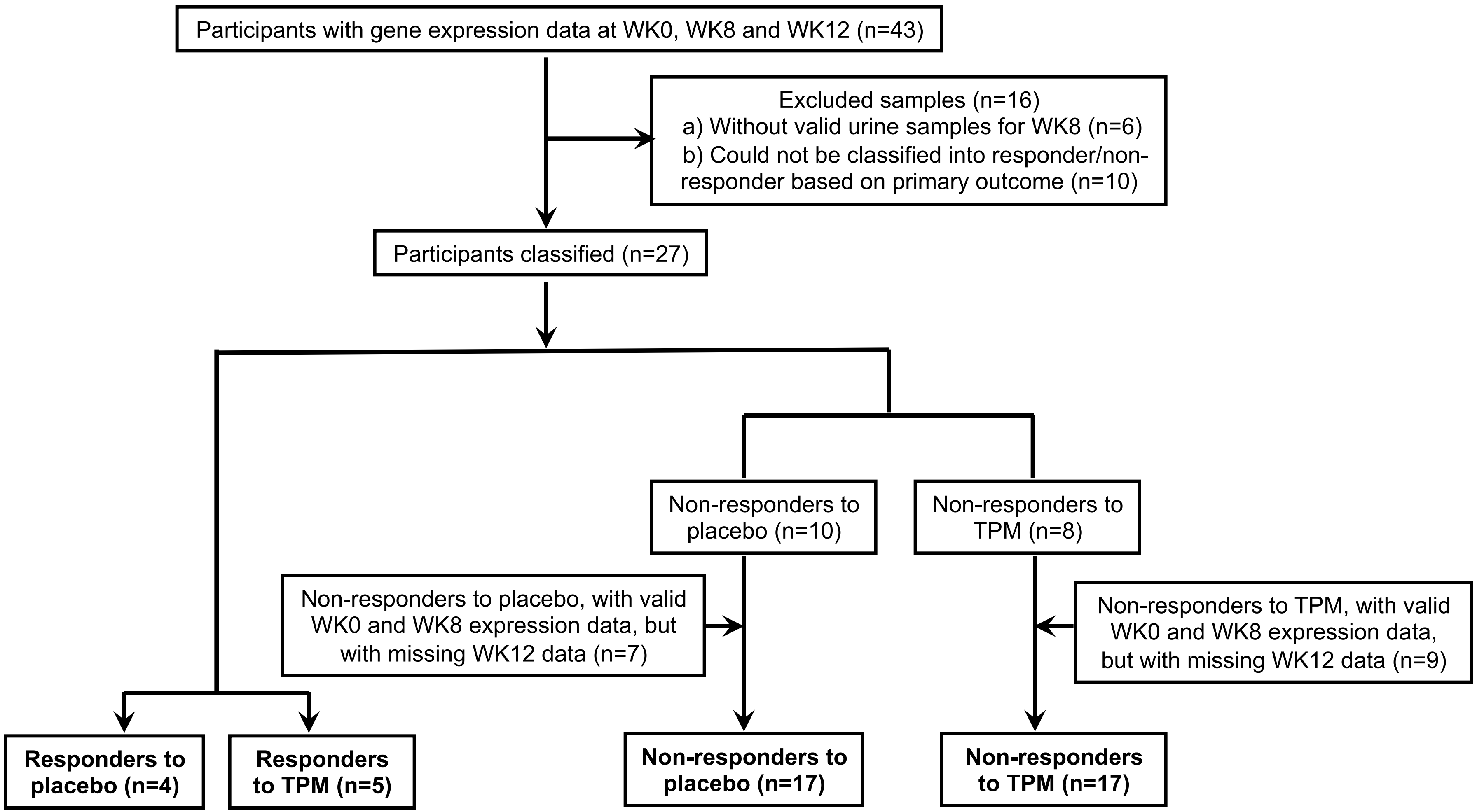

Supplementary Figure 1B: Diagram illustration for sample selection and grouping of responders and non-responders to Topiramate treatment and Placebo for Week 12

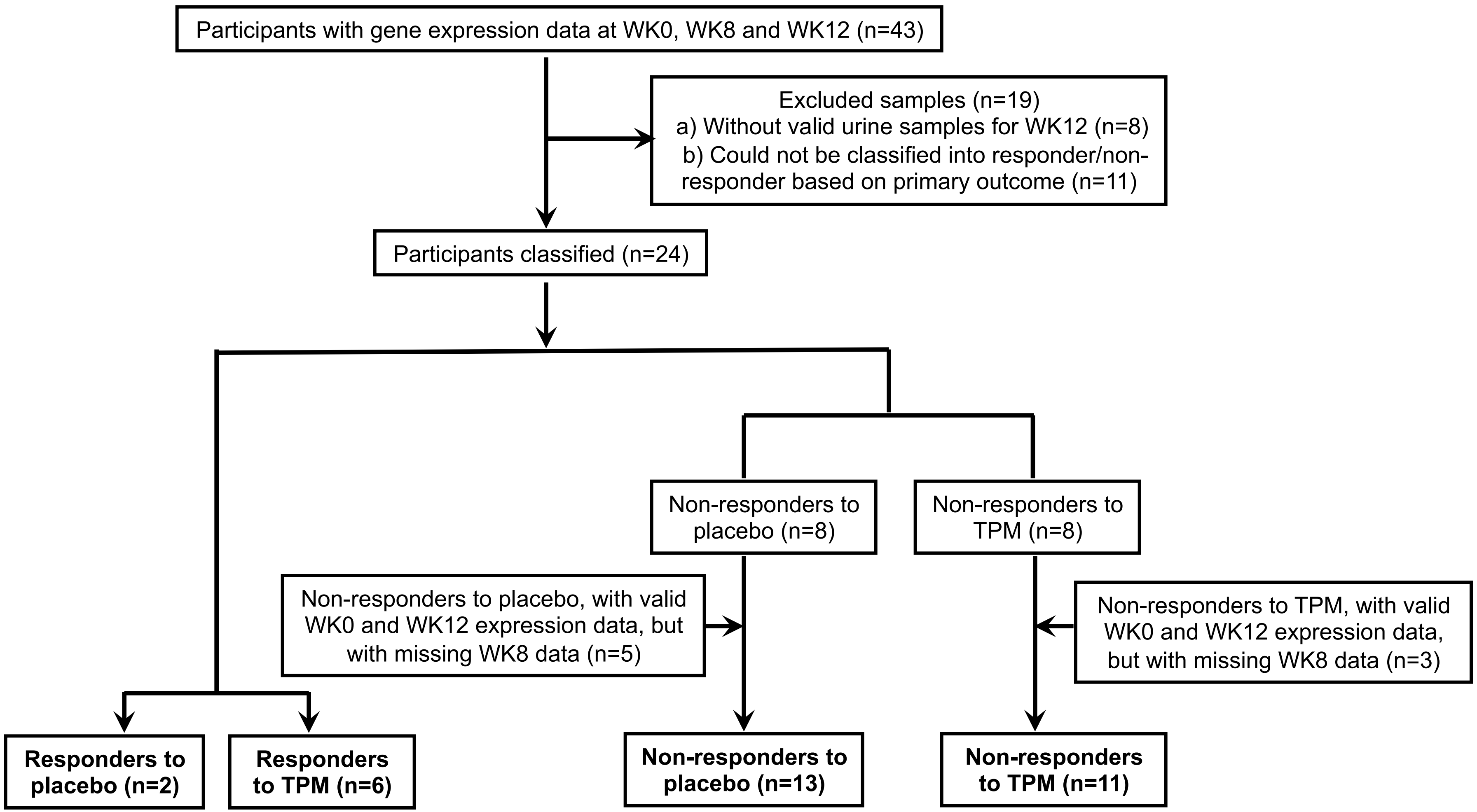

Supplement: Additional file 1: Figure S1. — Diagram of sample selection and grouping of responders and non-responders to TPM treatment and Placebo for Weeks 8 and 12. [file 12920_2014_65_MOESM1_ESM.pdf]
