# Supplementary material for: Transcriptome profiling and pathway analysis of genes expressed differentially in participants with or without a positive response to topiramate treatment for methamphetamine addiction
Source: BMC Med Genomics. 2014 Dec 12;7:65. doi: 10.1186/s12920-014-0065-x (PMC4279796; doi:10.1186/s12920-014-0065-x)
Supplement: Additional file 2: Tables S1-4. — A detailed list of genes and pathways identified from the study at different treatment times. [file 12920_2014_65_MOESM2_ESM.doc]

#### Table S1. Differentially Expressed Gene List for Week 8 Topiramate Group Between Responders (*n* = 5) and Non-responders (*n* = 17) Responders^a^

|  |  |  |  |  |  |  |
| --- | --- | --- | --- | --- | --- | --- |
| Gene Symbol | GO Annotation^b^ | GenBank  Acc. Number | Gene Name | FC^c^ | P Value^d^ | FDR^e^ |
|  |  |  |  |
|  |  |  |  |  |  |  |
|  |  |  |  |  |  |  |
| GDI2 | Rab GDP-dissociation inhibitor activity | NM_001494 | GDP dissociation inhibitor 2 | 1.63 | <1.00×10^-6^ | <1.00×10^-5^ |
| YME1L1 | nucleotide binding | AF070656 | YME1-like 1 (S. cerevisiae) | 1.69 | <1.00×10^-6^ | <1.00×10^-5^ |
| TRAK2 | cytoplasm | NM_015049 | trafficking protein, kinesin binding 2 | 1.44 | <1.00×10^-6^ | <1.00×10^-5^ |
| USF2 | transcription factor activity | NM_003367 | upstream transcription factor 2, c-fos interacting | 0.48 | <1.00×10^-6^ | <1.00×10^-5^ |
| PSMD12 | protein binding | AI446530 | proteasome (prosome, macropain) 26S subunit, non-ATPase, 12 | 1.77 | <1.00×10^-6^ | <1.00×10^-5^ |
| RXRA | transcription factor activity | BE675800 | retinoid X receptor, alpha | 0.36 | <1.00×10^-6^ | <1.00×10^-5^ |
| SSFA2 | actin binding | NM_006751 | sperm specific antigen 2 | 2.32 | <1.00×10^-6^ | <1.00×10^-5^ |
| PLCB2 | phosphoinositide phospholipase C activity | NM_004573 | phospholipase C, beta 2 | 0.52 | <1.00×10^-6^ | <1.00×10^-5^ |
| PIGB | mannosyltransferase activity | NM_004855 | phosphatidylinositol glycan anchor biosynthesis, class B | 1.56 | <1.00×10^-6^ | <1.00×10^-5^ |
| SMURF2 | ubiquitin ligase complex | AY014180 | SMAD specific E3 ubiquitin protein ligase 2 | 1.64 | <1.00×10^-6^ | <1.00×10^-5^ |
| RBPJ | recombinase activity | NM_015874 | recombination signal binding protein for immunoglobulin kappa J region | 1.77 | <1.00×10^-6^ | <1.00×10^-5^ |
| XPO1 | protein import into nucleus, docking | D89729 | exportin 1 (CRM1 homolog, yeast) | 1.98 | <1.00×10^-6^ | <1.00×10^-5^ |
| ELOVL5 | endoplasmic reticulum | AL136939 | ELOVL family member 5, elongation of long chain fatty acids (FEN1/Elo2, SUR4/Elo3-like, yeast) | 1.87 | <1.00×10^-6^ | <1.00×10^-5^ |
| TAPBP | Golgi membrane | AF029750 | TAP binding protein (tapasin) | 0.62 | <1.00×10^-6^ | <1.00×10^-5^ |
| RNF11 | ubiquitin ligase complex | AB024703 | ring finger protein 11 | 3.29 | <1.00×10^-6^ | <1.00×10^-5^ |
| FBXL5 | ubiquitin ligase complex | AF157323 | F-box and leucine-rich repeat protein 5 | 1.69 | <1.00×10^-6^ | <1.00×10^-5^ |
| DPM2 | glycosylphosphatidylinositol-N-acetylglucosaminyltransferase (GPI-GnT) complex | AF061729 | dolichyl-phosphate mannosyltransferase polypeptide 2, regulatory subunit | 0.39 | <1.00×10^-6^ | <1.00×10^-5^ |
| PTEN | regulation of cyclin-dependent protein kinase activity | BC005821 | phosphatase and tensin homolog | 2.01 | <1.00×10^-6^ | <1.00×10^-5^ |
| GANAB | protein binding | W87689 | glucosidase, alpha; neutral AB | 0.62 | <1.00×10^-6^ | <1.00×10^-5^ |
| GRINA | receptor activity | AL571424 | glutamate receptor, ionotropic, N-methyl D-aspartate-associated protein 1 (glutamate binding) | 0.40 | <1.00×10^-6^ | <1.00×10^-5^ |
| MAPK1IP1L | --- | AI671747 | mitogen-activated protein kinase 1 interacting protein 1-like | 1.39 | <1.00×10^-6^ | <1.00×10^-5^ |
| SOS2 | DNA binding | AI628605 | son of sevenless homolog 2 (Drosophila) | 2.51 | <1.00×10^-6^ | <1.00×10^-5^ |
| TLK2 | nucleotide binding | AU151689 | tousled-like kinase 2 | 1.40 | <1.00×10^-6^ | <1.00×10^-5^ |
| JMJD3 | binding | AA521267 | jumonji domain containing 3, histone lysine demethylase | 0.49 | <1.00×10^-6^ | <1.00×10^-5^ |
| BCR | protein serine/threonine kinase activity | U07000 | breakpoint cluster region | 0.70 | <1.00×10^-6^ | <1.00×10^-5^ |
| HLA-E | antigen processing and presentation of peptide antigen via MHC class I | M31183 | major histocompatibility complex, class I, E | 0.80 | <1.00×10^-6^ | <1.00×10^-5^ |
| SERBP1 | RNA binding | AF131807 | SERPINE1 mRNA binding protein 1 | 2.03 | <1.00×10^-6^ | <1.00×10^-5^ |
| ERBB2IP | ErbB-2 class receptor binding | NM_018695 | erbb2 interacting protein | 2.28 | <1.00×10^-6^ | <1.00×10^-5^ |
| U2AF2 | nucleotide binding | NM_007279 | U2 small nuclear RNA auxiliary factor 2 | 0.48 | <1.00×10^-6^ | <1.00×10^-5^ |
| NPEPL1 | aminopeptidase activity | AL139349 | aminopeptidase-like 1 | 0.80 | <1.00×10^-6^ | <1.00×10^-5^ |
| RPAP3 | binding | NM_024604 | RNA polymerase II associated protein 3 | 2.00 | <1.00×10^-6^ | <1.00×10^-5^ |
| ADAT1 | RNA binding | NM_012091 | adenosine deaminase, tRNA-specific 1 | 0.62 | <1.00×10^-6^ | <1.00×10^-5^ |
| NSFL1C | membrane fraction | NM_018839 | NSFL1 (p97) cofactor (p47) | 0.66 | <1.00×10^-6^ | <1.00×10^-5^ |
| SLC38A2 | plasma membrane | NM_018976 | solute carrier family 38, member 2 | 2.32 | <1.00×10^-6^ | <1.00×10^-5^ |
| ZFAND6 | molecular_function | AL136598 | zinc finger, AN1-type domain 6 | 2.25 | <1.00×10^-6^ | <1.00×10^-5^ |
| MRPL37 | structural constituent of ribosome | AF325707 | mitochondrial ribosomal protein L37 | 0.77 | <1.00×10^-6^ | <1.00×10^-5^ |
| NUDT5 | magnesium ion binding | BC000025 | nudix (nucleoside diphosphate linked moiety X)-type motif 5 | 1.34 | <1.00×10^-6^ | <1.00×10^-5^ |
| ATAD3B | nucleotide binding | BC002542 | ATPase family, AAA domain containing 3B | 0.68 | <1.00×10^-6^ | <1.00×10^-5^ |
| EMILIN2 | protein binding | AF270513 | elastin microfibril interfacer 2 | 0.71 | <1.00×10^-6^ | <1.00×10^-5^ |
| POLR1D | DNA binding | N32181 | polymerase (RNA) I polypeptide D, 16kDa | 1.51 | <1.00×10^-6^ | <1.00×10^-5^ |
| TMPO | chromatin | AL566034 | thymopoietin | 2.31 | <1.00×10^-6^ | <1.00×10^-5^ |
| MLLT1 | DNA binding | AW962605 | myeloid/lymphoid or mixed-lineage leukemia (trithorax homolog, Drosophila); translocated to, 1 | 0.68 | <1.00×10^-6^ | <1.00×10^-5^ |
| KPNA4 | protein binding | AI935246 | karyopherin alpha 4 (importin alpha 3) | 2.09 | <1.00×10^-6^ | <1.00×10^-5^ |
| SEC62 | --- | AI763287 | SEC62 homolog (S. cerevisiae) | 1.69 | <1.00×10^-6^ | <1.00×10^-5^ |
| NDUFV3 | NADH dehydrogenase activity | NM_021075 | NADH dehydrogenase (ubiquinone) flavoprotein 3, 10kDa | 0.61 | <1.00×10^-6^ | <1.00×10^-5^ |
| NFIC | transcription factor activity | AW134798 | Nuclear factor I/C (CCAAT-binding transcription factor) | 0.57 | <1.00×10^-6^ | <1.00×10^-5^ |
| ZNF281 | negative regulation of transcription from RNA polymerase II promoter | AA121673 | Zinc finger protein 281 | 2.59 | <1.00×10^-6^ | <1.00×10^-5^ |
| DGCR14 | molecular_function | L77566 | 3-phosphoinositide dependent protein kinase-1 | 0.69 | <1.00×10^-6^ | <1.00×10^-5^ |
| CLCN7 | voltage-gated ion channel activity | Z67743 | chloride channel 7 | 0.61 | <1.00×10^-6^ | <1.00×10^-5^ |
| SRCAP | nucleotide binding | AB002307 | Snf2-related CREBBP activator protein | 0.74 | <1.00×10^-6^ | <1.00×10^-5^ |
| SBF1 | inositol or phosphatidylinositol phosphatase activity | U93181 | SET binding factor 1 | 0.61 | <1.00×10^-6^ | <1.00×10^-5^ |
| RHOT2 | nucleotide binding | AI186666 | ras homolog gene family, member T2 | 0.68 | <1.00×10^-6^ | <1.00×10^-5^ |
| DNAJA1 | protein folding | AL534104 | DnaJ (Hsp40) homolog, subfamily A, member 1 | 1.66 | 1.00×10^-6^ | 5.066×10^-5^ |
| TXNL1 | cytoplasm | NM_004786 | thioredoxin-like 1 | 1.49 | 1.00×10^-6^ | 5.066×10^-5^ |
| ZFR | DNA binding | NM_016107 | zinc finger RNA binding protein | 2.14 | 1.00×10^-6^ | 5.066×10^-5^ |
| ZMYND11 | negative regulation of transcription from RNA polymerase II promoter | BE250417 | zinc finger, MYND domain containing 11 | 2.05 | 1.00×10^-6^ | 5.066×10^-5^ |
| TLK1 | nucleotide binding | NM_012290 | tousled-like kinase 1 | 2.27 | 1.00×10^-6^ | 5.066×10^-5^ |
| UBE2M | ubiquitin-protein ligase activity | NM_003969 | ubiquitin-conjugating enzyme E2M (UBC12 homolog, yeast) | 0.59 | 1.00×10^-6^ | 5.066×10^-5^ |
| OAS1 | RNA binding | NM_002534 | 2',5'-oligoadenylate synthetase 1, 40/46kDa | 0.54 | 1.00×10^-6^ | 5.066×10^-5^ |
| CD164 | extracellular region | BF669455 | CD164 molecule, sialomucin | 2.67 | 1.00×10^-6^ | 5.066×10^-5^ |
| NAPA | protein binding | BC001165 | N-ethylmaleimide-sensitive factor attachment protein, alpha | 0.49 | 1.00×10^-6^ | 5.066×10^-5^ |
| CDC42EP2 | opioid peptide activity | BC005406 | CDC42 effector protein (Rho GTPase binding) 2 | 0.50 | 1.00×10^-6^ | 5.066×10^-5^ |
| B4GALT3 | beta-N-acetylglucosaminylglycopeptide beta-1,4-galactosyltransferase activity | AF038661 | UDP-Gal:betaGlcNAc beta 1,4- galactosyltransferase, polypeptide 3 | 0.79 | 1.00×10^-6^ | 5.066×10^-5^ |
| EFR3A | binding | AW470003 | EFR3 homolog A (S. cerevisiae) | 1.89 | 1.00×10^-6^ | 5.066×10^-5^ |
| SUV39H1 | chromosome, telomeric region | NM_003173 | suppressor of variegation 3-9 homolog 1 (Drosophila) | 0.66 | 1.00×10^-6^ | 5.066×10^-5^ |
| CCDC59 | protein binding | NM_014167 | coiled-coil domain containing 59 | 1.86 | 1.00×10^-6^ | 5.066×10^-5^ |
| NSUN3 | methyltransferase activity | NM_022072 | NOL1/NOP2/Sun domain family, member 3 | 1.36 | 1.00×10^-6^ | 5.066×10^-5^ |
| CISH | regulation of cell growth | NM_013324 | cytokine inducible SH2-containing protein | 0.75 | 1.00×10^-6^ | 5.066×10^-5^ |
| CIRBP | nucleotide binding | AL565767 | cold inducible RNA binding protein | 0.57 | 1.00×10^-6^ | 5.066×10^-5^ |
| SLC43A2 | transport | AI742770 | solute carrier family 43, member 2 | 0.66 | 1.00×10^-6^ | 5.066×10^-5^ |
| JAK3 | nucleotide binding | BF512748 | Janus kinase 3 (a protein tyrosine kinase, leukocyte) | 0.59 | 1.00×10^-6^ | 5.066×10^-5^ |
| TOB1 | transcription corepressor activity | BF240286 | transducer of ERBB2, 1 | 2.28 | 1.00×10^-6^ | 5.066×10^-5^ |
| SLU7 | alternative nuclear mRNA splicing, via spliceosome | NM_006425 | SLU7 splicing factor homolog (S. cerevisiae) | 1.61 | 1.00×10^-6^ | 5.066×10^-5^ |
| PCNA | cyclin-dependent protein kinase holoenzyme complex | NM_002592 | proliferating cell nuclear antigen | 1.68 | 2.00×10^-6^ | 8.22×10^-5^ |
| DLG1 | endothelial cell proliferation | BG251175 | discs, large homolog 1 (Drosophila) | 1.66 | 2.00×10^-6^ | 8.22×10^-5^ |
| USP4 | cysteine-type endopeptidase activity | AI346043 | ubiquitin specific peptidase 4 (proto-oncogene) | 0.69 | 2.00×10^-6^ | 8.22×10^-5^ |
| ARHGEF2 | Rho guanyl-nucleotide exchange factor activity | NM_004723 | rho/rac guanine nucleotide exchange factor (GEF) 2 | 0.62 | 2.00×10^-6^ | 8.22×10^-5^ |
| SNAP23 | protein binding | BC003686 | synaptosomal-associated protein, 23kDa | 1.95 | 2.00×10^-6^ | 8.22×10^-5^ |
| PRKCI | polarisome | L18964 | protein kinase C, iota | 1.80 | 2.00×10^-6^ | 8.22×10^-5^ |
| P2RX1 | purinergic nucleotide receptor activity | U45448 | purinergic receptor P2X, ligand-gated ion channel, 1 | 0.58 | 2.00×10^-6^ | 8.22×10^-5^ |
| PRPF4B | nucleotide binding | Z25435 | PRP4 pre-mRNA processing factor 4 homolog B (yeast) | 1.97 | 2.00×10^-6^ | 8.22×10^-5^ |
| UBE2D1 | protein polyubiquitination | BC005980 | ubiquitin-conjugating enzyme E2D 1 (UBC4/5 homolog, yeast) | 2.41 | 2.00×10^-6^ | 8.22×10^-5^ |
| PSKH1 | nucleotide binding | AJ272212 | protein serine kinase H1 | 0.64 | 2.00×10^-6^ | 8.22×10^-5^ |
| TUBB2C | nucleotide binding | AA515698 | tubulin, beta 2C | 0.72 | 2.00×10^-6^ | 8.22×10^-5^ |
| PLEKHF2 | zinc ion binding | NM_024613 | pleckstrin homology domain containing, family F (with FYVE domain) member 2 | 2.33 | 2.00×10^-6^ | 8.22×10^-5^ |
| MSRB2 | protein-methionine-R-oxide reductase activity | NM_012228 | methionine sulfoxide reductase B2 | 0.59 | 2.00×10^-6^ | 8.22×10^-5^ |
| UBXN6 | --- | NM_025241 | UBX domain protein 6 | 0.42 | 2.00×10^-6^ | 8.22×10^-5^ |
| ATG7 | catalytic activity | AW270638 | ATG7 autophagy related 7 homolog (S. cerevisiae) | 0.81 | 2.00×10^-6^ | 8.22×10^-5^ |
| HIATL1 | transporter activity | AF255650 | hippocampus abundant transcript-like 1 | 1.97 | 2.00×10^-6^ | 8.22×10^-5^ |
| ZDHHC20 | zinc ion binding | BG249221 | zinc finger, DHHC-type containing 20 | 2.26 | 2.00×10^-6^ | 8.22×10^-5^ |
| PFKL | nucleotide binding | NM_002626 | phosphofructokinase, liver | 0.65 | 3.00×10^-6^ | 0.00011 |
| SRP54 | nucleotide binding | NM_003136 | signal recognition particle 54kDa | 1.56 | 3.00×10^-6^ | 0.00011 |
| SERPINA1 | serine-type endopeptidase inhibitor activity | AF119873 | serpin peptidase inhibitor, clade A (alpha-1 antiproteinase, antitrypsin), member 1 | 0.71 | 3.00×10^-6^ | 0.00011 |
| SLC25A46 | binding | M74089 | solute carrier family 25, member 46 | 2.20 | 3.00×10^-6^ | 0.00011 |
| ACTR3 | nucleotide binding | Z78330 | ARP3 actin-related protein 3 homolog (yeast) | 1.47 | 3.00×10^-6^ | 0.00011 |
| SHC1 | activation of MAPK activity | AI091079 | SHC (Src homology 2 domain containing) transforming protein 1 | 0.81 | 3.00×10^-6^ | 0.00011 |
| SCFD1 | cytoplasm | AB020724 | sec1 family domain containing 1 | 1.63 | 3.00×10^-6^\ | 0.00011 |
| PCNP | protein binding | NM_020357 | PEST proteolytic signal containing nuclear protein | 2.54 | 3.00×10^-6^ | 0.00011 |
| GALNT7 | Golgi membrane | NM_017423 | UDP-N-acetyl-alpha-D-galactosamine:polypeptide N-acetylgalactosaminyltransferase 7 (GalNAc-T7) | 1.87 | 3.00×10^-6^ | 0.00011 |
| RAP2C | nucleotide binding | NM_021183 | RAP2C, member of RAS oncogene family | 1.84 | 3.00×10^-6^ | 0.00011 |
| JMJD1C | iron ion binding | AA425650 | jumonji domain containing 1C | 1.94 | 3.00×10^-6^ | 0.00011 |
| CCNYL1 | --- | AK026922 | Cyclin Y-like 1 | 1.62 | 3.00×10^-6^ | 0.00011 |
| HIF1A | response to hypoxia | NM_001530 | hypoxia-inducible factor 1, alpha subunit (basic helix-loop-helix transcription factor) | 2.22 | 4.00×10^-6^ | 0.00013 |
| MBNL1 | in utero embryonic development | NM_021038 | muscleblind-like (Drosophila) | 2.16 | 4.00×10^-6^ | 0.00013 |
| POLR2B | magnesium ion binding | NM_000938 | polymerase (RNA) II (DNA directed) polypeptide B, 140kDa | 1.67 | 4.00×10^-6^ | 0.00013 |
| CCNG1 | regulation of cyclin-dependent protein kinase activity | BC000196 | cyclin G1 | 1.96 | 4.00×10^-6^ | 0.00013 |
| WBP2 | protein binding | U79458 | WW domain binding protein 2 | 0.54 | 4.00×10^-6^ | 0.00013 |
| ITGA4 | receptor activity | BG532690 | integrin, alpha 4 (antigen CD49D, alpha 4 subunit of VLA-4 receptor) | 2.24 | 4.00×10^-6^ | 0.00013 |
| LIMK2 | nucleotide binding | AC002073 | LIM domain kinase 2 | 0.33 | 4.00×10^-6^ | 0.00013 |
| NAGK | nucleotide binding | NM_017567 | N-acetylglucosamine kinase | 0.77 | 4.00×10^-6^ | 0.00013 |
| TRIM8 | protein binding | AF220034 | tripartite motif-containing 8 | 0.69 | 4.00×10^-6^ | 0.00013 |
| FGFR1OP2 | cytoplasm | AI738434 | FGFR1 oncogene partner 2 | 1.57 | 4.00×10^-6^ | 0.00013 |
| SCYL2 | protein kinase activity | AB037781 | SCY1-like 2 (S. cerevisiae) | 2.45 | 4.00×10^-6^ | 0.00013 |
| FBXO28 | ubiquitin cycle | AB007952 | F-box protein 28 | 1.45 | 5.00×10^-6^ | 0.00015 |
| UROD | uroporphyrinogen decarboxylase activity | M14016 | uroporphyrinogen decarboxylase | 0.74 | 5.00×10^-6^ | 0.00015 |
| MORF4L1 | regulation of cell growth | NM_006791 | mortality factor 4 like 1 | 1.71 | 5.00×10^-6^ | 0.00015 |
| CASC4 | membrane | BE904551 | cancer susceptibility candidate 4 | 2.03 | 5.00×10^-6^ | 0.00015 |
| RABEP1 | GTPase activator activity | AL550977 | rabaptin, RAB GTPase binding effector protein 1 | 1.42 | 5.00×10^-6^ | 0.00015 |
| SKAP2 | SH3/SH2 adaptor activity | N21390 | src kinase associated phosphoprotein 2 | 1.76 | 5.00×10^-6^ | 0.00015 |
| PIK3AP1 | kinase activity | AW575754 | phosphoinositide-3-kinase adaptor protein 1 | 1.70 | 5.00×10^-6^ | 0.00015 |
| PGK1 | nucleotide binding | NM_000291 | phosphoglycerate kinase 1 | 1.35 | 6.00×10^-6^ | 0.00017 |
| FKBP4 | peptidyl-prolyl cis-trans isomerase activity | NM_002014 | FK506 binding protein 4, 59kDa | 0.67 | 6.00×10^-6^ | 0.00017 |
| CUL5 | G1/S transition of mitotic cell cycle | BF435809 | cullin 5 | 2.25 | 6.00×10^-6^ | 0.00017 |
| ALKBH1 | DNA dealkylation | NM_006020 | alkB, alkylation repair homolog 1 (E. coli) | 1.30 | 6.00×10^-6^ | 0.00017 |
| TOR1AIP1 | membrane fraction | AK023204 | torsin A interacting protein 1 | 1.74 | 6.00×10^-6^ | 0.00017 |
| BXDC5 | membrane fraction | NM_025065 | brix domain containing 5 | 1.57 | 6.00×10^-6^ | 0.00017 |
| MAN1A1 | Golgi membrane | BG287153 | Mannosidase, alpha, class 1A, member 1 | 2.26 | 6.00×10^-6^ | 0.00017 |
| TRAPPC5 | protein binding | BF569208 | trafficking protein particle complex 5 | 0.63 | 6.00×10^-6^ | 0.00017 |
| ZNF710 | DNA binding | AI246730 | zinc finger protein 710 | 0.70 | 6.00×10^-6^ | 0.00017 |
| BZW1 | translation initiation factor activity | NM_014670 | basic leucine zipper and W2 domains 1 | 2.20 | 7.00×10^-6^ | 0.00019 |
| PIK3R1 | insulin receptor binding | AI679268 | phosphoinositide-3-kinase, regulatory subunit 1 (alpha) | 2.04 | 7.00×10^-6^ | 0.00019 |
| YTHDF3 | cytoplasm | AU157915 | YTH domain family, member 3 | 2.55 | 7.00×10^-6^ | 0.00019 |
| NOL11 | membrane fraction | AU158148 | nucleolar protein 11 | 1.69 | 7.00×10^-6^ | 0.00019 |
| PHPT1 | phosphoprotein phosphatase activity | AF285119 | phosphohistidine phosphatase 1 | 0.75 | 7.00×10^-6^ | 0.00019 |
| SLMAP | smooth endoplasmic reticulum | AB046821 | sarcolemma associated protein | 1.65 | 7.00×10^-6^ | 0.00019 |
| TGFBR2 | nucleotide binding | D50683 | transforming growth factor, beta receptor II (70/80kDa) | 1.49 | 8.00×10^-6^ | 0.00021 |
| RAP2A | nucleotide binding | AI302106 | RAP2A, member of RAS oncogene family | 1.75 | 8.00×10^-6^ | 0.00021 |
| RNF149 | protein binding | AL558987 | ring finger protein 149 | 1.51 | 8.00×10^-6^ | 0.00021 |
| SGTB | binding | AI376997 | small glutamine-rich tetratricopeptide repeat (TPR)-containing, beta | 2.64 | 8.00×10^-6^ | 0.00021 |
| ATP8A1 | nucleotide binding | AI769688 | ATPase, aminophospholipid transporter (APLT), class I, type 8A, member 1 | 2.11 | 9.00×10^-6^ | 0.00024 |
| POU2F1 | transcription factor activity | NM_002697 | POU class 2 homeobox 1 | 0.67 | 1.00×10^-5^ | 0.00025 |
| PIP5K3 | protein binding | AB023198 | phosphatidylinositol-3-phosphate/phosphatidylinositol 5-kinase, type III | 1.95 | 1.00×10^-5^ | 0.00025 |
| METTL9 | --- | NM_016025 | methyltransferase like 9 | 1.33 | 1.00×10^-5^ | 0.00025 |
| SS18L2 | --- | NM_016305 | synovial sarcoma translocation gene on chromosome 18-like 2 | 1.48 | 1.00×10^-5^ | 0.00025 |
| SHARPIN | intracellular | NM_030974 | SHANK-associated RH domain interactor | 0.47 | 1.00×10^-5^ | 0.00025 |
| EIF2A | tRNA binding | AF212241 | eukaryotic translation initiation factor 2A, 65kDa | 1.54 | 1.00×10^-5^ | 0.00025 |
| PRKACA | nucleotide binding | NM_002730 | solute carrier family 1 (glial high affinity glutamate transporter), member 3 | 0.59 | 1.10×10^-5^ | 0.00026 |
| AKAP11 | protein binding | NM_016248 | A kinase (PRKA) anchor protein 11 | 2.23 | 1.10×10^-5^ | 0.00026 |
| ITGB1 | ruffle | BG500301 | integrin, beta 1 (fibronectin receptor, beta polypeptide, antigen CD29 includes MDF2, MSK12) | 2.20 | 1.10×10^-5^ | 0.00026 |
| CDC2L2 | nucleotide binding | AI767436 | cell division cycle 2-like 2 (PITSLRE proteins) | 0.72 | 1.10×10^-5^ | 0.00026 |
| PTPLB | protein binding | AV712602 | protein tyrosine phosphatase-like (proline instead of catalytic arginine), member b | 1.72 | 1.10×10^-5^ | 0.00026 |
| CCDC109A | membrane | AA579630 | coiled-coil domain containing 109A | 1.45 | 1.10×10^-5^ | 0.00026 |
| XRN1 | DNA binding | BG534738 | 5'-3' exoribonuclease 1 | 1.67 | 1.10×10^-5^ | 0.00026 |
| AP2A1 | protein binding | AC006942 | adaptor-related protein complex 2, alpha 1 subunit | 0.49 | 1.10×10^-5^ | 0.00026 |
| SF3B1 | nuclear mRNA splicing, via spliceosome | NM_012433 | splicing factor 3b, subunit 1, 155kDa | 1.75 | 1.20×10^-5^ | 0.00028 |
| COASY | nucleotide binding | NM_025233 | Coenzyme A synthase | 0.78 | 1.20×10^-5^ | 0.00028 |
| COX5B | cytochrome-c oxidase activity | NM_001862 | cytochrome c oxidase subunit Vb | 0.70 | 1.20×10^-5^ | 0.00028 |
| MBD4 | DNA binding | AF114784 | methyl-CpG binding domain protein 4 | 1.44 | 1.20×10^-5^ | 0.00028 |
| SLAIN2 | --- | AU159395 | SLAIN motif family, member 2 | 1.34 | 1.20×10^-5^ | 0.00028 |
| SPIN1 | membrane fraction | AL136719 | spindlin 1 | 1.71 | 1.40×10^-5^ | 0.00032 |
| FBXL3 | ubiquitin ligase complex | AI983021 | F-box and leucine-rich repeat protein 3 | 2.18 | 1.40×10^-5^ | 0.00032 |
| TMEM41A | membrane | BE644935 | transmembrane protein 41A | 1.43 | 1.40×10^-5^ | 0.00032 |
| LTB4R | nucleotide binding | U41070 | leukotriene B4 receptor | 0.68 | 1.50×10^-5^ | 0.00034 |
| TGFBR1 | nucleotide binding | AA604375 | transforming growth factor, beta receptor 1 | 2.57 | 1.50×10^-5^ | 0.00034 |
| NBR1 | zinc ion binding | BC012591 | Neighbor of BRCA1 gene 2 | 1.71 | 1.60×10^-5^ | 0.00034 |
| USO1 | Golgi membrane | NM_003715 | USO1 homolog, vesicle docking protein (yeast) | 1.76 | 1.60×10^-5^ | 0.00034 |
| FES | nucleotide binding | NM_002005 | feline sarcoma oncogene | 0.67 | 1.60×10^-5^ | 0.00034 |
| CD46 | inner acrosomal membrane | AL570661 | CD46 molecule, complement regulatory protein | 2.11 | 1.60×10^-5^ | 0.00034 |
| ZFAND5 | molecular_function | AF062347 | zinc finger, AN1-type domain 5 | 1.77 | 1.60×10^-5^ | 0.00034 |
| UQCRC2 | metalloendopeptidase activity | AV727381 | ubiquinol-cytochrome c reductase core protein II | 1.38 | 1.60×10^-5^ | 0.00034 |
| ETNK1 | ethanolamine kinase activity | AV692425 | ethanolamine kinase 1 | 3.22 | 1.60×10^-5^ | 0.00034 |
| MAP2K7 | nucleotide binding | AI090153 | mitogen-activated protein kinase kinase 7 | 0.64 | 1.60×10^-5^ | 0.00034 |
| FURIN | furin activity | NM_002569 | furin (paired basic amino acid cleaving enzyme) | 0.66 | 1.70×10^-5^ | 0.00036 |
| TSPAN32 | molecular_function | NM_005705 | tetraspanin 32 | 0.81 | 1.70×10^-5^ | 0.00036 |
| DEK | DNA binding | NM_003472 | DEK oncogene | 2.40 | 1.80×10^-5^ | 0.00038 |
| PAPOLA | RNA binding | AI670847 | poly(A) polymerase alpha | 2.67 | 1.80×10^-5^ | 0.00038 |
| ENO2 | phosphopyruvate hydratase complex | NM_001975 | enolase 2 (gamma, neuronal) | 1.49 | 1.90×10^-5^ | 0.00039 |
| MPHOSPH6 | M phase of mitotic cell cycle | NM_005792 | M-phase phosphoprotein 6 | 1.40 | 1.90×10^-5^ | 0.00039 |
| RASGRP1 | Ras guanyl-nucleotide exchange factor activity | NM_005739 | RAS guanyl releasing protein 1 (calcium and DAG-regulated) | 2.00 | 1.90×10^-5^ | 0.00039 |
| RIN3 | GTPase activator activity | AW027923 | Ras and Rab interactor 3 | 0.55 | 1.90×10^-5^ | 0.00039 |
| VWCE | sugar binding | AI862096 | von Willebrand factor C and EGF domains | 0.33 | 1.90×10^-5^ | 0.00039 |
| CEP57 | protein import into nucleus, translocation | NM_014679 | centrosomal protein 57kDa | 1.79 | 2.00×10^-5^ | 0.00040 |
| CEBPG | liver development | BE622659 | CCAAT/enhancer binding protein (C/EBP), gamma | 1.59 | 2.00×10^-5^ | 0.00040 |
| MAP3K7 | nucleotide binding | AF218074 | mitogen-activated protein kinase kinase kinase 7 | 1.98 | 2.10×10^-5^ | 0.00042 |
| E2F4 | regulation of transcription of G1/S-phase of mitotic cell cycle | BC000110 | E2F transcription factor 4, p107/p130-binding | 0.80 | 2.20×10^-5^ | 0.00043 |
| ANKRD49 | --- | NM_017704 | ankyrin repeat domain 49 | 1.73 | 2.20×10^-5^ | 0.00043 |
| STK11IP | protein binding | AV727346 | serine/threonine kinase 11 interacting protein | 0.74 | 2.20×10^-5^ | 0.00043 |
| CPEB2 | nucleotide binding | AI202327 | cytoplasmic polyadenylation element binding protein 2 | 2.76 | 2.20×10^-5^ | 0.00043 |
| ECHDC1 | catalytic activity | AK021464 | enoyl Coenzyme A hydratase domain containing 1 | 1.53 | 2.20×10^-5^ | 0.00043 |
| VPS13B | protein transport | BE048857 | vacuolar protein sorting 13 homolog B (yeast) | 1.77 | 2.20×10^-5^ | 0.00043 |
| PIK3C2A | inositol or phosphatidylinositol kinase activity | AV682436 | phosphoinositide-3-kinase, class 2, alpha polypeptide | 2.05 | 2.30×10^-5^ | 0.00044 |
| ANP32E | protein binding | AW612574 | acidic (leucine-rich) nuclear phosphoprotein 32 family, member E | 1.60 | 2.30×10^-5^ | 0.00044 |
| TRAF7 | ubiquitin ligase complex | AL136921 | TNF receptor-associated factor 7 | 0.84 | 2.30×10^-5^ | 0.00044 |
| UBE2N | double-strand break repair via homologous recombination | NM_003348 | ubiquitin-conjugating enzyme E2N (UBC13 homolog, yeast) | 1.42 | 2.40×10^-5^ | 0.00045 |
| STK17A | nucleotide binding | NM_004760 | serine/threonine kinase 17a | 1.50 | 2.40×10^-5^ | 0.00045 |
| CNOT7 | nucleic acid binding | W94952 | CCR4-NOT transcription complex, subunit 7 | 1.74 | 2.40×10^-5^ | 0.00045 |
| IDS | iduronate-2-sulfatase activity | NM_000202 | iduronate 2-sulfatase | 1.46 | 2.50×10^-5^ | 0.00046 |
| ZER1 | ubiquitin-protein ligase activity | AI991574 | zer-1 homolog (C. elegans) | 0.50 | 2.50×10^-5^ | 0.00046 |
| CEP350 | membrane fraction | AI041204 | centrosomal protein 350kDa | 1.82 | 2.50×10^-5^ | 0.00046 |
| CYBA | iron ion binding | NM_000101 | cytochrome b-245, alpha polypeptide | 0.84 | 2.60×10^-5^ | 0.00048 |
| ACAA2 | acetyl-CoA C-acyltransferase activity | NM_006111 | acetyl-Coenzyme A acyltransferase 2 | 1.54 | 2.70×10^-5^ | 0.00049 |
| SGK3 | nucleotide binding | AV690866 | serum/glucocorticoid regulated kinase family, member 3 | 1.62 | 2.70×10^-5^ | 0.00049 |
| RAD23A | single-stranded DNA binding | BF572938 | RAD23 homolog A (S. cerevisiae) | 0.55 | 2.80×10^-5^ | 0.00051 |
| UBE4A | ubiquitin ligase complex | NM_004788 | ubiquitination factor E4A (UFD2 homolog, yeast) | 1.52 | 2.80×10^-5^ | 0.00051 |
| GOLGB1 | Golgi membrane | N53479 | golgin B1, golgi integral membrane protein | 0.82 | 2.90×10^-5^ | 0.00052 |
| DEDD | DNA binding | AK022531 | death effector domain containing | 0.78 | 2.90×10^-5^ | 0.00052 |
| HNRNPA0 | nucleotide binding | BE966599 | heterogeneous nuclear ribonucleoprotein A0 | 1.49 | 3.00×10^-5^ | 0.00053 |
| ZC3H11A | nucleic acid binding | NM_014827 | zinc finger CCCH-type containing 11A | 1.39 | 3.00×10^-5^ | 0.00053 |
| CRAT | carnitine O-acetyltransferase activity | BC000723 | carnitine acetyltransferase | 0.59 | 3.00×10^-5^ | 0.00053 |
| ABI1 | intracellular | BF673013 | abl-interactor 1 | 1.79 | 3.10×10^-5^ | 0.00054 |
| FMR1 | mRNA binding | AA830884 | fragile X mental retardation 1 | 2.43 | 3.10×10^-5^ | 0.00054 |
| ANKRD12 | membrane fraction | AB020681 | ankyrin repeat domain 12 | 1.70 | 3.20×10^-5^ | 0.00056 |
| SLC25A37 | iron ion transmembrane transporter activity | N63920 | solute carrier family 25, member 37 | 0.76 | 3.20×10^-5^ | 0.00056 |
| PLCG1 | in utero embryonic development | AL110247 | phospholipase C, gamma 1 | 0.79 | 3.30×10^-5^ | 0.00057 |
| RAPGEF6 | guanyl-nucleotide exchange factor activity | AI640834 | Rap guanine nucleotide exchange factor (GEF) 6 | 2.26 | 3.30×10^-5^ | 0.00057 |
| LILRB1 | receptor activity | NM_006669 | leukocyte immunoglobulin-like receptor, subfamily B (with TM and ITIM domains), member 1 | 0.70 | 3.40×10^-5^ | 0.00058 |
| SLC6A8 | neurotransmitter uptake | NM_005629 | solute carrier family 6 (neurotransmitter transporter, creatine), member 8 | 0.36 | 3.50×10^-5^ | 0.00059 |
| GPN1 | --- | AB044661 | GPN-loop GTPase 1 | 1.35 | 3.50×10^-5^ | 0.00059 |
| TOLLIP | signal transducer activity | NM_019009 | toll interacting protein | 0.48 | 3.60×10^-5^ | 0.00060 |
| SP1 | double-stranded DNA binding | AI795991 | Sp1 transcription factor | 0.69 | 3.60×10^-5^ | 0.00060 |
| RALA | nucleotide binding | AV703462 | v-ral simian leukemia viral oncogene homolog A (ras related) | 1.38 | 3.60×10^-5^ | 0.00060 |
| CTBP2 | membrane fraction | NM_001329 | C-terminal binding protein 2 | 1.32 | 3.70×10^-5^ | 0.00061 |
| SNX2 | protein binding | AF043453 | sorting nexin 2 | 1.72 | 3.70×10^-5^ | 0.00061 |
| TDG | damaged DNA binding | NM_003211 | thymine-DNA glycosylase | 1.69 | 3.70×10^-5^ | 0.00061 |
| FOXN2 | transcription factor activity | BF590117 | forkhead box N2 | 2.31 | 3.90×10^-5^ | 0.00064 |
| FKBP8 | cell fate specification | L37033 | FK506 binding protein 8, 38kDa | 0.26 | 3.90×10^-5^ | 0.00064 |
| HMGN1 | chromatin | NM_004965 | high-mobility group nucleosome binding domain 1 | 1.49 | 4.10×10^-5^ | 0.00066 |
| TFRC | receptor activity | NM_003234 | transferrin receptor (p90, CD71) | 1.47 | 4.10×10^-5^ | 0.00066 |
| MAT2B | catalytic activity | NM_013283 | methionine adenosyltransferase II, beta | 1.70 | 4.20×10^-5^ | 0.00067 |
| H2AFV | nucleosome | AI823792 | H2A histone family, member V | 1.29 | 4.20×10^-5^ | 0.00067 |
| RGS14 | receptor signaling protein activity | AF037195 | regulator of G-protein signaling 14 | 0.82 | 4.20×10^-5^ | 0.00067 |
| ZYG11B | binding | AV701229 | zyg-11 homolog B (C. elegans) | 1.48 | 4.30×10^-5^ | 0.00069 |
| JUND | chromatin | NM_005354 | jun D proto-oncogene | 0.80 | 4.50×10^-5^ | 0.00071 |
| RNF24 | protein binding | NM_007219 | ring finger protein 24 | 0.56 | 4.50×10^-5^ | 0.00071 |
| CRLS1 | mitochondrion | AF241784 | cardiolipin synthase 1 | 1.69 | 4.50×10^-5^ | 0.00071 |
| SIRPB2 | receptor activity | AK095499 | signal-regulatory protein beta 2 | 0.67 | 4.60×10^-5^ | 0.00072 |
| SLC30A1 | in utero embryonic development | AI972416 | Solute carrier family 30 (zinc transporter), member 1 | 2.48 | 4.60×10^-5^ | 0.00072 |
| TBPL1 | DNA binding | NM_004865 | TBP-like 1 | 1.59 | 4.70×10^-5^ | 0.00072 |
| MAN2A1 | Golgi membrane | AV700323 | mannosidase, alpha, class 2A, member 1 | 1.65 | 4.70×10^-5^ | 0.00072 |
| GPR177 | signal transducer activity | AL534095 | G protein-coupled receptor 177 | 0.74 | 4.70×10^-5^ | 0.00072 |
| CCR3 | rhodopsin-like receptor activity | NM_001837 | chemokine (C-C motif) receptor 3 | 2.13 | 4.80×10^-5^ | 0.00073 |
| CNP | 2',3'-cyclic-nucleotide 3'-phosphodiesterase activity | BC001362 | 2',3'-cyclic nucleotide 3' phosphodiesterase | 0.78 | 4.80×10^-5^ | 0.00073 |
| TMEM30A | membrane | NM_018247 | transmembrane protein 30A | 2.24 | 4.80×10^-5^ | 0.00073 |
| HISPPD1 | acid phosphatase activity | NM_015216 | histidine acid phosphatase domain containing 1 | 2.04 | 4.90×10^-5^ | 0.00074 |
| LSM10 | RNA binding | AL542359 | LSM10, U7 small nuclear RNA associated | 0.74 | 4.90×10^-5^ | 0.00074 |
| PYROXD1 | electron transport | NM_024854 | pyridine nucleotide-disulphide oxidoreductase domain 1 | 1.63 | 5.20×10^-5^ | 0.00078 |
| API5 | binding | NM_006595 | apoptosis inhibitor 5 | 1.69 | 5.30×10^-5^ | 0.00079 |
| OCIAD1 | endosome | AF323665 | OCIA domain containing 1 | 1.51 | 5.30×10^-5^ | 0.00079 |
| ZNF22 | DNA binding | AA744771 | zinc finger protein 22 (KOX 15) | 1.62 | 5.50×10^-5^ | 0.00082 |
| CACYBP | protein binding | BC005975 | calcyclin binding protein | 1.45 | 5.60×10^-5^ | 0.00083 |
| 2-Mar | protein binding | AF151074 | membrane-associated ring finger (C3HC4) 2 | 0.48 | 5.70×10^-5^ | 0.00084 |
| RP5-1077B9.4 | --- | W60802 | invasion inhibitory protein 45 | 0.63 | 5.70×10^-5^ | 0.00084 |
| UBL3 | intracellular | AF044221 | ubiquitin-like 3 | 2.02 | 5.80×10^-5^ | 0.00085 |
| GDI1 | Rab GDP-dissociation inhibitor activity | NM_001493 | GDP dissociation inhibitor 1 | 0.64 | 5.90×10^-5^ | 0.00086 |
| DCUN1D1 | molecular_function | N58513 | DCN1, defective in cullin neddylation 1, domain containing 1 (S. cerevisiae) | 0.61 | 5.90×10^-5^ | 0.00086 |
| TIA1 | nucleotide binding | AL046419 | TIA1 cytotoxic granule-associated RNA binding protein | 2.24 | 6.00×10^-5^ | 0.00086 |
| SAPS3 | protein phosphatase binding | AK023950 | SAPS domain family, member 3 | 1.86 | 6.00×10^-5^ | 0.00086 |
| WDSOF1 | membrane fraction | BE409290 | WD repeats and SOF1 domain containing | 1.64 | 6.00×10^-5^ | 0.00086 |
| CD5 | scavenger receptor activity | AI797836 | CD5 molecule | 1.33 | 6.00×10^-5^ | 0.00086 |
| PA2G4 | transcription factor activity | U87954 | proliferation-associated 2G4, 38kDa | 0.79 | 6.10×10^-5^ | 0.00087 |
| ADD3 | structural constituent of cytoskeleton | BE545756 | adducin 3 (gamma) | 1.82 | 6.30×10^-5^ | 0.00089 |
| COPS3 | in utero embryonic development | NM_003653 | COP9 constitutive photomorphogenic homolog subunit 3 (Arabidopsis) | 1.31 | 6.30×10^-5^ | 0.00089 |
| HMG20B | nucleotide binding | BC002552 | high-mobility group 20B | 0.71 | 6.30×10^-5^ | 0.00089 |
| NFE2L1 | transcription factor activity | NM_003204 | nuclear factor (erythroid-derived 2)-like 1 | 0.85 | 6.40×10^-5^ | 0.00089 |
| TUBA1B | nucleotide binding | NM_006082 | tubulin, alpha 1b | 0.82 | 6.40×10^-5^ | 0.00089 |
| MRPS35 | mitochondrion | NM_021821 | mitochondrial ribosomal protein S35 | 1.51 | 6.50×10^-5^ | 0.00090 |
| SEMA5B | receptor activity | BC002776 | sema domain, seven thrombospondin repeats (type 1 and type 1-like), transmembrane domain (TM) and short cytoplasmic domain, (semaphorin) 5B | 0.77 | 6.50×10^-5^ | 0.00090 |
| CRLF3 | --- | NM_015986 | cytokine receptor-like factor 3 | 1.61 | 6.60×10^-5^ | 0.00090 |
| CASP4 | protein binding | AL050391 | caspase 4, apoptosis-related cysteine peptidase | 0.68 | 6.60×10^-5^ | 0.00090 |
| MT1F | copper ion binding | M10943 | metallothionein 1F | 0.69 | 6.60×10^-5^ | 0.00090 |
| ST6GALNAC4 | Golgi membrane | AW044319 | ST6 (alpha-N-acetyl-neuraminyl-2,3-beta-galactosyl-1,3)-N-acetylgalactosaminide alpha-2,6-sialyltransferase 4 | 0.38 | 6.70×10^-5^ | 0.00091 |
| FYTTD1 | --- | BF576005 | forty-two-three domain containing 1 | 2.12 | 6.70×10^-5^ | 0.00091 |
| MAP3K1 | nucleotide binding | AA541479 | mitogen-activated protein kinase kinase kinase 1 | 1.76 | 6.70×10^-5^ | 0.00091 |
| SRBD1 | RNA binding | NM_018079 | S1 RNA binding domain 1 | 1.63 | 6.90×10^-5^ | 0.00093 |
| PHF23 | protein binding | AY099328 | PHD finger protein 23 | 0.75 | 7.20×10^-5^ | 0.00096 |
| CCNG2 | cell cycle checkpoint | AW134535 | cyclin G2 | 2.34 | 7.20×10^-5^ | 0.00096 |
| CANX | angiogenesis | M94859 | calnexin | 1.32 | 7.50×10^-5^ | 0.0010 |
| TNS1 | actin binding | AF116610 | tensin 1 | 0.47 | 7.50×10^-5^ | 0.0010 |
| LIN7C | protein binding | N63709 | lin-7 homolog C (C. elegans) | 1.50 | 7.60×10^-5^ | 0.0010 |
| TM9SF4 | membrane | AI418892 | transmembrane 9 superfamily protein member 4 | 0.71 | 7.70×10^-5^ | 0.0010 |
| TPP1 | aminopeptidase activity | BG231932 | tripeptidyl peptidase I | 0.67 | 7.80×10^-5^ | 0.0010 |
| CLK4 | nucleotide binding | AF212224 | CDC-like kinase 4 | 1.84 | 7.90×10^-5^ | 0.0010 |
| GCC2 | Golgi membrane | NM_014635 | glutathione peroxidase 2 (gastrointestinal) | 1.67 | 8.10×10^-5^ | 0.0010 |
| VDAC3 | protein binding | U90943 | voltage-dependent anion channel 3 | 1.35 | 8.10×10^-5^ | 0.0010 |
| RPL22 | RNA binding | BE250348 | ribosomal protein L22 | 1.42 | 8.10×10^-5^ | 0.0010 |
| KREMEN1 | molecular_function | BF221745 | kringle containing transmembrane protein 1 | 0.56 | 8.10×10^-5^ | 0.0010 |
| PPWD1 | peptidyl-prolyl cis-trans isomerase activity | AK025679 | peptidylprolyl isomerase domain and WD repeat containing 1 | 1.60 | 8.30×10^-5^ | 0.0011 |
| RAB18 | nucleotide binding | AI769954 | RAB18, member RAS oncogene family | 1.80 | 8.40×10^-5^ | 0.0011 |
| NEK7 | nucleotide binding | AL080111 | NIMA (never in mitosis gene a)-related kinase 7 | 3.19 | 8.60×10^-5^ | 0.0011 |
| RBM18 | nucleotide binding | AA167623 | RNA binding motif protein 18 | 1.56 | 8.60×10^-5^ | 0.0011 |
| BIRC2 | response to hypoxia | NM_001166 | baculoviral IAP repeat-containing 2 | 1.71 | 8.80×10^-5^ | 0.0011 |
| NFKBIB | transcription coactivator activity | NM_002503 | nuclear factor of kappa light polypeptide gene enhancer in B-cells inhibitor, beta | 0.68 | 8.80×10^-5^ | 0.0011 |
| LRRC58 | protein binding | AL524175 | leucine rich repeat containing 58 | 1.47 | 8.80×10^-5^ | 0.0011 |
| KAT2B | --- | AV727449 | K(lysine) acetyltransferase 2B | 2.15 | 8.90×10^-5^ | 0.0011 |
| CHUK | nucleotide binding | AF080157 | conserved helix-loop-helix ubiquitous kinase | 1.71 | 8.90×10^-5^ | 0.0011 |
| PION | --- | AK026747 | pigeon homolog (Drosophila) | 1.66 | 9.00×10^-5^ | 0.0011 |
| CTSB | cathepsin B activity | NM_001908 | cathepsin B | 0.84 | 9.20×10^-5^ | 0.0011 |
| PTGER4 | rhodopsin-like receptor activity | AA897516 | prostaglandin E receptor 4 (subtype EP4) | 1.75 | 9.20×10^-5^ | 0.0011 |
| FLOT2 | protein binding | BC003683 | flotillin 2 | 0.43 | 9.40×10^-5^ | 0.0012 |
| SRXN1 | nucleotide binding | AL121758 | sulfiredoxin 1 homolog (S. cerevisiae) | 1.45 | 9.40×10^-5^ | 0.0012 |
| DHX15 | nucleotide binding | NM_001358 | DEAH (Asp-Glu-Ala-His) box polypeptide 15 | 1.57 | 9.70×10^-5^ | 0.0012 |
| MPV17 | mitochondrial genome maintenance | NM_002437 | MpV17 mitochondrial inner membrane protein | 0.86 | 9.70×10^-5^ | 0.0012 |
| RPL15 | RNA binding | AF279903 | ribosomal protein L15 | 1.41 | 0.00010 | 0.0013 |
| CSNK1A1 | nucleotide binding | AW268585 | casein kinase 1, alpha 1 | 1.68 | 0.00011 | 0.0013 |
| SMEK2 | protein binding | AK026437 | SMEK homolog 2, suppressor of mek1 (Dictyostelium) | 2.13 | 0.00011 | 0.0013 |
| MBTPS1 | Golgi membrane | NM_003791 | membrane-bound transcription factor peptidase, site 1 | 1.50 | 0.00011 | 0.0013 |
| UBR5 | ubiquitin-protein ligase activity | U69567 | ubiquitin protein ligase E3 component n-recognin 5 | 1.43 | 0.00011 | 0.0013 |
| ZNF12 | DNA binding | BM463827 | zinc finger protein 12 | 1.90 | 0.00011 | 0.0013 |
| TAF9 | DNA binding | NM_003187 | TAF9 RNA polymerase II, TATA box binding protein (TBP)-associated factor, 32kDa | 1.63 | 0.00011 | 0.0013 |
| PER2 | signal transducer activity | NM_022817 | period homolog 2 (Drosophila) | 1.40 | 0.00011 | 0.0013 |
| GGA3 | protein binding | AW008018 | golgi associated, gamma adaptin ear containing, ARF binding protein 3 | 0.83 | 0.00012 | 0.0014 |
| ZBED5 | DNA binding | NM_021211 | zinc finger, BED-type containing 5 | 1.68 | 0.00012 | 0.0014 |
| ELOF1 | membrane fraction | AK001171 | elongation factor 1 homolog (S. cerevisiae) | 0.66 | 0.00012 | 0.0014 |
| SMARCAD1 | regulation of DNA recombination | AI807204 | SWI/SNF-related, matrix-associated actin-dependent regulator of chromatin, subfamily a, containing DEAD/H box 1 | 1.43 | 0.00013 | 0.0015 |
| AKIRIN1 | --- | BG109865 | akirin 1 | 1.80 | 0.00013 | 0.0015 |
| PPP4R2 | protein binding | AI983837 | protein phosphatase 4, regulatory subunit 2 | 2.14 | 0.00013 | 0.0016 |
| PSEN1 | Golgi membrane | NM_007318 | presenilin 1 | 1.31 | 0.00013 | 0.0016 |
| SNX3 | protein binding | BE962615 | sorting nexin 3 | 1.39 | 0.00014 | 0.0016 |
| FNDC3A | response to hypoxia | NM_014923 | fibronectin type III domain containing 3A | 2.29 | 0.00014 | 0.0016 |
| DDX17 | nucleotide binding | Z97056 | DEAD (Asp-Glu-Ala-Asp) box polypeptide 17 | 1.36 | 0.00014 | 0.0016 |
| USP33 | ubiquitin thiolesterase activity | AB029020 | ubiquitin specific peptidase 33 | 1.68 | 0.00014 | 0.0016 |
| SCP2 | sterol carrier activity | NM_002979 | sterol carrier protein 2 | 1.64 | 0.00014 | 0.0016 |
| REEP5 | molecular_function | BC000232 | receptor accessory protein 5 | 1.61 | 0.00015 | 0.0017 |
| DERL1 | receptor activity | AF131854 | Der1-like domain family, member 1 | 1.81 | 0.00015 | 0.0017 |
| TXN2 | mitochondrion | AL022313 | thioredoxin 2 | 0.64 | 0.00015 | 0.0017 |
| ARL6IP5 | protein binding | NM_006407 | ADP-ribosylation-like factor 6 interacting protein 5 | 1.88 | 0.00016 | 0.0017 |
| MED25 | membrane fraction | NM_030973 | mediator complex subunit 25 | 0.60 | 0.00016 | 0.0017 |
| SNX10 | protein binding | NM_013322 | sorting nexin 10 | 2.29 | 0.00016 | 0.0017 |
| IL1RN | interleukin-1 receptor antagonist activity | AW083357 | interleukin 1 receptor antagonist | 0.60 | 0.00016 | 0.0018 |
| TYMP | --- | NM_001953 | thymidine phosphorylase | 0.39 | 0.00016 | 0.0018 |
| HNRNPH3 | --- | AF132362 | heterogeneous nuclear ribonucleoprotein H3 (2H9) | 1.74 | 0.00016 | 0.0018 |
| MPST | thiosulfate sulfurtransferase activity | NM_021126 | mercaptopyruvate sulfurtransferase | 0.73 | 0.00016 | 0.0018 |
| EHBP1L1 | --- | BG334196 | EH domain binding protein 1-like 1 | 0.49 | 0.00016 | 0.0018 |
| UBE3A | ubiquitin-protein ligase activity | AA160522 | ubiquitin protein ligase E3A | 1.66 | 0.00016 | 0.0018 |
| PHF10 | protein binding | NM_018288 | PHD finger protein 10 | 1.38 | 0.00016 | 0.0018 |
| GNB5 | GTPase activity | NM_016194 | guanine nucleotide binding protein (G protein), beta 5 | 1.54 | 0.00017 | 0.0018 |
| MRPS22 | molecular_function | NM_020191 | mitochondrial ribosomal protein S22 | 1.41 | 0.00017 | 0.0018 |
| SBNO2 | --- | NM_014963 | strawberry notch homolog 2 (Drosophila) | 0.46 | 0.00017 | 0.0018 |
| SLC30A6 | Golgi apparatus | BF793552 | solute carrier family 30 (zinc transporter), member 6 | 1.48 | 0.00017 | 0.0018 |
| PPT1 | protein depalmitoylation | NM_000310 | palmitoyl-protein thioesterase 1 | 1.36 | 0.00017 | 0.0018 |
| ZSCAN29 | transcription factor activity | BE622486 | zinc finger and SCAN domain containing 29 | 1.49 | 0.00017 | 0.0018 |
| ACSL3 | magnesium ion binding | NM_004457 | acyl-CoA synthetase long-chain family member 3 | 1.75 | 0.00017 | 0.0018 |
| DHX34 | nucleotide binding | AA039439 | DEAH (Asp-Glu-Ala-His) box polypeptide 34 | 0.67 | 0.00017 | 0.0018 |
| PRKACB | nucleotide binding | AA130247 | protein kinase, cAMP-dependent, catalytic, beta | 2.29 | 0.00018 | 0.0019 |
| UPF1 | nucleotide binding | U59323 | UPF1 regulator of nonsense transcripts homolog (yeast) | 0.69 | 0.00018 | 0.0019 |
| ZMAT3 | regulation of cell growth | NM_022470 | zinc finger, matrin type 3 | 0.70 | 0.00018 | 0.0019 |
| NEDD1 | --- | AL832468 | neural precursor cell expressed, developmentally down-regulated 1 | 1.56 | 0.00018 | 0.0019 |
| BTAF1 | nucleotide binding | AJ001017 | BTAF1 RNA polymerase II, B-TFIID transcription factor-associated, 170kDa (Mot1 homolog, S. cerevisiae) | 1.56 | 0.00018 | 0.0019 |
| SON | DNA binding | BF676840 | SON DNA binding protein | 0.52 | 0.00018 | 0.0019 |
| CSK | nucleotide binding | NM_004383 | c-src tyrosine kinase | 0.75 | 0.00018 | 0.0019 |
| DOK3 | insulin receptor binding | NM_024872 | docking protein 3 | 0.58 | 0.00019 | 0.0020 |
| KLHL7 | protein binding | AL136597 | kelch-like 7 (Drosophila) | 1.63 | 0.00019 | 0.0020 |
| CLDND1 | membrane | AF161522 | claudin domain containing 1 | 1.79 | 0.00019 | 0.0020 |
| PWWP2A | --- | AI686303 | PWWP domain containing 2A | 1.47 | 0.00019 | 0.0020 |
| SKIV2L2 | nucleotide binding | AA525163 | superkiller viralicidic activity 2-like 2 (S. cerevisiae) | 1.63 | 0.00019 | 0.0020 |
| SLC35A2 | Golgi membrane | D84454 | solute carrier family 35 (UDP-galactose transporter), member A2 | 0.85 | 0.00020 | 0.0020 |
| GBA2 | glucosylceramidase activity | AB046825 | glucosidase, beta (bile acid) 2 | 0.83 | 0.00020 | 0.0021 |
| RNF19A | microtubule cytoskeleton organization | AB029316 | ring finger protein 19A | 1.92 | 0.00020 | 0.0021 |
| AP3M2 | protein binding | NM_006803 | adaptor-related protein complex 3, mu 2 subunit | 1.30 | 0.00021 | 0.0021 |
| ACTN1 | integrin binding | BC003576 | actinin, alpha 1 | 0.60 | 0.00021 | 0.0022 |
| DGKA | diacylglycerol kinase activity | AF064771 | diacylglycerol kinase, alpha 80kDa | 0.61 | 0.00021 | 0.0022 |
| GAK | nucleotide binding | D88435 | cyclin G associated kinase | 0.81 | 0.00021 | 0.0022 |
| OFD1 | molecular_function | NM_003611 | oral-facial-digital syndrome 1 | 1.42 | 0.00022 | 0.0022 |
| COQ10B | mitochondrion | NM_025147 | coenzyme Q10 homolog B (S. cerevisiae) | 1.52 | 0.00022 | 0.0022 |
| LRRC8C | protein binding | BE877357 | leucine rich repeat containing 8 family, member C | 1.38 | 0.00022 | 0.0022 |
| SH3GLB2 | protein binding | NM_020145 | SH3-domain GRB2-like endophilin B2 | 0.58 | 0.00023 | 0.0023 |
| SIAH1 | membrane fraction | NM_003031 | seven in absentia homolog 1 (Drosophila) | 1.45 | 0.00023 | 0.0023 |
| BTF3L4 | --- | AL568049 | basic transcription factor 3-like 4 | 1.52 | 0.00023 | 0.0023 |
| CUL2 | G1/S transition of mitotic cell cycle | NM_003591 | cullin 2 | 1.50 | 0.00023 | 0.0023 |
| TNIK | nucleotide binding | AF172268 | TRAF2 and NCK interacting kinase | 1.52 | 0.00023 | 0.0023 |
| RRBP1 | receptor activity | AA706065 | ribosome binding protein 1 homolog 180kDa (dog) | 0.70 | 0.00023 | 0.0023 |
| ATP5A1 | nucleotide binding | AI587323 | ATP synthase, H+ transporting, mitochondrial F1 complex, alpha subunit 1, cardiac muscle | 1.29 | 0.00023 | 0.0023 |
| TBCE | microtubule cytoskeleton organization | NM_003193 | tubulin folding cofactor E | 1.39 | 0.00023 | 0.0023 |
| GAA | maltose metabolic process | NM_000152 | STAM binding protein | 0.67 | 0.00024 | 0.0023 |
| ARGLU1 | --- | NM_018011 | arginine and glutamate rich 1 | 1.81 | 0.00024 | 0.0024 |
| EPN1 | membrane fraction | AK022454 | epsin 1 | 0.56 | 0.00024 | 0.0024 |
| SLC25A36 | transporter activity | AI694452 | solute carrier family 25, member 36 | 2.06 | 0.00025 | 0.0024 |
| SYNE1 | actin binding | AF043290 | spectrin repeat containing, nuclear envelope 1 | 1.39 | 0.00025 | 0.0024 |
| ARL6IP2 | nucleotide binding | AV700003 | ADP-ribosylation factor-like 6 interacting protein 2 | 1.43 | 0.00026 | 0.0025 |
| NPM1 | transcription coactivator activity | AB042278 | nucleophosmin (nucleolar phosphoprotein B23, numatrin) | 1.62 | 0.00026 | 0.0025 |
| NCOA7 | protein binding | AL035689 | nuclear receptor coactivator 7 | 1.38 | 0.00026 | 0.0025 |
| DENR | molecular_function | AF103800 | density-regulated protein | 1.51 | 0.00026 | 0.0025 |
| USP47 | ubiquitin thiolesterase activity | BE966019 | ubiquitin specific peptidase 47 | 1.38 | 0.00027 | 0.0026 |
| CHURC1 | transcription | BE568660 | churchill domain containing 1 | 1.67 | 0.00027 | 0.0026 |
| MIF4GD | protein binding | NM_020679 | MIF4G domain containing | 0.81 | 0.00027 | 0.0026 |
| SMARCD2 | transcription coactivator activity | AF113019 | SWI/SNF related, matrix associated, actin dependent regulator of chromatin, subfamily d, member 2 | 0.68 | 0.00027 | 0.0026 |
| RYK | nucleotide binding | NM_002958 | hypothetical protein FLJ11506 | 1.81 | 0.00028 | 0.0026 |
| ACP2 | skeletal system development | NM_001610 | acid phosphatase 2, lysosomal | 0.76 | 0.00028 | 0.0026 |
| UBE2NL | small conjugating protein ligase activity | AL109622 | ubiquitin-conjugating enzyme E2N-like | 1.32 | 0.00028 | 0.0026 |
| ATP5E | mitochondrion | NM_006886 | ATP synthase, H+ transporting, mitochondrial F1 complex, epsilon subunit | 0.53 | 0.00028 | 0.0026 |
| LRRC47 | RNA binding | AB033011 | leucine rich repeat containing 47 | 0.79 | 0.00028 | 0.0027 |
| SAMD3 | --- | AI129628 | sterile alpha motif domain containing 3 | 1.57 | 0.00029 | 0.0027 |
| BMP2K | nucleotide binding | AI735391 | BMP2 inducible kinase | 1.83 | 0.00029 | 0.0027 |
| RBM15 | nucleotide binding | AF364037 | RNA binding motif protein 15 | 1.44 | 0.00029 | 0.0027 |
| FCER1G | positive regulation of type IIa hypersensitivity | NM_004106 | Fc fragment of IgE, high affinity I, receptor for; gamma polypeptide | 0.78 | 0.00029 | 0.0027 |
| LY75 | receptor activity | NM_002349 | lymphocyte antigen 75 | 1.80 | 0.00030 | 0.0028 |
| SFRS3 | nucleotide binding | BC000914 | splicing factor, arginine/serine-rich 3 | 1.56 | 0.00030 | 0.0028 |
| TTYH3 | ion channel activity | AI934753 | tweety homolog 3 (Drosophila) | 0.67 | 0.00030 | 0.0028 |
| VAMP4 | Golgi membrane | AF052100 | vesicle-associated membrane protein 4 | 1.48 | 0.00030 | 0.0028 |
| ZNF559 | DNA binding | BC006436 | zinc finger protein 559 | 1.52 | 0.00030 | 0.0028 |
| PRPF19 | ubiquitin ligase complex | NM_014502 | PRP19/PSO4 pre-mRNA processing factor 19 homolog (S. cerevisiae) | 0.71 | 0.00031 | 0.0028 |
| HSPA6 | nucleotide binding | X51757 | heat shock 70kDa protein 6 (HSP70B') | 0.69 | 0.00031 | 0.0028 |
| LAMP2 | membrane fraction | NM_013995 | lysosomal-associated membrane protein 2 | 1.75 | 0.00031 | 0.0028 |
| DIMT1L | rRNA modification | AF091078 | DIM1 dimethyladenosine transferase 1-like (S. cerevisiae) | 1.62 | 0.00031 | 0.0028 |
| EHD1 | nucleotide binding | AW182860 | EH-domain containing 1 | 0.50 | 0.00031 | 0.0028 |
| IPO7 | protein import into nucleus, docking | AA939270 | importin 7 | 1.50 | 0.00032 | 0.0029 |
| IFITM3 | plasma membrane | BF338947 | interferon induced transmembrane protein 3 (1-8U) | 0.85 | 0.00032 | 0.0029 |
| PIM3 | nucleotide binding | BE778706 | pim-3 oncogene | 0.78 | 0.00032 | 0.0029 |
| MARK2 | nucleotide binding | NM_017490 | MAP/microtubule affinity-regulating kinase 2 | 0.62 | 0.00032 | 0.0029 |
| GPD2 | glycerol-3-phosphate dehydrogenase activity | AA613031 | glycerol-3-phosphate dehydrogenase 2 (mitochondrial) | 1.55 | 0.00032 | 0.0029 |
| EIF4A2 | nucleotide binding | NM_001967 | eukaryotic translation initiation factor 4A, isoform 2 | 1.66 | 0.00032 | 0.0029 |
| SLC23A2 | transporter activity | AL389886 | solute carrier family 23 (nucleobase transporters), member 2 | 1.45 | 0.00033 | 0.0029 |
| TERF2IP | nuclear chromosome | NM_018975 | telomeric repeat binding factor 2, interacting protein | 1.30 | 0.00033 | 0.0030 |
| PLEKHO1 | membrane fraction | NM_016274 | pleckstrin homology domain containing, family O member 1 | 0.82 | 0.00034 | 0.0030 |
| MARCO | scavenger receptor activity | NM_006770 | macrophage receptor with collagenous structure | 0.57 | 0.00034 | 0.0030 |
| ANK1 | structural constituent of cytoskeleton | NM_020479 | ankyrin 1, erythrocytic | 0.45 | 0.00034 | 0.0030 |
| PPP1R7 | protein binding | NM_002712 | protein phosphatase 1, regulatory (inhibitor) subunit 7 | 0.86 | 0.00034 | 0.0030 |
| CTSC | cysteine-type endopeptidase activity | NM_001814 | cathepsin C | 1.53 | 0.00034 | 0.0030 |
| RSBN1 | membrane fraction | AW027347 | round spermatid basic protein 1 | 1.99 | 0.00034 | 0.0030 |
| ACBD5 | acyl-CoA binding | AL047650 | acyl-Coenzyme A binding domain containing 5 | 1.85 | 0.00034 | 0.0030 |
| FYB | protein binding | BF679849 | FYN binding protein (FYB-120/130) | 1.32 | 0.00035 | 0.0030 |
| RBM27 | nucleotide binding | AB037732 | RNA binding motif protein 27 | 2.24 | 0.00035 | 0.0031 |
| DCP1A | nuclear-transcribed mRNA catabolic process, nonsense-mediated decay | AK025344 | DCP1 decapping enzyme homolog A (S. cerevisiae) | 1.39 | 0.00035 | 0.0031 |
| MED28 | actin binding | BF064162 | Mediator complex subunit 28 | 1.32 | 0.00036 | 0.0031 |
| ARAF | nucleotide binding | NM_001654 | v-raf murine sarcoma 3611 viral oncogene homolog | 0.63 | 0.00036 | 0.0031 |
| AGPAT2 | 1-acylglycerol-3-phosphate O-acyltransferase activity | U56418 | 1-acylglycerol-3-phosphate O-acyltransferase 2 (lysophosphatidic acid acyltransferase, beta) | 0.71 | 0.00036 | 0.0032 |
| ZDHHC17 | Golgi membrane | AI621223 | zinc finger, DHHC-type containing 17 | 1.88 | 0.00038 | 0.0033 |
| ACP1 | acid phosphatase activity | NM_004300 | acid phosphatase 1, soluble | 1.49 | 0.00038 | 0.0033 |
| CUL4A | G1/S transition of mitotic cell cycle | AL037208 | cullin 4A | 1.54 | 0.00038 | 0.0033 |
| TMEM123 | receptor activity | BG538627 | transmembrane protein 123 | 2.56 | 0.00038 | 0.0033 |
| SAR1B | nucleotide binding | NM_016103 | SAR1 gene homolog B (S. cerevisiae) | 1.51 | 0.00038 | 0.0033 |
| FGL2 | receptor binding | AW135176 | fibrinogen-like 2 | 2.78 | 0.00039 | 0.0033 |
| MED13 | mediator complex | AI984051 | mediator complex subunit 13 | 1.33 | 0.00040 | 0.0034 |
| SFPQ | nucleotide binding | NM_005066 | splicing factor proline/glutamine-rich (polypyrimidine tract binding protein associated) | 1.61 | 0.00040 | 0.0034 |
| AMD1 | adenosylmethionine decarboxylase activity | M21154 | adenosylmethionine decarboxylase 1 | 1.48 | 0.00040 | 0.0034 |
| ZNF319 | DNA binding | BG260623 | zinc finger protein 319 | 0.72 | 0.00040 | 0.0034 |
| ERICH1 | --- | BE644894 | glutamate-rich 1 | 1.31 | 0.00041 | 0.0034 |
| CD59 | protein binding | NM_000611 | CD59 molecule, complement regulatory protein | 0.81 | 0.00041 | 0.0034 |
| ABI3 | protein binding | AL136709 | ABI gene family, member 3 | 0.69 | 0.00041 | 0.0034 |
| FRYL | binding | AI126634 | FRY-like | 1.54 | 0.00041 | 0.0035 |
| MED23 | transcription coactivator activity | AL136776 | mediator complex subunit 23 | 1.69 | 0.00041 | 0.0035 |
| CYLD | structural constituent of ribosome | AL050166 | cylindromatosis (turban tumor syndrome) | 1.56 | 0.00041 | 0.0035 |
| IER3IP1 | endoplasmic reticulum | AL136667 | immediate early response 3 interacting protein 1 | 2.05 | 0.00042 | 0.0035 |
| SMCHD1 | protein binding | AA868754 | structural maintenance of chromosomes flexible hinge domain containing 1 | 1.64 | 0.00042 | 0.0035 |
| WTAP | membrane fraction | NM_004906 | Wilms tumor 1 associated protein | 1.67 | 0.00042 | 0.0035 |
| MED8 | mediator complex | BG230758 | mediator complex subunit 8 | 1.29 | 0.00043 | 0.0035 |
| PTPN23 | protein tyrosine phosphatase activity | AF290614 | protein tyrosine phosphatase, non-receptor type 23 | 0.73 | 0.00043 | 0.0035 |
| ACSL5 | magnesium ion binding | NM_016234 | acyl-CoA synthetase long-chain family member 5 | 1.30 | 0.00044 | 0.0036 |
| SLC25A24 | binding | NM_013386 | solute carrier family 25 (mitochondrial carrier; phosphate carrier), member 24 | 2.42 | 0.00044 | 0.0036 |
| NME6 | nucleotide binding | BC001808 | non-metastatic cells 6, protein expressed in (nucleoside-diphosphate kinase) | 0.85 | 0.00044 | 0.0036 |
| GOLPH3L | --- | NM_018178 | golgi phosphoprotein 3-like | 1.36 | 0.00045 | 0.0037 |
| MRPL9 | structural constituent of ribosome | BC004517 | mitochondrial ribosomal protein L9 | 1.30 | 0.00045 | 0.0037 |
| MALAT1 | --- | NM_014086 | metastasis associated lung adenocarcinoma transcript 1 (non-protein coding) | 3.25 | 0.00045 | 0.0037 |
| CAMTA2 | protein binding | AB020716 | calmodulin binding transcription activator 2 | 0.75 | 0.00046 | 0.0037 |
| SELT | selenocysteine incorporation | BF692332 | selenoprotein T | 1.71 | 0.00046 | 0.0037 |
| ZMPSTE24 | Golgi membrane | NM_005857 | zinc metallopeptidase (STE24 homolog, S. cerevisiae) | 1.76 | 0.00046 | 0.0037 |
| NOL7 | membrane fraction | AF130102 | nucleolar protein 7, 27kDa | 1.33 | 0.00047 | 0.0038 |
| TMEM32 | endoplasmic reticulum | BF978280 | transmembrane protein 32 | 1.40 | 0.00047 | 0.0038 |
| F11R | thiosulfate sulfurtransferase activity | AF172398 | F11 receptor | 0.71 | 0.00047 | 0.0038 |
| NUCB1 | DNA binding | BC002356 | nucleobindin 1 | 0.38 | 0.00047 | 0.0038 |
| LIMS1 | neurotransmitter secretion | NM_004987 | LIM and senescent cell antigen-like domains 1 | 1.47 | 0.00048 | 0.0038 |
| JMJD2B | nucleic acid binding | BE256900 | jumonji domain containing 2B | 0.70 | 0.00048 | 0.0039 |
| MAD2L2 | protein binding | AF080398 | MAD2 mitotic arrest deficient-like 2 (yeast) | 0.75 | 0.00048 | 0.0039 |
| EXOC1 | exocyst | AK023461 | exocyst complex component 1 | 1.64 | 0.00049 | 0.0039 |
| AGL | 4-alpha-glucanotransferase activity | NM_000645 | amylo-1, 6-glucosidase, 4-alpha-glucanotransferase | 2.67 | 0.00049 | 0.0039 |
| RPRD1A | --- | AI684281 | regulation of nuclear pre-mRNA domain containing 1A | 1.51 | 0.00050 | 0.0039 |
| GNPAT | peroxisome | NM_014236 | glyceronephosphate O-acyltransferase | 1.51 | 0.00050 | 0.0039 |
| CDC42SE2 | structural molecule activity | AF131831 | CDC42 small effector 2 | 1.57 | 0.00050 | 0.0040 |
| SLC15A3 | transporter activity | NM_016582 | solute carrier family 15, member 3 | 0.59 | 0.00051 | 0.0040 |
| DHX36 | nucleotide binding | AF217190 | DEAH (Asp-Glu-Ala-His) box polypeptide 36 | 1.60 | 0.00051 | 0.0040 |
| SAPS2 | --- | NM_014678 | SAPS domain family, member 2 | 0.75 | 0.00051 | 0.0040 |
| RBBP5 | protein binding | NM_005057 | retinoblastoma binding protein 5 | 0.68 | 0.00052 | 0.0040 |
| HK3 | nucleotide binding | NM_002115 | hexokinase 3 (white cell) | 0.73 | 0.00052 | 0.0040 |
| SETX | nucleotide binding | N64643 | senataxin | 1.43 | 0.00052 | 0.0040 |
| CDC73 | protein binding | NM_024529 | cell division cycle 73, Paf1/RNA polymerase II complex component, homolog (S. cerevisiae) | 1.94 | 0.00052 | 0.0040 |
| MAPK1 | MAPKKK cascade | AA129773 | mitogen-activated protein kinase 1 | 1.67 | 0.00052 | 0.0040 |
| SH3BP2 | SH3/SH2 adaptor activity | AB000463 | SH3-domain binding protein 2 | 0.66 | 0.00052 | 0.0041 |
| ITM2A | membrane | NM_004867 | integral membrane protein 2A | 1.39 | 0.00052 | 0.0041 |
| MKI67IP | nucleotide binding | AL542544 | MKI67 (FHA domain) interacting nucleolar phosphoprotein | 1.40 | 0.00054 | 0.0041 |
| MLL3 | DNA binding | BE962679 | myeloid/lymphoid or mixed-lineage leukemia 3 | 1.47 | 0.00055 | 0.0042 |
| NECAP1 | protein binding | BC002888 | NECAP endocytosis associated 1 | 1.68 | 0.00055 | 0.0042 |
| ROCK1 | Golgi membrane | AV683882 | Rho-associated, coiled-coil containing protein kinase 1 | 1.65 | 0.00055 | 0.0042 |
| ZNF207 | transcription factor activity | BE871379 | zinc finger protein 207 | 1.32 | 0.00055 | 0.0042 |
| IDI1 | magnesium ion binding | NM_004508 | isopentenyl-diphosphate delta isomerase 1 | 1.46 | 0.00056 | 0.0043 |
| PDE6D | 3',5'-cyclic-nucleotide phosphodiesterase activity | NM_002601 | phosphodiesterase 6D, cGMP-specific, rod, delta | 1.40 | 0.00056 | 0.0043 |
| PCGF5 | protein binding | AI798098 | polycomb group ring finger 5 | 2.03 | 0.00056 | 0.0043 |
| ANPEP | angiogenesis | NM_001150 | DNA-damage-inducible transcript 4 | 0.67 | 0.00056 | 0.0043 |
| HBQ1 | oxygen transporter activity | NM_005331 | hemoglobin, theta 1 | 0.29 | 0.00058 | 0.0044 |
| CUGBP2 | nucleotide binding | NM_006561 | CUG triplet repeat, RNA binding protein 2 | 1.52 | 0.00058 | 0.0044 |
| SMARCA5 | nucleotide binding | AV712064 | SWI/SNF related, matrix associated, actin dependent regulator of chromatin, subfamily a, member 5 | 1.97 | 0.00058 | 0.0044 |
| GATA3 | cell fate determination | BC003070 | GATA binding protein 3 | 1.42 | 0.00058 | 0.0044 |
| RAB14 | nucleotide binding | AA919115 | RAB14, member RAS oncogene family | 1.66 | 0.00059 | 0.0044 |
| TLE4 | molecular_function | NM_007005 | transducin-like enhancer of split 4 (E(sp1) homolog, Drosophila) | 1.46 | 0.00060 | 0.0045 |
| RNASEH2C | membrane fraction | AI990526 | ribonuclease H2, subunit C | 0.79 | 0.00060 | 0.0045 |
| ALPK1 | protein serine/threonine kinase activity | AI760166 | alpha-kinase 1 | 0.76 | 0.00061 | 0.0045 |
| CD47 | protein binding | BG230614 | CD47 molecule | 1.42 | 0.00061 | 0.0046 |
| NDRG1 | protein binding | NM_006096 | N-myc downstream regulated gene 1 | 0.86 | 0.00062 | 0.0046 |
| TLN1 | ruffle | NM_006289 | talin 1 | 0.54 | 0.00062 | 0.0046 |
| CRBN | ATP-dependent peptidase activity | AI676103 | cereblon | 1.64 | 0.00062 | 0.0046 |
| ALAD | porphobilinogen synthase activity | BC000977 | aminolevulinate, delta-, dehydratase | 0.75 | 0.00063 | 0.0046 |
| CAMK2G | nucleotide binding | AI093569 | calcium/calmodulin-dependent protein kinase (CaM kinase) II gamma | 0.78 | 0.00063 | 0.0047 |
| SMAD2 | mesoderm formation | AW151617 | SMAD family member 2 | 1.39 | 0.00065 | 0.0048 |
| METTL3 | RNA methylation | AW069290 | methyltransferase like 3 | 1.40 | 0.00065 | 0.0048 |
| HNRPDL | nucleotide binding | D89092 | heterogeneous nuclear ribonucleoprotein D-like | 1.69 | 0.00066 | 0.0048 |
| ST3GAL2 | beta-galactoside alpha-2,3-sialyltransferase activity | NM_006927 | ST3 beta-galactoside alpha-2,3-sialyltransferase 2 | 0.60 | 0.00067 | 0.0049 |
| MECP2 | negative regulation of transcription from RNA polymerase II promoter | L37298 | methyl CpG binding protein 2 (Rett syndrome) | 0.79 | 0.00068 | 0.0049 |
| ANKZF1 | nucleic acid binding | NM_018089 | ankyrin repeat and zinc finger domain containing 1 | 0.85 | 0.00069 | 0.0050 |
| PLOD1 | response to hypoxia | NM_000302 | procollagen-lysine 1, 2-oxoglutarate 5-dioxygenase 1 | 0.76 | 0.00070 | 0.0051 |
| IFT52 | neural tube formation | NM_016004 | intraflagellar transport 52 homolog (Chlamydomonas) | 1.35 | 0.00070 | 0.0051 |
| RNF145 | protein binding | AL553942 | ring finger protein 145 | 1.43 | 0.00070 | 0.0051 |
| HNRNPF | --- | AI591354 | heterogeneous nuclear ribonucleoprotein F | 1.54 | 0.00071 | 0.0051 |
| TXNDC9 | molecular_function | NM_005783 | thioredoxin domain containing 9 | 1.95 | 0.00071 | 0.0051 |
| ITGAX | magnesium ion binding | M81695 | integrin, alpha X (complement component 3 receptor 4 subunit) | 0.72 | 0.00072 | 0.0052 |
| GOLGA2 | protein binding | L06147 | golgi autoantigen, golgin subfamily a, 2 | 0.63 | 0.00072 | 0.0052 |
| DMTF1 | transcription factor activity | NM_021145 | cyclin D binding myb-like transcription factor 1 | 1.59 | 0.00072 | 0.0052 |
| CFL1 | actin binding | D00682 | cofilin 1 (non-muscle) | 0.80 | 0.00073 | 0.0052 |
| FCGRT | receptor activity | NM_004107 | Fc fragment of IgG, receptor, transporter, alpha | 0.77 | 0.00073 | 0.0052 |
| PPP2R5B | protein phosphatase type 2A complex | NM_006244 | protein phosphatase 2, regulatory subunit B', beta isoform | 0.68 | 0.00073 | 0.0052 |
| EDF1 | transcription coactivator activity | AB002282 | endothelial differentiation-related factor 1 | 0.82 | 0.00073 | 0.0052 |
| RNF125 | protein binding | AI969697 | ring finger protein 125 | 1.66 | 0.00073 | 0.0052 |
| LONP2 | nucleotide binding | AI971258 | Lon peptidase 2, peroxisomal | 1.50 | 0.00074 | 0.0052 |
| CGGBP1 | DNA binding | BE501318 | CGG triplet repeat binding protein 1 | 1.94 | 0.00074 | 0.0052 |
| SR140 | nucleotide binding | AI184562 | U2-associated SR140 protein | 1.61 | 0.00074 | 0.0053 |
| RBL2 | DNA binding | BF110947 | retinoblastoma-like 2 (p130) | 1.43 | 0.00075 | 0.0053 |
| YWHAQ | cytoplasm | BF033313 | tyrosine 3-monooxygenase/tryptophan 5-monooxygenase activation protein, theta polypeptide | 1.92 | 0.00075 | 0.0053 |
| CLK1 | nucleotide binding | AI251890 | CDC-like kinase 1 | 1.70 | 0.00077 | 0.0054 |
| MYBL1 | DNA binding | AW592266 | v-myb myeloblastosis viral oncogene homolog (avian)-like 1 | 2.03 | 0.00077 | 0.0054 |
| OSTM1 | membrane | NM_014028 | osteopetrosis associated transmembrane protein 1 | 2.01 | 0.00077 | 0.0054 |
| KTELC1 | endoplasmic reticulum | AV682567 | KTEL (Lys-Tyr-Glu-Leu) containing 1 | 1.49 | 0.00078 | 0.0055 |
| VASP | actin binding | NM_003370 | vasodilator-stimulated phosphoprotein | 0.73 | 0.00079 | 0.0055 |
| ARMC10 | regulation of cell growth | BC003586 | armadillo repeat containing 10 | 1.35 | 0.00079 | 0.0055 |
| SUSD3 | membrane | AW966474 | sushi domain containing 3 | 1.34 | 0.00079 | 0.0055 |
| ZNF740 | nucleic acid binding | BF114738 | zinc finger protein 740 | 0.82 | 0.00079 | 0.0055 |
| USP32 | ubiquitin thiolesterase activity | AI148567 | ubiquitin specific peptidase 32 | 2.58 | 0.00079 | 0.0055 |
| MRFAP1L1 | ribosome | AL566962 | Morf4 family associated protein 1-like 1 | 1.49 | 0.00080 | 0.0055 |
| PRRC1 | Golgi apparatus | AL524045 | proline-rich coiled-coil 1 | 1.57 | 0.00081 | 0.0056 |
| SLC30A7 | cation transport | AI761597 | solute carrier family 30 (zinc transporter), member 7 | 1.49 | 0.00082 | 0.0057 |
| MS4A7 | receptor activity | AB026043 | membrane-spanning 4-domains, subfamily A, member 7 | 1.41 | 0.00082 | 0.0057 |
| AIDA | --- | W72220 | axin interactor, dorsalization associated | 1.62 | 0.00083 | 0.0057 |
| ANGEL2 | --- | AU151793 | angel homolog 2 (Drosophila) | 1.38 | 0.00084 | 0.0057 |
| TRAM1 | receptor activity | NM_014294 | translocation associated membrane protein 1 | 1.53 | 0.00084 | 0.0057 |
| AGPAT3 | 1-acylglycerol-3-phosphate O-acyltransferase activity | BE737251 | 1-acylglycerol-3-phosphate O-acyltransferase 3 | 0.84 | 0.00084 | 0.0057 |
| PPM1A | magnesium ion binding | AF070670 | protein phosphatase 1A (formerly 2C), magnesium-dependent, alpha isoform | 1.91 | 0.00085 | 0.0058 |
| AP2S1 | transporter activity | BC006337 | adaptor-related protein complex 2, sigma 1 subunit | 0.66 | 0.00086 | 0.0058 |
| SLC15A4 | transporter activity | AI636759 | solute carrier family 15, member 4 | 1.42 | 0.00086 | 0.0058 |
| VPS13C | protein localization | NM_017684 | vacuolar protein sorting 13 homolog C (S. cerevisiae) | 1.76 | 0.00086 | 0.0058 |
| SPATA2L | --- | AF070574 | spermatogenesis associated 2-like | 0.66 | 0.00086 | 0.0058 |
| MTO1 | mitochondrion | AK026073 | mitochondrial translation optimization 1 homolog (S. cerevisiae) | 1.62 | 0.00086 | 0.0058 |
| ATP2B4 | nucleotide binding | AW517686 | ATPase, Ca++ transporting, plasma membrane 4 | 0.75 | 0.00087 | 0.0058 |
| IGHMBP2 | nucleotide binding | L14754 | immunoglobulin mu binding protein 2 | 0.86 | 0.00087 | 0.0058 |
| CBFB | transcription factor activity | NM_001755 | core-binding factor, beta subunit | 1.76 | 0.00087 | 0.0058 |
| ZZZ3 | DNA binding | AL080063 | zinc finger, ZZ-type containing 3 | 1.75 | 0.00087 | 0.0058 |
| RBM39 | nucleotide binding | NM_004902 | RNA binding motif protein 39 | 1.80 | 0.00089 | 0.0060 |
| ASF1A | chromatin binding | AB028628 | ASF1 anti-silencing function 1 homolog A (S. cerevisiae) | 1.63 | 0.00090 | 0.0060 |
| ENTPD4 | magnesium ion binding | AB002390 | ectonucleoside triphosphate diphosphohydrolase 4 | 1.32 | 0.00090 | 0.0060 |
| SLC16A6 | transporter activity | NM_004694 | solute carrier family 16, member 6 (monocarboxylic acid transporter 7) | 1.31 | 0.00090 | 0.0060 |
| MEF2C | regulation of cell growth | AL536517 | myocyte enhancer factor 2C | 1.47 | 0.00090 | 0.0060 |
| RCHY1 | protein binding | AA524525 | ring finger and CHY zinc finger domain containing 1 | 2.13 | 0.00091 | 0.0060 |
| P2RY14 | rhodopsin-like receptor activity | NM_014879 | purinergic receptor P2Y, G-protein coupled, 14 | 1.63 | 0.00091 | 0.0060 |
| EML4 | molecular_function | AV707345 | echinoderm microtubule associated protein like 4 | 1.32 | 0.00091 | 0.0060 |
| MTDH | membrane fraction | AV700332 | metadherin | 2.25 | 0.00091 | 0.0060 |
| NIPBL | binding | NM_015384 | Nipped-B homolog (Drosophila) | 1.41 | 0.00092 | 0.0060 |
| YPEL5 | --- | NM_016061 | yippee-like 5 (Drosophila) | 1.81 | 0.00092 | 0.0061 |
| RICTOR | binding | AI743511 | rapamycin-insensitive companion of mTOR | 1.74 | 0.00092 | 0.0061 |
| ZNF443 | DNA binding | NM_005815 | zinc finger protein 443 | 1.36 | 0.00093 | 0.0061 |
| SRP72 | binding | BE856385 | signal recognition particle 72kDa | 1.63 | 0.00093 | 0.0061 |
| BACH1 | transcription factor activity | NM_001186 | BTB and CNC homology 1, basic leucine zipper transcription factor 1 | 2.65 | 0.00094 | 0.0061 |
| AKAP8L | DNA binding | AB015332 | A kinase (PRKA) anchor protein 8-like | 0.75 | 0.00094 | 0.0061 |
| PDE8A | two-component response regulator activity | BE568219 | phosphodiesterase 8A | 1.35 | 0.00094 | 0.0061 |
| CCNL1 | membrane fraction | NM_020307 | cyclin L1 | 1.55 | 0.00094 | 0.0061 |
| TMED7 | regulation of cytokine production | AF151867 | transmembrane emp24 protein transport domain containing 7 | 1.72 | 0.00095 | 0.0061 |
| TFE3 | nucleotide binding | AL161985 | transcription factor binding to IGHM enhancer 3 | 0.65 | 0.00096 | 0.0062 |
| TMBIM4 | protein binding | NM_016056 | transmembrane BAX inhibitor motif containing 4 | 0.81 | 0.00097 | 0.0063 |
| SFRS2 | nucleotide binding | BG254869 | splicing factor, arginine/serine-rich 2 | 1.63 | 0.00098 | 0.0063 |
| ME2 | malate dehydrogenase (decarboxylating) activity | M55905 | malic enzyme 2, NAD(+)-dependent, mitochondrial | 1.48 | 0.00099 | 0.0063 |
| CASP8 | signal transducer activity | BF439983 | caspase 8, apoptosis-related cysteine peptidase | 0.78 | 0.0010 | 0.0065 |
| PRAM1 | integrin-mediated signaling pathway | AW027174 | PML-RARA regulated adaptor molecule 1 | 0.75 | 0.0010 | 0.0065 |
| TBRG1 | DNA binding | AI701055 | transforming growth factor beta regulator 1 | 1.33 | 0.0010 | 0.0065 |
| TES | zinc ion binding | NM_015641 | testis derived transcript (3 LIM domains) | 1.30 | 0.0010 | 0.0066 |
| DNM2 | G2/M transition of mitotic cell cycle | NM_004945 | dynamin 2 | 0.68 | 0.0011 | 0.0067 |
| NGLY1 | peptide-N4-(N-acetyl-beta-glucosaminyl)asparagine amidase activity | NM_018297 | N-glycanase 1 | 1.40 | 0.0011 | 0.0067 |
| GFM1 | nucleotide binding | AI814295 | G elongation factor, mitochondrial 1 | 1.95 | 0.0011 | 0.0068 |
| FEM1B | receptor activity | AI799061 | fem-1 homolog b (C. elegans) | 1.40 | 0.0011 | 0.0068 |
| PIM1 | nucleotide binding | M24779 | pim-1 oncogene | 0.79 | 0.0011 | 0.0069 |
| TOMM70A | protein binding | NM_014820 | translocase of outer mitochondrial membrane 70 homolog A (S. cerevisiae) | 1.37 | 0.0011 | 0.0069 |
| TLE2 | protein binding | M99436 | transducin-like enhancer of split 2 (E(sp1) homolog, Drosophila) | 1.39 | 0.0011 | 0.0069 |
| PPIL3 | peptidyl-prolyl cis-trans isomerase activity | AF251049 | peptidylprolyl isomerase (cyclophilin)-like 3 | 1.74 | 0.0011 | 0.0069 |
| ZNF238 | negative regulation of transcription from RNA polymerase II promoter | AJ223321 | zinc finger protein 238 | 1.65 | 0.0011 | 0.0069 |
| SLC35B3 | Golgi membrane | AL355815 | solute carrier family 35, member B3 | 1.79 | 0.0011 | 0.0070 |
| TINP1 | intracellular | NM_014886 | TGF beta-inducible nuclear protein 1 | 1.43 | 0.0011 | 0.0070 |
| EIF3H | translation initiation factor activity | NM_003756 | eukaryotic translation initiation factor 3, subunit H | 1.29 | 0.0011 | 0.0070 |
| IQCB1 | calmodulin binding | NM_014642 | IQ motif containing B1 | 1.30 | 0.0011 | 0.0070 |
| GTF2A2 | transcription factor activity | NM_004492 | general transcription factor IIA, 2, 12kDa | 1.31 | 0.0011 | 0.0070 |
| RASGRP4 | guanyl-nucleotide exchange factor activity | AA923524 | RAS guanyl releasing protein 4 | 0.62 | 0.0011 | 0.0070 |
| DDX3X | nucleotide binding | NM_001356 | DEAD (Asp-Glu-Ala-Asp) box polypeptide 3, X-linked | 1.47 | 0.0011 | 0.0071 |
| MTF1 | transcription factor activity | N46867 | metal-regulatory transcription factor 1 | 1.32 | 0.0011 | 0.0071 |
| NT5C2 | nucleotide binding | BC001595 | 5'-nucleotidase, cytosolic II | 0.82 | 0.0011 | 0.0071 |
| NR2C2 | transcription factor activity | AL138444 | nuclear receptor subfamily 2, group C, member 2 | 1.36 | 0.0011 | 0.0071 |
| TBC1D23 | Rab GTPase activator activity | AK001750 | TBC1 domain family, member 23 | 1.93 | 0.0012 | 0.0071 |
| ATM | DNA binding | U82828 | ataxia telangiectasia mutated | 1.66 | 0.0012 | 0.0071 |
| LMNA | structural molecule activity | AA063189 | Lamin A/C | 0.71 | 0.0012 | 0.0071 |
| PPP1R12A | signal transducer activity | NM_002480 | protein phosphatase 1, regulatory (inhibitor) subunit 12A | 2.64 | 0.0012 | 0.0072 |
| SNX5 | protein binding | NM_014426 | sorting nexin 5 | 1.54 | 0.0012 | 0.0072 |
| WAS | small GTPase regulator activity | NM_000377 | Wiskott-Aldrich syndrome (eczema-thrombocytopenia) | 0.57 | 0.0012 | 0.0072 |
| VPS36 | membrane fraction | AL576924 | vacuolar protein sorting 36 homolog (S. cerevisiae) | 1.94 | 0.0012 | 0.0072 |
| SOCS2 | regulation of cell growth | NM_003877 | suppressor of cytokine signaling 2 | 1.71 | 0.0012 | 0.0072 |
| AP3M1 | protein binding | AA700485 | adaptor-related protein complex 3, mu 1 subunit | 1.36 | 0.0012 | 0.0073 |
| TCEA1 | DNA binding | X57198 | transcription elongation factor A (SII), 1 | 1.65 | 0.0012 | 0.0073 |
| UTRN | actin binding | N66570 | utrophin | 1.57 | 0.0012 | 0.0073 |
| ATP6V0A1 | protein binding | AL096733 | ATPase, H+ transporting, lysosomal V0 subunit a1 | 0.61 | 0.0012 | 0.0074 |
| PBXIP1 | transcription corepressor activity | AI935162 | pre-B-cell leukemia homeobox interacting protein 1 | 0.83 | 0.0012 | 0.0074 |
| KPNB1 | protein import into nucleus, docking | BG249565 | karyopherin (importin) beta 1 | 0.63 | 0.0012 | 0.0074 |
| GYPC | protein binding | NM_002101 | glycophorin C (Gerbich blood group) | 0.64 | 0.0013 | 0.0076 |
| ARPC5 | actin binding | AL516350 | actin related protein 2/3 complex, subunit 5, 16kDa | 1.73 | 0.0013 | 0.0077 |
| RBMS1 | DNA binding | AI580100 | RNA binding motif, single stranded interacting protein 1 | 1.42 | 0.0013 | 0.0077 |
| EIF5 | nucleotide binding | AK026933 | eukaryotic translation initiation factor 5 | 1.80 | 0.0013 | 0.0079 |
| STK11 | nucleotide binding | AI914604 | serine/threonine kinase 11 | 0.77 | 0.0013 | 0.0080 |
| FARSA | nucleotide binding | AD000092 | phenylalanyl-tRNA synthetase, alpha subunit | 0.83 | 0.0014 | 0.0081 |
| HIF1AN | peptide-aspartate beta-dioxygenase activity | NM_017902 | hypoxia-inducible factor 1, alpha subunit inhibitor | 0.80 | 0.0014 | 0.0081 |
| TUG1 | --- | AK000749 | taurine upregulated gene 1 | 1.59 | 0.0014 | 0.0081 |
| SFRS7 | nucleotide binding | AA524053 | splicing factor, arginine/serine-rich 7, 35kDa | 1.73 | 0.0014 | 0.0082 |
| MSL3L1 | chromatin | NM_006800 | male-specific lethal 3-like 1 (Drosophila) | 1.29 | 0.0014 | 0.0083 |
| ABR | guanyl-nucleotide exchange factor activity | L19704 | active BCR-related gene | 0.71 | 0.0014 | 0.0083 |
| CENTA2 | phosphatidylinositol-4,5-bisphosphate binding | NM_018404 | centaurin, alpha 2 | 0.69 | 0.0014 | 0.0083 |
| TACC1 | protein binding | AB029026 | transforming, acidic coiled-coil containing protein 1 | 0.65 | 0.0014 | 0.0084 |
| EIF1AY | RNA binding | NM_004681 | eukaryotic translation initiation factor 1A, Y-linked | 1.82 | 0.0015 | 0.0086 |
| IDE | insulysin activity | N22903 | insulin-degrading enzyme | 1.41 | 0.0015 | 0.0087 |
| UBA1 | --- | NM_003334 | ubiquitin-like modifier activating enzyme 1 | 0.77 | 0.0015 | 0.0087 |
| ARL4C | nucleotide binding | BG435404 | ADP-ribosylation factor-like 4C | 1.33 | 0.0015 | 0.0087 |
| ENOSF1 | catalytic activity | NM_017512 | enolase superfamily member 1 | 1.42 | 0.0015 | 0.0088 |
| PPP3CC | phosphoprotein phosphatase activity | NM_005605 | protein phosphatase 3 (formerly 2B), catalytic subunit, gamma isoform | 1.44 | 0.0015 | 0.0088 |
| FBXO18 | nucleotide binding | BE961916 | F-box protein, helicase, 18 | 0.78 | 0.0015 | 0.0090 |
| NUDT21 | RNA binding | AL520677 | nudix (nucleoside diphosphate linked moiety X)-type motif 21 | 2.27 | 0.0015 | 0.0090 |
| MORN1 | --- | NM_024848 | MORN repeat containing 1 | 0.77 | 0.0016 | 0.0091 |
| OGFR | regulation of cell growth | NM_007346 | TAF15 RNA polymerase II, TATA box binding protein (TBP)-associated factor, 68kDa | 0.61 | 0.0016 | 0.0091 |
| ATP6V1C1 | transporter activity | AW024925 | TNF receptor-associated factor 4 | 1.43 | 0.0016 | 0.0092 |
| TRIM37 | protein binding | AK022701 | tripartite motif-containing 37 | 1.39 | 0.0016 | 0.0092 |
| LRG1 | molecular_function | AA622495 | leucine-rich alpha-2-glycoprotein 1 | 0.61 | 0.0016 | 0.0093 |
| SPG11 | membrane fraction | NM_025137 | spastic paraplegia 11 (autosomal recessive) | 1.32 | 0.0016 | 0.0093 |
| ATP5I | mitochondrion | NM_007100 | ATP synthase, H+ transporting, mitochondrial F0 complex, subunit E | 0.63 | 0.0016 | 0.0093 |
| RALGPS2 | guanyl-nucleotide exchange factor activity | AW003297 | Ral GEF with PH domain and SH3 binding motif 2 | 1.38 | 0.0016 | 0.0093 |
| N4BP2L1 | --- | N80918 | NEDD4 binding protein 2-like 1 | 1.78 | 0.0016 | 0.0093 |
| UNC93B1 | membrane | AW001274 | unc-93 homolog B1 (C. elegans) | 0.57 | 0.0016 | 0.0093 |
| HPS3 | cytoplasm | AA219354 | Hermansky-Pudlak syndrome 3 | 1.62 | 0.0016 | 0.0093 |
| TNRC6A | nucleotide binding | N21600 | trinucleotide repeat containing 6A | 1.38 | 0.0016 | 0.0093 |
| ROGDI | --- | NM_024589 | rogdi homolog (Drosophila) | 0.59 | 0.0016 | 0.0093 |
| SPAST | nucleotide binding | AB029006 | spastin | 2.22 | 0.0016 | 0.0094 |
| FIS1 | mitochondrial fission | NM_016068 | fission 1 (mitochondrial outer membrane) homolog (S. cerevisiae) | 0.68 | 0.0016 | 0.0094 |
| MYBBP1A | DNA binding | NM_014520 | MYB binding protein (P160) 1a | 0.69 | 0.0017 | 0.0094 |
| CDK6 | G1 phase of mitotic cell cycle | AW274756 | cyclin-dependent kinase 6 | 1.64 | 0.0017 | 0.0094 |
| GPR18 | rhodopsin-like receptor activity | AF261135 | G protein-coupled receptor 18 | 1.37 | 0.0017 | 0.0094 |
| SSR1 | signal sequence binding | AW006345 | signal sequence receptor, alpha | 1.69 | 0.0017 | 0.0095 |
| RFXANK | transcription factor activity | NM_003721 | regulatory factor X-associated ankyrin-containing protein | 0.80 | 0.0017 | 0.0095 |
| GMIP | GTPase activator activity | BF000144 | GEM interacting protein | 0.51 | 0.0017 | 0.0096 |
| GIMAP6 | GTP binding | NM_024711 | GTPase, IMAP family member 6 | 1.76 | 0.0017 | 0.0097 |
| ZNF512 | DNA binding | NM_032434 | zinc finger protein 512 | 1.35 | 0.0017 | 0.0097 |
| PTAR1 | protein prenyltransferase activity | BF056048 | protein prenyltransferase alpha subunit repeat containing 1 | 2.00 | 0.0017 | 0.0097 |
| CDR2 | molecular_function | AL582414 | cerebellar degeneration-related protein 2, 62kDa | 1.34 | 0.0017 | 0.0097 |
| STARD3NL | endosome | BC003074 | STARD3 N-terminal like | 1.53 | 0.0017 | 0.0097 |
| DCUN1D5 | --- | BC004169 | DCN1, defective in cullin neddylation 1, domain containing 5 (S. cerevisiae) | 1.38 | 0.0018 | 0.0098 |
| RNASEK | --- | BE744389 | ribonuclease, RNase K | 0.73 | 0.0018 | 0.0099 |
| ZNF131 | transcription factor activity | AW968301 | zinc finger protein 131 | 1.39 | 0.0018 | 0.0099 |
|  |  |  |  |  |  |  |

^a^: Genes with P Values < 0.01 were sorted by P Values in an ascending order.

^b^: Gene ontology (GO) annotations were obtained by the GOstat program (<http://gostat.wehi.edu.au/>), and the first annotation was shown when a gene has multiple GO annotations. Missing value was presented as “---”.

^c^: FC, denoting fold change, is defined as the ratio of the expression value of positive over negative responders.

^d^: P Value was calculated using the ordinary Student’s *t* test for each gene.

^e^: FDR, denpoting false discovery rate, was estimated by the Benjamini-Hochberg (BH) method.

#### Table S2. Differentially Expressed Gene List for Week 8 Placebo Group Between Responders (*n* = 4) and Non-responders (*n* = 17)^a^

|  |  |  |  |  |  |  |
| --- | --- | --- | --- | --- | --- | --- |
| Gene Symbol | GO Annotation^b^ | GenBank  Acc. Number | Gene Name | FC^c^ | P Value^d^ | FDR^e^ |
|  |  |  |  |
|  |  |  |  |  |  |  |
|  |  |  |  |  |  |  |
| TPR | nucleotide binding | NM_003292 | translocated promoter region (to activated MET oncogene) | 1.59 | <1.00×10^-6^ | <1.00×10^-5^ |
| DDT | dopachrome isomerase activity | NM_001355 | D-dopachrome tautomerase | 0.62 | <1.00×10^-6^ | <1.00×10^-5^ |
| MED12 | mediator complex | NM_005120 | mediator complex subunit 12 | 1.67 | <1.00×10^-6^ | <1.00×10^-5^ |
| PCBP2 | DNA binding | NM_005016 | poly(rC) binding protein 2 | 1.30 | <1.00×10^-6^ | <1.00×10^-5^ |
| HIGD1A | protein binding | BE739519 | HIG1 domain family, member 1A | 0.57 | <1.00×10^-6^ | <1.00×10^-5^ |
| BCCIP | regulation of cyclin-dependent protein kinase activity | BE464077 | BRCA2 and CDKN1A interacting protein | 0.63 | <1.00×10^-6^ | <1.00×10^-5^ |
| NEDD8 | protein binding | NM_006156 | neural precursor cell expressed, developmentally down-regulated 8 | 0.75 | 1.00×10^-6^ | 0.00039 |
| PRR13 | --- | NM_018457 | proline rich 13 | 1.29 | 1.00×10^-6^ | 0.00039 |
| TMEM80 | membrane | AI739035 | transmembrane protein 80 | 0.76 | 1.00×10^-6^ | 0.00039 |
| GOT1 | L-aspartate:2-oxoglutarate aminotransferase activity | BC000498 | glutamic-oxaloacetic transaminase 1, soluble (aspartate aminotransferase 1) | 1.33 | 2.00×10^-6^ | 0.00059 |
| GCN1L1 | protein binding | AI697055 | GCN1 general control of amino-acid synthesis 1-like 1 (yeast) | 1.61 | 2.00×10^-6^ | 0.00059 |
| UBL5 | molecular_function | NM_024292 | ubiquitin-like 5 | 0.55 | 2.00×10^-6^ | 0.00059 |
| SLC25A36 | transporter activity | AL049246 | solute carrier family 25, member 36 | 0.70 | 3.00×10^-6^ | 0.00074 |
| ACTG1 | --- | AA703939 | actin, gamma 1 | 1.25 | 3.00×10^-6^ | 0.00074 |
| STX6 | Golgi membrane | NM_005819 | syntaxin 6 | 1.24 | 4.00×10^-6^ | 0.00074 |
| APLP2 | DNA binding | BC000373 | amyloid beta (A4) precursor-like protein 2 | 1.43 | 4.00×10^-6^ | 0.00074 |
| RRAGD | nucleotide binding | AF272036 | Ras-related GTP binding D | 0.72 | 4.00×10^-6^ | 0.00074 |
| DUSP16 | inactivation of MAPK activity | AB051487 | dual specificity phosphatase 16 | 1.60 | 4.00×10^-6^ | 0.00074 |
| KLHL9 | protein binding | AK022805 | kelch-like 9 (Drosophila) | 0.66 | 4.00×10^-6^ | 0.00074 |
| ATP5B | nucleotide binding | NM_001686 | ATP synthase, H+ transporting, mitochondrial F1 complex, beta polypeptide | 1.34 | 5.00×10^-6^ | 0.00074 |
| USP7 | cysteine-type endopeptidase activity | NM_003470 | ubiquitin specific peptidase 7 (herpes virus-associated) | 1.19 | 5.00×10^-6^ | 0.00074 |
| NDUFB1 | NADH dehydrogenase activity | NM_004545 | NADH dehydrogenase (ubiquinone) 1 beta subcomplex, 1, 7kDa | 0.43 | 5.00×10^-6^ | 0.00074 |
| HIVEP2 | DNA binding | AL023584 | human immunodeficiency virus type I enhancer binding protein 2 | 1.38 | 5.00×10^-6^ | 0.00074 |
| CCDC49 | --- | H79861 | coiled-coil domain containing 49 | 0.74 | 5.00×10^-6^ | 0.00074 |
| LONP2 | nucleotide binding | BC004234 | lon peptidase 2, peroxisomal | 1.63 | 6.00×10^-6^ | 0.00078 |
| LSMD1 | mRNA metabolic process | BC006407 | LSM domain containing 1 | 0.58 | 6.00×10^-6^ | 0.00078 |
| HMGB1 | chromatin | BF673940 | high-mobility group box 1 | 0.60 | 6.00×10^-6^ | 0.00078 |
| EIF4E2 | RNA cap binding | BF516289 | eukaryotic translation initiation factor 4E family member 2 | 0.74 | 7.00×10^-6^ | 0.00088 |
| WDR74 | nucleus | NM_018093 | WD repeat domain 74 | 1.34 | 8.00×10^-6^ | 0.00094 |
| DOCK10 | guanyl-nucleotide exchange factor activity | NM_017718 | dedicator of cytokinesis 10 | 2.00 | 8.00×10^-6^ | 0.00094 |
| PPP1R10 | DNA binding | NM_002714 | protein phosphatase 1, regulatory (inhibitor) subunit 10 | 1.55 | 9.00×10^-6^ | 0.00099 |
| JMJD2A | nucleic acid binding | NM_014663 | jumonji domain containing 2A | 1.33 | 9.00×10^-6^ | 0.00099 |
| RBM15B | nucleotide binding | W68720 | RNA binding motif protein 15B | 1.37 | 1.00×10^-5^ | 0.0011 |
| PUM1 | RNA binding | BG474429 | pumilio homolog 1 (Drosophila) | 1.44 | 1.10×10^-5^ | 0.0011 |
| OSBP | Golgi membrane | AI927993 | oxysterol binding protein | 1.44 | 1.10×10^-5^ | 0.0011 |
| ALCAM | receptor binding | AA156721 | activated leukocyte cell adhesion molecule | 0.57 | 1.30×10^-5^ | 0.0013 |
| RABGAP1L | Rab GTPase activator activity | BG107203 | RAB GTPase activating protein 1-like | 0.73 | 1.40×10^-5^ | 0.0013 |
| USMG5 | membrane | BG291685 | up-regulated during skeletal muscle growth 5 homolog (mouse) | 0.54 | 1.40×10^-5^ | 0.0013 |
| MRPL53 | mitochondrion | AV726817 | mitochondrial ribosomal protein L53 | 0.66 | 1.60×10^-5^ | 0.0014 |
| ALDH9A1 | aldehyde dehydrogenase (NAD) activity | NM_000696 | aldehyde dehydrogenase 9 family, member A1 | 1.38 | 1.80×10^-5^ | 0.0016 |
| SNAP29 | SNAP receptor activity | NM_004782 | synaptosomal-associated protein, 29kDa | 0.70 | 1.90×10^-5^ | 0.0016 |
| ASRGL1 | N4-(beta-N-acetylglucosaminyl)-L-asparaginase activity | NM_025080 | asparaginase like 1 | 0.74 | 2.10×10^-5^ | 0.0016 |
| TRSPAP1 | nucleotide binding | NM_017846 | tRNA selenocysteine associated protein 1 | 0.74 | 2.10×10^-5^ | 0.0016 |
| RAB8B | nucleotide binding | NM_016530 | RAB8B, member RAS oncogene family | 0.73 | 2.10×10^-5^ | 0.0016 |
| MTRF1L | translation release factor activity | AI479199 | mitochondrial translational release factor 1-like | 0.71 | 2.10×10^-5^ | 0.0016 |
| PTBP1 | nucleotide binding | BC002397 | polypyrimidine tract binding protein 1 | 1.38 | 2.20×10^-5^ | 0.0017 |
| SMARCA2 | nucleotide binding | NM_003070 | SWI/SNF related, matrix associated, actin dependent regulator of chromatin, subfamily a, member 2 | 1.81 | 2.30×10^-5^ | 0.0017 |
| PIN4 | peptidyl-prolyl cis-trans isomerase activity | BE797213 | protein (peptidylprolyl cis/trans isomerase) NIMA-interacting, 4 (parvulin) | 0.53 | 2.40×10^-5^ | 0.0018 |
| CIAO1 | protein binding | NM_004804 | cytosolic iron-sulfur protein assembly 1 homolog (S. cerevisiae) | 0.80 | 2.80×10^-5^ | 0.0020 |
| PHKA2 | catalytic activity | AL096700 | phosphorylase kinase, alpha 2 (liver) | 0.73 | 2.80×10^-5^ | 0.0020 |
| TMCC1 | membrane | AI934469 | transmembrane and coiled-coil domain family 1 | 0.74 | 2.90×10^-5^ | 0.0020 |
| NACA | DNA binding | NM_005594 | nascent polypeptide-associated complex alpha subunit | 1.15 | 3.20×10^-5^ | 0.0021 |
| ZNF143 | DNA binding | AW162015 | zinc finger protein 143 | 1.40 | 3.20×10^-5^ | 0.0021 |
| AP1GBP1 | calcium ion binding | BE856321 | AP1 gamma subunit binding protein 1 | 1.62 | 3.20×10^-5^ | 0.0021 |
| NUP93 | protein binding | NM_014669 | nucleoporin 93kDa | 1.39 | 3.50×10^-5^ | 0.0022 |
| LUC7L2 | protein binding | NM_016007 | LUC7-like 2 (S. cerevisiae) | 1.32 | 3.50×10^-5^ | 0.0022 |
| DUSP23 | protein tyrosine phosphatase activity | BC001140 | dual specificity phosphatase 23 | 0.64 | 3.60×10^-5^ | 0.0022 |
| SSR1 | signal sequence binding | BF206389 | signal sequence receptor, alpha | 1.38 | 3.60×10^-5^ | 0.0022 |
| TUBA1B | nucleotide binding | BC006379 | tubulin, alpha 1b | 1.16 | 3.80×10^-5^ | 0.0023 |
| ATP5E | mitochondrion | NM_006886 | ATP synthase, H+ transporting, mitochondrial F1 complex, epsilon subunit | 0.56 | 3.90×10^-5^ | 0.0023 |
| WHSC2 | nucleus | AF101434 | Wolf-Hirschhorn syndrome candidate 2 | 1.28 | 3.90×10^-5^ | 0.0023 |
| NDUFA2 | NADH dehydrogenase activity | BC003674 | NADH dehydrogenase (ubiquinone) 1 alpha subcomplex, 2, 8kDa | 0.69 | 4.10×10^-5^ | 0.0023 |
| FBXL14 | protein binding | BF001666 | F-box and leucine-rich repeat protein 14 | 1.32 | 4.20×10^-5^ | 0.0024 |
| CTR9 | binding | NM_014633 | Ctr9, Paf1/RNA polymerase II complex component, homolog (S. cerevisiae) | 1.54 | 4.40×10^-5^ | 0.0024 |
| NFATC3 | transcription factor activity | U85430 | nuclear factor of activated T-cells, cytoplasmic, calcineurin-dependent 3 | 1.29 | 4.40×10^-5^ | 0.0024 |
| TPM1 | actin binding | M19267 | tropomyosin 1 (alpha) | 0.63 | 4.60×10^-5^ | 0.0025 |
| FCER1A | positive regulation of type I hypersensitivity | BC005912 | Fc fragment of IgE, high affinity I, receptor for; alpha polypeptide | 0.58 | 5.10×10^-5^ | 0.0027 |
| NPIP | nucleus | NM_006985 | nuclear pore complex interacting protein | 1.42 | 5.20×10^-5^ | 0.0027 |
| ACTR2 | nucleotide binding | AA699583 | ARP2 actin-related protein 2 homolog (yeast) | 2.02 | 5.40×10^-5^ | 0.0027 |
| CRAT | carnitine O-acetyltransferase activity | NM_000755 | carnitine acetyltransferase | 0.75 | 5.40×10^-5^ | 0.0027 |
| CNOT2 | nucleus | AI123426 | CCR4-NOT transcription complex, subunit 2 | 1.25 | 5.40×10^-5^ | 0.0027 |
| MRPS14 | structural constituent of ribosome | BG254653 | mitochondrial ribosomal protein S14 | 0.75 | 6.20×10^-5^ | 0.0030 |
| ARNT | protein import into nucleus, translocation | AL042842 | aryl hydrocarbon receptor nuclear translocator | 1.45 | 6.20×10^-5^ | 0.0030 |
| PDIA4 | protein disulfide isomerase activity | BC000425 | protein disulfide isomerase family A, member 4 | 1.23 | 6.60×10^-5^ | 0.0031 |
| MAP4K4 | nucleotide binding | AL561281 | mitogen-activated protein kinase kinase kinase kinase 4 | 1.55 | 6.60×10^-5^ | 0.0031 |
| MYEOV2 | --- | AL036350 | myeloma overexpressed 2 | 0.54 | 6.60×10^-5^ | 0.0031 |
| ANKRD49 | --- | NM_017704 | ankyrin repeat domain 49 | 0.68 | 6.80×10^-5^ | 0.0031 |
| RIPK2 | nucleotide binding | AF064824 | receptor-interacting serine-threonine kinase 2 | 0.80 | 6.60×10^-5^ | 0.0031 |
| CAMLG | receptor recycling | NM_001745 | calcium modulating ligand | 0.74 | 7.00×10^-5^ | 0.0031 |
| ZXDC | nucleic acid binding | BF438203 | ZXD family zinc finger C | 1.27 | 7.20×10^-5^ | 0.0031 |
| CASP9 | protein binding | AB015653 | caspase 9, apoptosis-related cysteine peptidase | 1.39 | 7.30×10^-5^ | 0.0031 |
| RPL35A | tRNA binding | AK021571 | Ribosomal protein L35a | 1.29 | 7.30×10^-5^ | 0.0031 |
| NDUFA13 | NADH dehydrogenase activity | NM_015965 | NADH dehydrogenase (ubiquinone) 1 alpha subcomplex, 13 | 0.64 | 7.30×10^-5^ | 0.0031 |
| 1-Mar | --- | NM_017923 | membrane-associated ring finger (C3HC4) 1 | 0.63 | 7.80×10^-5^ | 0.0033 |
| PRKCQ | nucleotide binding | L01087 | protein kinase C, theta | 1.37 | 8.00×10^-5^ | 0.0033 |
| ZNF302 | DNA binding | BF508739 | zinc finger protein 302 | 0.68 | 8.00×10^-5^ | 0.0033 |
| SRP19 | cytoplasm | NM_003135 | signal recognition particle 19kDa | 0.69 | 9.60×10^-5^ | 0.0039 |
| MACF1 | hydrolase activity, hydrolyzing O-glycosyl compounds | AK023406 | microtubule-actin crosslinking factor 1 | 1.39 | 9.70×10^-5^ | 0.0039 |
| MPHOSPH8 | chromatin | BC003542 | M-phase phosphoprotein 8 | 1.25 | 9.90×10^-5^ | 0.0039 |
| ZC3H7A | nucleic acid binding | NM_014153 | zinc finger CCCH-type containing 7A | 1.25 | 0.00010 | 0.0040 |
| XPO5 | tRNA binding | BC000129 | exportin 5 | 1.37 | 0.00011 | 0.0042 |
| CCBL2 | biosynthetic process | BC000819 | cysteine conjugate-beta lyase 2 | 0.73 | 0.00011 | 0.0042 |
| COX5B | cytochrome-c oxidase activity | NM_001862 | cytochrome c oxidase subunit Vb | 0.71 | 0.00011 | 0.0042 |
| CENTA2 | phosphatidylinositol-4,5-bisphosphate binding | NM_018404 | centaurin, alpha 2 | 0.70 | 0.00011 | 0.0042 |
| SGPL1 | endoplasmic reticulum | AB033078 | sphingosine-1-phosphate lyase 1 | 1.36 | 0.00012 | 0.0043 |
| HNRNPM | --- | AF061832 | heterogeneous nuclear ribonucleoprotein M | 1.47 | 0.00013 | 0.0046 |
| RNF181 | protein binding | AF151072 | ring finger protein 181 | 0.67 | 0.00013 | 0.0046 |
| EXOSC9 | 3'-5'-exoribonuclease activity | NM_005033 | exosome component 9 | 0.78 | 0.00013 | 0.0048 |
| CKLF | extracellular region | NM_016326 | chemokine-like factor | 0.61 | 0.00014 | 0.0048 |
| ACOT8 | carboxylesterase activity | NM_005469 | acyl-CoA thioesterase 8 | 0.78 | 0.00014 | 0.0048 |
| GALNT10 | Golgi membrane | NM_024564 | UDP-N-acetyl-alpha-D-galactosamine:polypeptide N-acetylgalactosaminyltransferase 10 (GalNAc-T10) | 1.41 | 0.00014 | 0.0048 |
| CPEB4 | nucleotide binding | AA772278 | cytoplasmic polyadenylation element binding protein 4 | 1.54 | 0.00014 | 0.0048 |
| GRWD1 | nucleus | AF337808 | glutamate-rich WD repeat containing 1 | 0.68 | 0.00015 | 0.0051 |
| MTF2 | DNA binding | BG033764 | metal response element binding transcription factor 2 | 0.76 | 0.00016 | 0.0053 |
| ALKBH7 | --- | AI245026 | alkB, alkylation repair homolog 7 (E. coli) | 0.69 | 0.00016 | 0.0053 |
| ATP6V1D | ion transport | AF077614 | ATPase, H+ transporting, lysosomal 34kDa, V1 subunit D | 0.78 | 0.00016 | 0.0054 |
| SPOCK2 | calcium ion binding | NM_014767 | sparc/osteonectin, cwcv and kazal-like domains proteoglycan (testican) 2 | 1.86 | 0.00017 | 0.0057 |
| AQP3 | transporter activity | N74607 | aquaporin 3 (Gill blood group) | 1.51 | 0.00018 | 0.0058 |
| PRKAG1 | cAMP-dependent protein kinase activity | NM_002733 | protein kinase, AMP-activated, gamma 1 non-catalytic subunit | 0.82 | 0.00018 | 0.0059 |
| NLRC3 | nucleotide binding | AA694067 | NLR family, CARD domain containing 3 | 1.31 | 0.00018 | 0.0059 |
| CTSZ | cysteine-type endopeptidase activity | AF073890 | cathepsin Z | 2.40 | 0.00018 | 0.0059 |
| PRPF4B | nucleotide binding | Z25435 | PRP4 pre-mRNA processing factor 4 homolog B (yeast) | 1.35 | 0.00019 | 0.0060 |
| KLHL20 | actin binding | NM_014458 | kelch-like 20 (Drosophila) | 1.33 | 0.00020 | 0.0061 |
| CEP63 | protein binding | NM_025180 | centrosomal protein 63kDa | 0.75 | 0.00020 | 0.0061 |
| HSPA5 | nucleotide binding | AF216292 | heat shock 70kDa protein 5 (glucose-regulated protein, 78kDa) | 1.45 | 0.00020 | 0.0061 |
| YTHDC1 | nucleus | N36997 | YTH domain containing 1 | 1.27 | 0.00020 | 0.0061 |
| R3HCC1 | nucleic acid binding | AI081543 | R3H domain and coiled-coil containing 1 | 0.74 | 0.00021 | 0.0061 |
| ERGIC1 | Golgi membrane | AK000752 | endoplasmic reticulum-golgi intermediate compartment (ERGIC) 1 | 1.61 | 0.00021 | 0.0061 |
| HP1BP3 | nucleosome | BG483966 | heterochromatin protein 1, binding protein 3 | 1.21 | 0.00021 | 0.0061 |
| TMEM33 | protein binding | BE245080 | transmembrane protein 33 | 0.67 | 0.00022 | 0.0063 |
| ANXA7 | voltage-gated calcium channel activity | J04543 | annexin A7 | 1.30 | 0.00022 | 0.0063 |
| PSMD14 | proteasome complex | NM_005805 | proteasome (prosome, macropain) 26S subunit, non-ATPase, 14 | 0.74 | 0.00022 | 0.0063 |
| UXS1 | binding | NM_025076 | UDP-glucuronate decarboxylase 1 | 1.43 | 0.00022 | 0.0063 |
| ANP32A | protein binding | T67821 | Acidic (leucine-rich) nuclear phosphoprotein 32 family, member A | 0.71 | 0.00022 | 0.0063 |
| ATOX1 | copper ion transmembrane transporter activity | NM_004045 | ATX1 antioxidant protein 1 homolog (yeast) | 0.75 | 0.00022 | 0.0063 |
| SFRS14 | RNA binding | BG252853 | splicing factor, arginine/serine-rich 14 | 1.26 | 0.00024 | 0.0067 |
| VPS25 | nucleus | AL528911 | vacuolar protein sorting 25 homolog (S. cerevisiae) | 0.77 | 0.00025 | 0.0068 |
| NUP188 | protein binding | AW131863 | nucleoporin 188kDa | 1.48 | 0.00025 | 0.0070 |
| HEXB | skeletal system development | NM_000521 | hexosaminidase B (beta polypeptide) | 0.78 | 0.00027 | 0.0073 |
| MS4A6A | receptor activity | AA045175 | membrane-spanning 4-domains, subfamily A, member 6A | 0.68 | 0.00027 | 0.0073 |
| BCL11A | nucleic acid binding | AF080216 | B-cell CLL/lymphoma 11A (zinc finger protein) | 0.72 | 0.00027 | 0.0073 |
| PHF3 | molecular_function | NM_015153 | PHD finger protein 3 | 1.51 | 0.00028 | 0.0073 |
| FUCA2 | alpha-L-fucosidase activity | BC003060 | fucosidase, alpha-L- 2, plasma | 0.67 | 0.00028 | 0.0073 |
| TRAM1 | receptor activity | BC000687 | translocation associated membrane protein 1 | 0.74 | 0.00028 | 0.0073 |
| EIF2B5 | ovarian follicle development | U23028 | eukaryotic translation initiation factor 2B, subunit 5 epsilon, 82kDa | 1.29 | 0.00029 | 0.0076 |
| CYBB | voltage-gated ion channel activity | AI308863 | cytochrome b-245, beta polypeptide | 0.74 | 0.00030 | 0.0077 |
| WIPF2 | actin binding | AA676803 | WAS/WASL interacting protein family, member 2 | 1.27 | 0.00030 | 0.0077 |
| TMEM131 | membrane | D87446 | transmembrane protein 131 | 1.47 | 0.00030 | 0.0078 |
| ERICH1 | --- | AA767385 | glutamate-rich 1 | 0.69 | 0.00031 | 0.0079 |
| RNF187 | protein binding | AA085748 | ring finger protein 187 | 0.77 | 0.00031 | 0.0079 |
| NFX1 | negative regulation of transcription from RNA polymerase II promoter | AW291398 | nuclear transcription factor, X-box binding 1 | 1.40 | 0.00032 | 0.0079 |
| LARP1 | RNA binding | BC001460 | La ribonucleoprotein domain family, member 1 | 1.58 | 0.00032 | 0.0080 |
| ZNF226 | DNA binding | AK023091 | zinc finger protein 226 | 0.76 | 0.00033 | 0.0081 |
| NOTCH2NL | calcium ion binding | AW024960 | Notch homolog 2 (Drosophila) N-terminal like | 0.74 | 0.00033 | 0.0081 |
| SMARCC1 | chromatin | AA593983 | SWI/SNF related, matrix associated, actin dependent regulator of chromatin, subfamily c, member 1 | 1.21 | 0.00034 | 0.0083 |
| ARPC2 | actin binding | NM_005731 | actin related protein 2/3 complex, subunit 2, 34kDa | 0.78 | 0.00035 | 0.0083 |
| IKBKAP | DNA binding | NM_003640 | inhibitor of kappa light polypeptide gene enhancer in B-cells, kinase complex-associated protein | 1.28 | 0.00035 | 0.0084 |
| BLOC1S1 | molecular_function | NM_001487 | biogenesis of lysosomal organelles complex-1, subunit 1 | 0.61 | 0.00036 | 0.0084 |
| ATAD3B | nucleotide binding | BC002542 | ATPase family, AAA domain containing 3B | 1.24 | 0.00036 | 0.0084 |
| CCDC82 | protein binding | AF245436 | coiled-coil domain containing 82 | 0.50 | 0.00036 | 0.0084 |
| MYCBP2 | protein binding | AK000968 | MYC binding protein 2 | 1.27 | 0.00036 | 0.0085 |
| SPARC | ossification | NM_003118 | secreted protein, acidic, cysteine-rich (osteonectin) | 0.57 | 0.00036 | 0.0085 |
| CARD11 | positive regulation of cytokine production | AF322641 | caspase recruitment domain family, member 11 | 1.48 | 0.00037 | 0.0085 |
| DNMT1 | negative regulation of transcription from RNA polymerase II promoter | NM_001379 | DNA (cytosine-5-)-methyltransferase 1 | 1.57 | 0.00037 | 0.0085 |
| HELZ | nucleotide binding | NM_014877 | helicase with zinc finger | 1.55 | 0.00039 | 0.0088 |
| RSPRY1 | protein binding | AI206557 | ring finger and SPRY domain containing 1 | 0.76 | 0.00039 | 0.0089 |
| EVL | actin binding | NM_016337 | Enah/Vasp-like | 1.29 | 0.00041 | 0.0091 |
| SLC35D2 | nucleotide-sugar transmembrane transporter activity | AJ005866 | solute carrier family 35, member D2 | 0.82 | 0.00042 | 0.0093 |
| MYADM | membrane | BE908995 | myeloid-associated differentiation marker | 1.84 | 0.00042 | 0.0094 |
| ERP27 | endoplasmic reticulum | AI051248 | endoplasmic reticulum protein 27 kDa | 0.83 | 0.00043 | 0.0095 |
| SLC12A9 | transport | NM_020246 | solute carrier family 12 (potassium/chloride transporters), member 9 | 0.79 | 0.00044 | 0.0095 |
| GCHFR | protein binding | NM_005258 | GTP cyclohydrolase I feedback regulator | 0.67 | 0.00045 | 0.0097 |
| CSNK2A2 | nucleotide binding | AI022089 | casein kinase 2, alpha prime polypeptide | 1.23 | 0.00045 | 0.0098 |
| MVK | nucleotide binding | M88468 | mevalonate kinase | 1.26 | 0.00045 | 0.0098 |
| OS9 | protein binding | NM_006812 | amplified in osteosarcoma | 1.33 | 0.00046 | 0.0098 |

^a^: Genes with P Values < 0.01 were sorted by P Values in an ascending order.

^b^: Gene ontology (GO) annotations were obtained by the GOstat program (http://gostat.wehi.edu.au/), and the first annotation was shown when a gene has multiple GO annotations. Missing value was presented as “---”.

^c^: FC, denoting fold change, is defined as the ratio of the expression value of positive over negative responders on the absolute scale.

^d^: P Value was calculated using the ordinary Student’s *t* test for each gene.

^e^: FDR, denoting false discovery rate, was estimated by the Benjamini-Hochberg (BH) method.

#### Table S3. Differentially Expressed Gene List for Week 12 Topiramate Group Between Responders (*n* = 6) and Non-responders (*n* = 11) ^a^

|  |  |  |  |  |  |  |
| --- | --- | --- | --- | --- | --- | --- |
| Gene Symbol | GO Annotation^b^ | GenBank  Acc. Number | Gene Name | FC^c^ | P Value^d^ | FDR^e^ |
|  |  |  |  |
|  |  |  |  |  |  |  |
|  |  |  |  |  |  |  |
| ITCH | regulation of cell growth | AB056663 | itchy E3 ubiquitin protein ligase homolog (mouse) | 0.72 | 1.00×10^-6^ | 0.0033 |
| MKNK2 | nucleotide binding | NM_017572 | MAP kinase interacting serine/threonine kinase 2 | 0.77 | 8.00×10^-6^ | 0.013 |
| RPS17 | RNA binding | NM_001021 | ribosomal protein S17 | 1.54 | 1.70×10^-5^ | 0.019 |
| PPIE | nucleotide binding | NM_006112 | peptidylprolyl isomerase E (cyclophilin E) | 1.31 | 4.40×10^-5^ | 0.029 |
| SEC14L1 | transporter activity | AI017770 | SEC14-like 1 (S. cerevisiae) | 0.65 | 4.90×10^-5^ | 0.029 |
| SKP1 | protein binding | BE964043 | S-phase kinase-associated protein 1 | 1.32 | 5.20×10^-5^ | 0.029 |
| KATNB1 | spindle pole | NM_005886 | katanin p80 (WD repeat containing) subunit B 1 | 0.77 | 6.30×10^-5^ | 0.030 |
| ZCCHC17 | RNA binding | AF151085 | zinc finger, CCHC domain containing 17 | 1.30 | 0.00010 | 0.042 |
| ZDHHC18 | zinc ion binding | BG168720 | zinc finger, DHHC-type containing 18 | 0.75 | 0.00012 | 0.045 |
| WDR68 | protein binding | AA769995 | WD repeat domain 68 | 1.50 | 0.00014 | 0.045 |
|  |  |  |  |  |  |  |

^a^: Genes with P Values < 0.01 were sorted by P Values in an ascending order.

^b^: Gene ontology (GO) annotations were obtained by the GOstat program (http://gostat.wehi.edu.au/), and the first annotation was shown when a gene has multiple GO annotations. Missing value was presented as “---”.

^c^: FC, denoting fold change, is defined as the ratio of the expression value of positive over negative responders on the absolute scale.

^d^: P Value was calculated using the ordinary Student’s *t* test for each gene.

^e^: FDR, denoting false discovery rate, was estimated by the Benjamini-Hochberg (BH) method.

#### Table S4. Differentially Expressed Gene List for Week 12 Placebo Group Between Responders (*n* = 2) and Non-responsers (*n* = 13) ^a^

|  |  |  |  |  |  |  |
| --- | --- | --- | --- | --- | --- | --- |
| Gene Symbol | GO Annotation^b^ | GenBank  Acc. Number | Gene Name | FC^c^ | P Value^d^ | FDR^e^ |
|  |  |  |  |
|  |  |  |  |  |  |  |
|  |  |  |  |  |  |  |
| UBE2O | small conjugating protein ligase activity | NM_022066 | ubiquitin-conjugating enzyme E2O | 3.00 | <1.00×10^-6^ | <1.00×10^-5^ |
| EPB41 | actin binding | AA522435 | erythrocyte membrane protein band 4.1 (elliptocytosis 1, RH-linked) | 1.97 | <1.00×10^-6^ | <1.00×10^-5^ |
| SLC41A1 | magnesium ion binding | AW439816 | solute carrier family 41, member 1 | 1.45 | <1.00×10^-6^ | <1.00×10^-5^ |
| TXNRD2 | response to oxygen radical | AB019695 | thioredoxin reductase 2 | 0.84 | 1.00×10^-6^ | 0.00038 |
| GORASP1 | protein binding | AK001574 | golgi reassembly stacking protein 1, 65kDa | 1.31 | 1.00×10^-6^ | 0.00038 |
| HAB1 | --- | AJ006206 | B1 for mucin | 0.62 | 1.00×10^-6^ | 0.00038 |
| COPZ1 | Golgi membrane | NM_016057 | coatomer protein complex, subunit zeta 1 | 0.83 | 1.00×10^-6^ | 0.00038 |
| GNA12 | nucleotide binding | BG028884 | guanine nucleotide binding protein (G protein) alpha 12 | 1.95 | 1.00×10^-6^ | 0.00038 |
| RANBP10 | --- | AL041852 | RAN binding protein 10 | 1.93 | 1.00×10^-6^ | 0.00038 |
| GOLGA2 | protein binding | AL514295 | golgi autoantigen, golgin subfamily a, 2 | 1.44 | 2.00×10^-6^ | 0.00068 |
| THOC2 | RNA binding | BE543527 | THO complex 2 | 2.05 | 3.00×10^-6^ | 0.00093 |
| DNAJA4 | protein folding | N45231 | DnaJ (Hsp40) homolog, subfamily A, member 4 | 1.57 | 4.00×10^-6^ | 0.0011 |
| CASP6 | protein binding | U20537 | caspase 6, apoptosis-related cysteine peptidase | 0.58 | 6.00×10^-6^ | 0.0014 |
| MLL | transcription factor activity | AI701430 | myeloid/lymphoid or mixed-lineage leukemia (trithorax homolog, Drosophila) | 0.69 | 6.00×10^-6^ | 0.0014 |
| TUBB1 | nucleotide binding | N63244 | tubulin, beta 1 | 2.18 | 6.00×10^-6^ | 0.0014 |
| DVL3 | signal transducer activity | NM_004423 | dishevelled, dsh homolog 3 (Drosophila) | 1.53 | 7.00×10^-6^ | 0.0014 |
| TES | zinc ion binding | NM_015641 | testis derived transcript (3 LIM domains) | 1.31 | 7.00×10^-6^ | 0.0014 |
| ARHGAP24 | angiogenesis | AI743534 | Rho GTPase activating protein 24 | 1.71 | 9.00×10^-6^ | 0.0017 |
| CLCN3 | Golgi membrane | AI760629 | Chloride channel 3 | 1.74 | 1.00×10^-5^ | 0.0018 |
| RNF144A | protein binding | NM_014746 | ring finger protein 144A | 1.40 | 1.20×10^-5^ | 0.0019 |
| FEM1A | receptor activity | BC004988 | fem-1 homolog a (C. elegans) | 1.49 | 1.20×10^-5^ | 0.0019 |
| RPL7 | RNA binding | BG389744 | ribosomal protein L7 | 0.71 | 1.50×10^-5^ | 0.0022 |
| COX15 | catalytic activity | NM_004376 | COX15 homolog, cytochrome c oxidase assembly protein (yeast) | 0.79 | 1.50×10^-5^ | 0.0022 |
| WDR7 | serine-type endopeptidase activity | AB011113 | WD repeat domain 7 | 1.73 | 2.20×10^-5^ | 0.0027 |
| SRRM1 | DNA binding | NM_005839 | serine/arginine repetitive matrix 1 | 1.53 | 2.30×10^-5^ | 0.0027 |
| ENC1 | actin binding | NM_003633 | ectodermal-neural cortex (with BTB-like domain) | 1.63 | 2.30×10^-5^ | 0.0027 |
| LARS | nucleotide binding | NM_020117 | leucyl-tRNA synthetase | 0.71 | 2.30×10^-5^ | 0.0027 |
| ZNF148 | negative regulation of transcription from RNA polymerase II promoter | AW594167 | zinc finger protein 148 | 1.32 | 2.30×10^-5^ | 0.0027 |
| RASGRP2 | guanyl-nucleotide exchange factor activity | AI688812 | RAS guanyl releasing protein 2 (calcium and DAG-regulated) | 0.78 | 2.40×10^-5^ | 0.0027 |
| EHBP1L1 | --- | BG334196 | EH domain binding protein 1-like 1 | 0.70 | 2.40×10^-5^ | 0.0027 |
| EIF3B | nucleotide binding | NM_003751 | eukaryotic translation initiation factor 3, subunit B | 1.43 | 2.50×10^-5^ | 0.0027 |
| EIF2AK1 | nucleotide binding | NM_014413 | eukaryotic translation initiation factor 2-alpha kinase 1 | 1.83 | 2.50×10^-5^ | 0.0027 |
| CSNK1G1 | nucleotide binding | BG104860 | casein kinase 1, gamma 1 | 1.39 | 2.80×10^-5^ | 0.0029 |
| DENND4A | DNA binding | BC041706 | DENN/MADD domain containing 4A | 1.71 | 3.10×10^-5^ | 0.0030 |
| MFHAS1 | protein binding | BE783723 | malignant fibrous histiocytoma amplified sequence 1 | 1.29 | 3.10×10^-5^ | 0.0030 |
| BCL2L1 | release of cytochrome c from mitochondria | AL117381 | BCL2-like 1 | 2.14 | 3.30×10^-5^ | 0.0031 |
| DLEU2 | cell cycle | H48516 | deleted in lymphocytic leukemia 2 (non-protein coding) | 0.63 | 3.40×10^-5^ | 0.0031 |
| ECD | transcription coactivator activity | NM_007265 | ecdysoneless homolog (Drosophila) | 1.45 | 3.80×10^-5^ | 0.0034 |
| GPR155 | intracellular signaling cascade | AI970061 | G protein-coupled receptor 155 | 0.54 | 3.90×10^-5^ | 0.0034 |
| FBXW12 | ubiquitin cycle | AK022174 | F-box and WD repeat domain containing 12 | 0.65 | 4.70×10^-5^ | 0.0040 |
| PRR11 | --- | AK000296 | proline rich 11 | 0.72 | 5.00×10^-5^ | 0.0042 |
| SGPL1 | endoplasmic reticulum | AB033078 | sphingosine-1-phosphate lyase 1 | 1.30 | 5.60×10^-5^ | 0.0045 |
| GNL1 | nucleotide binding | NM_005275 | guanine nucleotide binding protein-like 1 | 0.76 | 5.80×10^-5^ | 0.0045 |
| FHL1 | GO:0003674 molecular_function | NM_001449 | four and a half LIM domains 1 | 1.56 | 5.90×10^-5^ | 0.0045 |
| CCDC152 | --- | AI151104 | coiled-coil domain containing 152 | 0.57 | 6.00×10^-5^ | 0.0045 |
| TRIM68 | protein binding | NM_018073 | tripartite motif-containing 68 | 1.51 | 6.40×10^-5^ | 0.0047 |
| STAU1 | double-stranded RNA binding | BC000830 | staufen, RNA binding protein, homolog 1 (Drosophila) | 1.40 | 7.30×10^-5^ | 0.0053 |
| ZNF592 | DNA binding | BF593899 | zinc finger protein 592 | 1.41 | 7.60×10^-5^ | 0.0053 |
| VPS45 | Golgi membrane | AF165513 | vacuolar protein sorting 45 homolog (S. cerevisiae) | 1.25 | 7.70×10^-5^ | 0.0053 |
| SF3A1 | nuclear mRNA 3'-splice site recognition | AI655996 | splicing factor 3a, subunit 1, 120kDa | 1.87 | 7.80×10^-5^ | 0.0053 |
| MAP3K5 | MAPKKK cascade | NM_005923 | mitogen-activated protein kinase kinase kinase 5 | 1.35 | 8.10×10^-5^ | 0.0054 |
| IRF2BP2 | protein binding | BF968057 | interferon regulatory factor 2 binding protein 2 | 1.41 | 8.80×10^-5^ | 0.0056 |
| GCLC | magnesium ion binding | NM_001498 | glutamate-cysteine ligase, catalytic subunit | 1.56 | 9.00×10^-5^ | 0.0056 |
| LSM12 | --- | AW873564 | LSM12 homolog (S. cerevisiae) | 1.25 | 9.00×10^-5^ | 0.0056 |
| ANKRD40 | --- | AV712687 | ankyrin repeat domain 40 | 1.39 | 9.10×10^-5^ | 0.0056 |
| RPS25 | RNA binding | AA888388 | ribosomal protein S25 | 0.76 | 9.50×10^-5^ | 0.0057 |
| MSRA | protein modification process | NM_012331 | methionine sulfoxide reductase A | 1.44 | 9.50×10^-5^ | 0.0057 |
| URM1 | ubiquitin cycle | NM_030914 | ubiquitin related modifier 1 homolog (S. cerevisiae) | 0.81 | 0.00010 | 0.0059 |
| GLRX5 | mitochondrion | AA133341 | glutaredoxin 5 | 1.96 | 0.00010 | 0.0059 |
| RNASEN | double-stranded RNA binding | NM_013235 | ribonuclease type III, nuclear | 1.50 | 0.00011 | 0.0060 |
| APRT | adenine binding | AA927724 | adenine phosphoribosyltransferase | 0.74 | 0.00011 | 0.0062 |
| ADD1 | actin binding | BE898639 | adducin 1 (alpha) | 1.43 | 0.00011 | 0.0063 |
| GIGYF2 | --- | AB014542 | GRB10 interacting GYF protein 2 | 1.36 | 0.00012 | 0.0064 |
| PRPS1 | magnesium ion binding | BC001605 | phosphoribosyl pyrophosphate synthetase 1 | 1.46 | 0.00013 | 0.0065 |
| RPL6 | DNA binding | NM_000970 | ribosomal protein L6 | 0.74 | 0.00013 | 0.0065 |
| RALBP1 | GTPase activator activity | NM_006788 | ralA binding protein 1 | 1.36 | 0.00013 | 0.0065 |
| CEP110 | protein binding | NM_007018 | centrosomal protein 110kDa | 1.36 | 0.00013 | 0.0065 |
| TFG | signal transducer activity | NM_006070 | TRK-fused gene | 1.34 | 0.00013 | 0.0065 |
| RPS17L4 | --- | AL049693 | ribosomal protein S17-like 4 | 0.54 | 0.00013 | 0.0065 |
| ZNF264 | DNA binding | BF063192 | zinc finger protein 264 | 1.58 | 0.00014 | 0.0066 |
| INO80E | --- | AA743390 | INO80 complex subunit E | 0.80 | 0.00015 | 0.0069 |
| BTBD11 | protein binding | BF510581 | BTB (POZ) domain containing 11 | 1.30 | 0.00015 | 0.0069 |
| ALS2CR2 | nucleotide binding | AB038950 | amyotrophic lateral sclerosis 2 (juvenile) chromosome region, candidate 2 | 2.52 | 0.00015 | 0.0069 |
| XPO4 | binding | BF968638 | exportin 4 | 1.43 | 0.00015 | 0.0069 |
| STAG1 | protein binding | AI126490 | stromal antigen 1 | 1.36 | 0.00016 | 0.0070 |
| OSBPL5 | cytosol | AW271225 | oxysterol binding protein-like 5 | 0.76 | 0.00017 | 0.0077 |
| ATXN1L | --- | AW138861 | ataxin 1-like | 1.34 | 0.00019 | 0.0082 |
| GARNL1 | GTPase activator activity | BG436400 | GTPase activating Rap/RanGAP domain-like 1 | 1.46 | 0.00020 | 0.0086 |
| FOXJ2 | transcription factor activity | NM_018416 | forkhead box J2 | 1.20 | 0.00020 | 0.0087 |
| SLC35E1 | transport | NM_024881 | solute carrier family 35, member E1 | 0.62 | 0.00022 | 0.0092 |
|  |  |  |  |  |  |  |

^a^: Genes with P Values < 0.01 were sorted by P Values in an ascending order.

^b^: Gene ontology (GO) annotations were obtained by the GOstat program (http://gostat.wehi.edu.au/), and the first annotation was shown when a gene has multiple GO annotations. Missing value was presented as “---”.

^c^: FC, denoting fold change, is defined as the ratio of the expression value of positive over negative responders on the absolute scale.

^d^: P Value was calculated using the ordinary Student’s *t* test for each gene.

^e^: FDR, enoting false discovery rate, was estimated by the Benjamini-Hochberg (BH) method.
